# Sparse graphs using exchangeable random measures

**DOI:** 10.1111/rssb.12233

**Published:** 2017-09-23

**Authors:** François Caron, Emily B. Fox

**Affiliations:** ^1^ University of Oxford UK; ^2^ University of Washington Seattle USA

**Keywords:** Exchangeability, Generalized gamma process, Lévy measure, Point process, Random graphs

## Abstract

Statistical network modelling has focused on representing the graph as a discrete structure, namely the adjacency matrix. When assuming exchangeability of this array—which can aid in modelling, computations and theoretical analysis—the Aldous–Hoover theorem informs us that the graph is necessarily either dense or empty. We instead consider representing the graph as an exchangeable *random measure* and appeal to the Kallenberg representation theorem for this object. We explore using completely random measures (CRMs) to define the exchangeable random measure, and we show how our CRM construction enables us to achieve sparse graphs while maintaining the attractive properties of exchangeability. We relate the sparsity of the graph to the Lévy measure defining the CRM. For a specific choice of CRM, our graphs can be tuned from dense to sparse on the basis of a single parameter. We present a scalable Hamiltonian Monte Carlo algorithm for posterior inference, which we use to analyse network properties in a range of real data sets, including networks with hundreds of thousands of nodes and millions of edges.

## 1 Introduction

The rapid increase in the availability and importance of network data has been a driving force behind the significant recent attention on random‐graph models. In devising such models, there are several competing forces:

- *flexibility* to capture network features like sparsity of connections between nodes, heavy‐tailed node degree distributions, dense spots or block structure;
- *interpretability* of the network model and associated parameters;
- *theoretical tractability* of analysis of network properties;
- *computational tractability* of inference with the ability to scale analyses to large collections of nodes.

A plethora of network models have been proposed in recent decades, each with different trade‐offs made between flexibility, interpretability and theoretical and computational tractability; we refer the interested reader to overviews of such models provided by Newman (2003, 2010), Bollobás (2001), Durrett (2007), Goldenberg *et al*. (2010) and Fienberg (2012). In this paper, our focus is on providing a new framework in which to make these trade‐offs. We demonstrate the ability to make gains in multiple directions using this framework through a specific example where the goal is to capture

- *sparsity*—tunable from sparse to dense via interpretable parameters,
- *heavy‐tailed degree distributions*—again controlled via interpretable parameters—and
- *computational tractability* of Bayesian inference, scaling to networks with hundreds of thousands of nodes and millions of edges.

Classically, the graph being modelled has been represented by a discrete structure, or *adjacency matrix*,* Z* where *Z*_*ij*_ is a binary variable with *Z*_*ij*_=1 indicating an edge from node *i* to node *j*. In the case of undirected graphs, we furthermore restrict *Z*_*ij*_=*Z*_*ji*_. Then, the statistical network model is devised with this structure representing the observable quantity.

From a modelling, computational and theoretical standpoint, making an assumption of *exchangeability* is attractive. Under the adjacency matrix graph representation, such a statement informally equates with an invariance in distribution to permutations of node orderings. One reason why this assumption is attractive can be seen from applying the celebrated Aldous–Hoover theorem (Aldous, 1981; Hoover, 1979) to the adjacency matrix: infinite exchangeability of the binary matrix implies a mixture model representation involving transformations of uniform random variables (see theorem 7 in Appendix A.1). For undirected graphs, this transformation is specified by the *graphon* (Borgs *et al*. 2008; Lovász, 2013), which was originally studied as the limit object of dense graph sequences (Lovász and Szegedy, 2006; Borgs *et al*. 2010). The connection with the Aldous–Hoover theorem was made by Diaconis and Janson (2008). The graphon provides an object by which to study the theoretical properties of the statistical network process and to devise new estimators, as has been studied extensively in recent years (Bickel and Chen, 2009; Bickel *et al*., 2011; Rohe, *et al*., 2011; Zhao *et al*., 2012; Airoldi *et al*., 2013; Choi and Wolfe, 2014). Furthermore, the mixture model is a cornerstone of Bayesian modelling and provides a framework in which computational strategies are straightforwardly devised. Indeed, the Aldous–Hoover constructive definition has motivated new models (Lloyd *et al*., 2012) and many popular existing models can be recast in this framework, including the stochastic block model (Nowicki and Snijders, 2001; Airoldi *et al*., 2008) and latent space model (Hoff *et al*., 2002).

One consequence of the Aldous–Hoover theorem is that graphs that are represented by an exchangeable random array are either empty or dense, i.e. the number of edges grows quadratically with the number of nodes *n* (see Lovász (2013) and Orbanz and Roy (2015)). However, empirical analyses suggest that many real world networks are sparse (Newman, 2010). Formally, sparsity is an asymptotic property of a graph. Following Bollobás and Riordan (2009), we refer to graphs with *Θ*(*n* ^2^) edges as *dense* and graphs with *o*(*n* ^2^) edges as *sparse* (for notation, see Appendix C). The conclusion appears to be that we cannot have both exchangeability, with the associated benefits described above, and sparse graphs. Although network models can often adapt parameters to fit finite graphs, it is appealing to have a modelling framework with theoretically provable properties that are consistent with observed network attributes.

There are a couple of approaches to handling this apparent issue. One is to give up on exchangeability to obtain sparse graphs, such as in the popular preferential attachment model (Barabási and Albert, 1999; Berger *et al*., 2014) or configuration model (Bollobás, 1980; Newman, 2010). Indeed, the networks literature is dominated by sparse non‐exchangeable models. Alternatively, there is a body of literature that examines rescaling graph properties with network size *n*, leading to sparse graph sequences where each graph is finitely exchangeable (Bollobás *et al*., 2007; Bollobás and Riordan, 2009; Olhede and Wolfe, 2012; Wolfe and Olhede, 2013; Borgs *et al*., 2014a,b). Convergence of sparse graph sequences, analogous to the study of limiting objects for dense graph sequences, has also been studied (e.g. Borgs *et al*. (2017)). However, any method building on a rescaling approach provides a graph distribution *π*_*n*_ that lacks projectivity: marginalizing node *n* does not yield *π*_*n*−1_, the distribution on graphs of size *n*−1.

**Figure 1:**
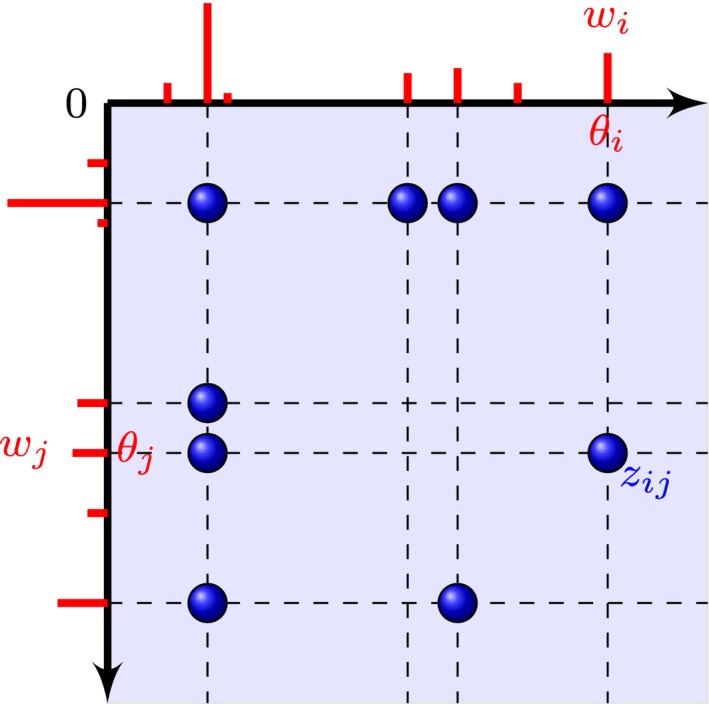
Point process representation of a random graph: each node *i* is embedded in $R_{+}$ at some location $\theta_{i}$ and is associated with a sociability parameter *w*_*i*_; an edge between nodes $\theta_{i}$ and $\theta_{j}$ is represented by a point at locations $\left(\theta_{i},\theta_{j}\right)$ and $\left(\theta_{j},\theta_{i}\right)$ in $R_{+}^{2}$

We instead propose to set aside the discrete structure of the adjacency matrix and examine a different notion of exchangeability for a continuous space representation of networks. In particular, we consider a *point process* on $R_{+}^{2}$:

(1) $$Z=\sum_{i,j}z_{ij}\delta_{\left(\theta_{i},\theta_{j}\right)},$$

where *z*_*ij*_ is 1 if there is a link between node *i* and node *j*, and is 0 otherwise, and $\theta_{i}$ and $\theta_{j}$ are in $R_{+}=\left[\right.0,\infty )$ (Fig. 1). We can think of $\theta_{i}$ as a *time index* for node *i*. Exchangeability, as defined in Section 2, then equates with invariance to the time of arrival of the nodes. See Section 3.5 for a further interpretation of $\theta_{i}$.

We note that exchangeability of the point process representation does not imply exchangeability of the associated adjacency matrix; however, the same modelling, computational and theoretical advantages remain. Interestingly, we arrive at a direct analogue to the constructive representation of the Aldous–Hoover theorem for exchangeable arrays and the associated graphon. Appealing to Kallenberg (1990, 2005), chapter 9, a point process on $R_{+}^{2}$ is exchangeable if and only if it can be represented as a transformation of unit rate Poisson processes and uniform random variables (see theorem 1 in Section 2).

As a case‐study in how this exchangeable random‐measure framework can enable statistical network models with properties that are different from what can be achieved in the exchangeable array framework, we consider the following specification. To induce node heterogeneity in the link probabilities, we endow each node with a scalar *sociability parameterw*_*i*_>0. We then consider a straightforward link probability model. For any *i*≠*j*,

(2) $$\text{Pr}\left(z_{ij}=1|w_{i},w_{j},\theta_{i},\theta_{j}\right)=1-\exp\left(-2w_{i}w_{j}\right).$$

This link function has been previously used by several others to build network models (Aldous, 1997; Norros and Reittu, 2006; Bollobás, *et al*., 2007; van der Hofstad, 2014). Note that, under this specification, the ‘time indices’ $\theta_{i}$ and *θ*_*j*_ of nodes *i* and *j* do not influence the probability of these two nodes to form a link. This is in contrast with, for example, standard latent space models (Hoff *et al*., 2002). See Section 4 for further discussion.

To define the set of ${\left(w_{i},\theta_{i}\right)}_{i=1,2,\ldots }$ underlying this statistical network model, we explore the use of *completely random measures* (CRMs) (Kingman, 1967). The (*w*_*i*_)_*i*=1,2,…_ are the jumps of the CRM and the ${\left(\theta_{i}\right)}_{i=1,2,\ldots }$ the locations of the atoms. We show that, by carefully choosing the Lévy measure characterizing this CRM, we can construct graphs ranging from *sparse* to *dense*. In particular, any Lévy measure yielding an *infinite activity* CRM leads to sparse graphs; alternatively, *finite activity* CRMs yield dense graphs. For the class of infinite activity *regularly varying* CRMs, we can sharpen the results to obtain graphs where the number of edges increases at a rate below *n* ^*a*^, where 1<*a*<2 depending on the Lévy measure. We focus on the flexible *generalized gamma process* CRM and show that one can tune the graph from dense to sparse via a single parameter.

Building on the framework of CRMs leads to other desirable properties as well. One is that our CRM‐based exchangeable point process leads to an analytic representation for the graphon analogue in the Kallenberg framework (see Section 5.1). Another is that, by drawing on the considerable theory of CRMs that has been well studied in the Bayesian non‐parametric community, we can derive network simulation techniques and develop a principled statistical estimation procedure. For the latter, in Section 7 we devise a scalable Hamiltonian Monte Carlo (HMC) sampler that can automatically handle a range of graphs from dense to sparse. We empirically show in Section 8 that our methods scale to graphs with hundreds of thousands of nodes and millions of edges. Importantly, exchangeability of the random measure underlies the efficiency of the sampler.

In summary, the CRM‐based formulation combined with the specific link model of equation (2) serves as a proof of concept that moving to the point process representation of equation (1) can yield models with desirable attributes that are different from what can be obtained by using the discrete adjacency matrix representation. More generally, the notion of modelling the graph as an exchangeable random measure and appealing to a Kallenberg representation for such exchangeable random measures serves as an important new framework for devising and studying random‐graph models, just as the original graphon concept stimulated considerable work in the network community in the past decade.

Our paper is organized as follows. In Section 2, we provide background on exchangeability and CRMs. Our statistical network models for directed multigraphs, undirected graphs and bipartite graphs are in Section 3. A discussion of our framework compared with related network models is provided in Section 4. Properties, such as exchangeability and sparsity, and methods for simulation are presented in Section 5. Specific cases of our formulation leading to dense and sparse graphs are considered in Section 6, including an empirical analysis of network properties of our proposed formulation. Our Markov chain Monte Carlo (MCMC) posterior computations are in Section 7. Finally, Section 8 provides a simulation study and an analysis of a variety of large, real world graphs.

The programs that were used to analyse the data can be obtained from

http://wileyonlinelibrary.com/journal/rssdatasets

## 2 Background on exchangeability

Our focus is on exchangeable random structures that can represent networks. We first briefly review exchangeability for random sequences, continuous time processes and discrete network arrays. Thorough and accessible overviews of exchangeability of random structures have been presented in the surveys of Aldous (1985) and Orbanz and Roy (2015). Here, we simply abstract away the notions that are relevant to placing our network formulation in context, as summarized in Table 1.

**Table 1:** Overview of representation theorems

	*Discrete structure*	*Continuous time or space*
Exchangeability	de Finetti (1931)	Bühlmann (1960)
Joint or separate	Aldous (1981) and	Kallenberg (1990)
exchangeability	Hoover (1979)	

The classical representation theorem arising from a notion of exchangeability for discrete *sequences* of random variables is due to de Finetti (1931). The theorem states that a sequence *Z*_1_,*Z*_2_,… with $Z_{i}\in Z$ is exchangeable if and only if there is a random probability measure P on **Z** with law *ν* such that the *Z*_*i*_ are conditionally independently and identically distributed (IID) given P, i.e. all exchangeable infinite sequences can be represented as a mixture with mixing measure *ν*. If examining continuous time *processes* instead of sequences, the representation that is associated with exchangeable *increments* was given by Bühlmann (1960) (see also Freedman (1996) in terms of mixing Lévy processes.

The focus of our work, however, is on graph structures. For generic matrices *Z* in some space **Z**, an (infinite) *exchangeable random array* (Diaconis and Janson, 2008; Lauritzen, 2008) is one such that

(3) $$\left(Z_{ij}\right)\overset{d}{=}\;\left(Z_{\pi \left(i\right)\tilde{}\pi \left(j\right)}\right)\;\text{for}\;\left(i,j\right)\in N^{2}$$

for any permutations $\pi ,\tilde{}\pi$ of N (separate exchangeability), or for any permutation $\pi =\tilde{}\pi$ of N (joint exchangeability), where the notation ‘=^d^’ stands for ‘equal in distribution’. A representation theorem for exchangeability of the classical discrete adjacency *matrix Z* arises by considering a special case of the Aldous–Hoover theorem (Aldous, 1981; Hoover, 1979) to *2‐arrays*. For undirected graphs where *Z* is a binary, symmetric adjacency matrix, the Aldous–Hoover representation can be expressed as the existence of a *graphon*. For completeness, the Aldous–Hoover theorem (specialized to 2‐arrays under joint exchangeability) and further details on the graphon are provided in Appendix A.1.

Throughout this paper, we instead consider representing a graph as a point process $Z=\Sigma_{i,j}z_{ij}\delta_{\left(\theta_{i},\theta_{j}\right)}$ with nodes $\theta_{i}$ embedded in $R_{+}$, as in equation (1), and then examine notions of exchangeability in this context. Paralleling result (3), the point process *Z* on $R_{+}^{2}$ is exchangeable if and only if (Kallenberg (2005), chapter 9)

(4) $$\left(Z\left(A_{i}\times A_{j}\right)\right)\overset{d}{=}\;\left(Z\left(A_{\pi (i)}\times A_{\tilde{}\pi \left(j\right)}\right)\right)\;\text{for}\;\left(i,j\right)\in N^{2},$$

for any permutations $\pi ,\tilde{\pi }$ of N, with $\pi =\tilde{\pi }$ in the jointly exchangeable case, and any *intervalsA*_*i*_=[*h*(*i*−1),*hi*] with i∈N and *h*>0.

**Figure 2:**
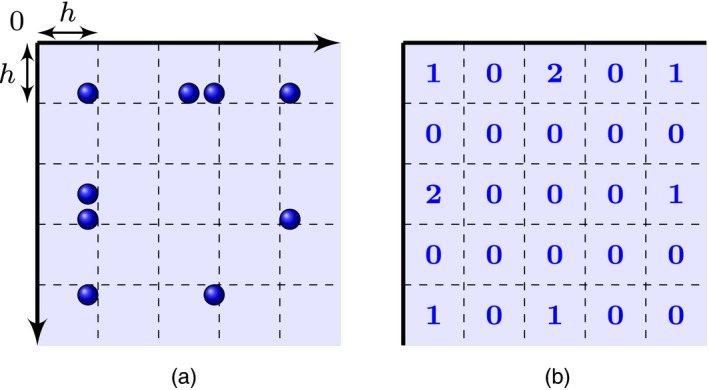
Illustration of the notion of exchangeability for point processes on the plane: for any regular square grid on the plane (a), the associated infinite array counting the number of points in each square (b) is exchangeable in the sense of result (3)

In words, result (4) states that the point process *Z* is exchangeable if, for any arbitrary regular square grid on the plane, the associated infinite array of increments (edge counts between nodes in a square) is exchangeable (Fig. 2). This provides a notion of exchangeability akin to that of the Aldous–Hoover theorem, but fundamentally different as the array being considered here is formed on the basis of an underlying continuous process. This array is not equivalent to an adjacency matrix, regardless of how fine a grid is considered.

Kallenberg (1990) derived de‐Finetti‐style representation theorems for separately and jointly exchangeable random measures on $R_{+}^{2}$, which we present for the jointly exchangeable case in theorem 1. In what follows *λ* denotes the Lebesgue measure on $R_{+}$, *λ*_*D*_ the Lebesgue measure on the diagonal $D=\{\left(s,t\right)\in R_{+}^{2}|s=t\}$ and ${\tilde{N}}_{2}=\left\{\left\{i,j\right\}|\left(i,j\right)\in N^{2}\right\}$. We also define a *U‐array* to be an array of independent uniform random variables.

Theorem 1 (representation theorem for jointly exchangeable random measures on $R_{+}^{2}$ (Kallenberg (1990) and Kallenberg (2005), theorem 9.24)). A random measure *ξ* on $R_{+}^{2}$ is jointly exchangeable if and only if almost surely

(5) $$\begin{array}{cc} \xi = & \sum_{i,j}f\left(\alpha_{0},\vartheta_{i},\vartheta_{j},\zeta_{\left\{i,j\right\}}\right)\delta_{\theta_{i},\theta_{j}}+\beta_{0}\lambda_{D}+\gamma_{0}\left(\lambda \times \lambda \right)\\ & +\sum_{j,k}\left\{g\left(\alpha_{0},\vartheta_{j},\chi_{jk}\right)\delta_{\theta_{j},\sigma_{jk}}+g^{\prime }\left(\alpha_{0},\vartheta_{j},\chi_{jk}\right)\delta_{\sigma_{jk},\theta_{j}}\right\}\\ & +\sum_{j}\left\{h\left(\alpha_{0},\vartheta_{j}\right)\left(\delta_{\theta_{j}}\times \lambda \right)+h^{\prime }\left(\alpha_{0},\vartheta_{j}\right)\left(\lambda \times \delta_{\theta_{j}}\right)\right\}\\ & +\sum_{k}\left\{l\left(\alpha_{0},\eta_{k}\right)\delta_{\rho_{k},\rho_{k}^{\prime }}+l^{\prime }\left(\alpha_{0},\eta_{k}\right)\delta_{\rho_{k}^{\prime },\rho_{k}}\right\} \end{array}$$

for some measurable functions $f:R_{+}^{4}\to R_{+}$, $g,g^{\prime }:R_{+}^{3}\to R_{+}$ and *h*,*h* ^′^,*l*,*l* ^′^: $R_{+}^{2}\to R_{+}$. Here, (*ζ*_{*i*,*j*}_) with $\left\{i,j\right\}\in {\tilde{N}}_{2}$ is a U‐array. $\left\{\left(\theta_{j},\vartheta_{j}\right)\right\}$ and {(*σ*_*ij*_,*χ*_*ij*_)} on $R_{+}^{2}$ and $\left\{\left(\rho_{j},\rho_{j}^{\prime },\eta_{j}\right)\right\}$ on $R_{+}^{3}$ are independent, unit rate Poisson processes. Furthermore, *α*_0_,*β*_0_,*γ*_0_⩾0 are an independent set of random variables.

We can think of the $\theta_{i}$ as random time indices, the sets $\left\{\theta_{i}\right\}\times R_{+}$ and $R_{+}\times \left\{\theta_{j}\right\}$ forming Poisson processes of vertical and horizontal lines. The representation (1) is slightly more involved than the representation theorem for exchangeable arrays (see Appendix A.1). The first component of *ξ* is, however, similar to the representation for exchangeable arrays, the sequence of fixed indices *i*=1,2,… and uniform random variables (*U*_*i*_)_*i*=1,2,…_ in equation (46) in Appendix A.1 being replaced by a unit rate Poisson process $\left\{\left(\theta_{i},\vartheta_{i}\right)\right\}$ on $R_{+}^{2}$. We place our proposed network model of Section 3 within this Kallenberg representation in Section 5.1, yielding direct analogues to the classical graphon representation of graphs based on exchangeability of the adjacency matrix.

## 3 Proposed statistical network model

Recall that we represent an undirected graph using an atomic measure

$$Z=\sum_{i=1}^{\infty }\sum_{j=1}^{\infty }z_{ij}\delta_{\left(\theta_{i},\theta_{j}\right)},$$

with the convention $z_{ij}=z_{ji}\in \left\{0,1\right\}$. Here, *z*_*ij*_=*z*_*ji*_=1 indicates an undirected edge between nodes $\theta_{i}$ and *θ*_*j*_. See Section 3.5 for the interpretation of $\theta_{i}$.

There are many options for defining a statistical model for the point process graph representation *Z*. We consider one in particular in this paper based on a specific choice of

- link probability model and
- a prior on the model parameters.

Expanding on the discussion of Section 1, we introduce a collection of per‐node *sociability* parameters *w*={*w*_*i*_} and specify link probabilities via

(6) $$\text{Pr}\left(z_{ij}=1|w\right)=\left\{\begin{array}{cc} 1-\exp(-2w_{i}w_{j}) & i\neq j,\\ 1-\exp(-w_{i}^{2}) & i=j. \end{array}\right.$$

As mentioned in Section 1, this link probability model is not new to the statistical networks community and is a straightforward method for achieving node heterogeneity (see Aldous (1997) and Norros and Reittu (2006)).

### 3.1 Defining node parameters by using completely random measures

The model parameters consist of a collection of node‐specific sociability parameters *w*_*i*_>0 and continuous‐valued node indices $\theta_{i}\in R_{+}$.

Our generative model jointly specifies ${\left(w_{i},\theta_{i}\right)}_{i=1,2,\cdots }$ by first defining an atomic random measure

(7) $$W=\sum_{i=1}^{\infty }w_{i}\delta_{\theta_{i}}$$

and then taking *W* to be distributed according to a homogeneous CRM (Kingman, 1967).

CRMs have been used extensively in the Bayesian non‐parametric literature for proposing flexible classes of priors over functional space (Regazzini *et al*., 2003; Lijoi and Prünster, 2010). We briefly review a few important properties of CRMs that are relevant to our construction; the reader can refer to Kingman (1993) or Daley and Vere‐Jones (2008) for an exhaustive coverage.

A CRM *W* on $R_{+}$ is a random measure such that, for all finite families of disjoint, bounded measurable sets (*A*_1_,…,*A*_*n*_) of $R_{+}$, the random variables *W*(*A*_1_),…,*W*(*A*_*n*_) are mutually independent.

We shall focus here on CRMs with no deterministic component and stationary increments (i.e. the distribution of *W*([*t*,*s*]) depends only on *t*−*s*). In this case, the CRM takes the form (7), with ${\left(w_{i},\theta_{i}\right)}_{i\in N}$ the points of a Poisson point process on $\left(0,\infty \right)\times R_{+}$ defined by a mean measure *ν*(d*w*,d*θ*)=*ρ*(d*w*)*λ*(d*θ*), where *λ* is the Lebesgue measure and *ρ* is a Lévy measure on (0,∞).

We denote this by

(8) W∼CRM(ρ,λ).

Note that *W*([0,*T*])<∞ almost surely for any *T*<∞, whereas $W(R_{+})=\infty$ almost surely if $\int_{0}^{\infty }\rho \left(dw\right)>0$.

The jump part *ρ* of the mean measure—which characterizes the increments of *W*—is of particular interest in our graph construction, as explored in Section 5. If *ρ* satisfies the condition

(9) $$\int_{0}^{\infty }\rho \left(dw\right)=\infty ,$$

then there will be an infinite number of jumps in any interval [0,*T*], and we refer to the CRM as *infinite activity*. Otherwise, the number of jumps will be finite almost surely. In our model, the jumps correspond to potentially connected nodes, i.e. these nodes need not be connected to any other node within a bounded interval and instead represent an upper bound on the set of connected nodes. See Section 3.5 for further discussion.

In Section 6, we consider special cases including the (compound) Poisson process and generalized gamma process (GGP) (Brix, 1999; Lijoi *et al*., 2007).

### 3.2 Directed multigraphs

Formally, our undirected graph model is viewed as a transformation of a directed integer‐weighted graph, or *multigraph*, as detailed in Section 3.3. We now specify this directed multigraph. Although our primary focus is on undirected network models, in some applications the directed multigraph might actually be the direct quantity of interest. For example, in social networks, interactions are often not only directed (‘person *i* messages person *j*’) but also have an associated count. Additionally, interactions might be typed (‘message’, ‘SMS’, ‘like’, ‘tag’). Our proposed framework could be directly extended to model such data.

**Figure 3:**
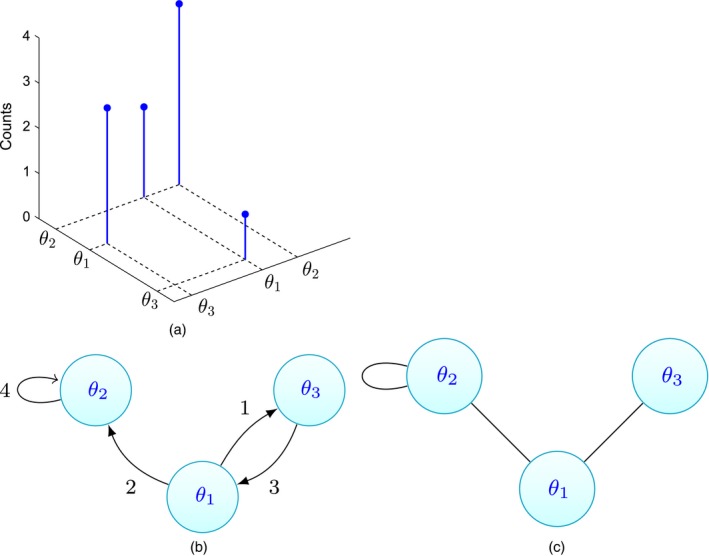
Example of (a) an atomic measure *D* as in equation (10) restricted to [0,1]^2^, (b) the corresponding directed multigraph and (c) the corresponding undirected graph

Let *V*=(*θ*_1_,*θ*_2_,…) be a countably infinite set of node indices with $\theta_{i}\in R_{+}$. We represent the directed multigraph of interest with an atomic measure on $R_{+}^{2}$

(10) $$D=\sum_{i=1}^{\infty }\sum_{j=1}^{\infty }n_{ij}\delta_{\left(\theta_{i},\theta_{j}\right)},$$

where *n*_*ij*_ counts the number of directed edges from node *i* to node *j*, with time indices $\theta_{i}$ and *θ*_*j*_. See Fig. 3 for an illustration.

Given *W* as defined in expressions (7) and (8), *D* is simply generated from a Poisson process with intensity given by the product measure $\tilde{W}=W\times W$ on $R_{+}^{2}$:

(11) $$D|W∼\text{PP}(\tilde{W}),$$

i.e., informally, the individual counts *n*_*ij*_ are generated as Poisson(*w*_*i*_ *w*_*j*_). (We consider a generalized definition of a Poisson process, where the mean measure is allowed to have atoms (Daley and Vere‐Jones (2003), section 2.4).) By construction, for any A,B⊂R, we have $\tilde{W}\left(A\times B\right)=W\left(A\right)W\left(B\right)$. On any bounded interval *A* of $R_{+}$, *W*(*A*)<∞, implying that $\tilde{W}\left(A\times A\right)$ has finite mass.

### 3.3 Undirected graphs via transformations of directed graphs

We arrive at the undirected graph via a simple transformation of the directed graph: set *z*_*ij*_=*z*_*ji*_=1 if *n*_*ij*_+*n*_*ji*_>0 and *z*_*ij*_=*z*_*ji*_=0 otherwise, i.e. place an undirected edge between nodes $\theta_{i}$ and *θ*_*j*_ if and only if there is at least one directed interaction between the nodes. In this definition of an undirected graph, we allow self‐edges. This could represent, for example, a person posting a message on his or her own profile page. The resulting hierarchical model is

(12) $$\left.\begin{array}{c} W=\sum_{i=1}^{\infty }w_{i}\delta_{\theta_{i}}\;\;W∼\text{CRM}\left(\rho ,\lambda \right),\\ D=\sum_{i=1}^{\infty }\sum_{j=1}^{\infty }n_{ij}\delta_{\left(\theta_{i},\theta_{j}\right)}\;\;D|W∼\text{PP}\left(W\times W\right),\\ Z=\sum_{i=1}^{\infty }\sum_{j=1}^{\infty }\min\left(n_{ij}+n_{ji},1\right)\delta_{\left(\theta_{i},\theta_{j}\right)}. \end{array}\right\}$$

**Figure 4:**
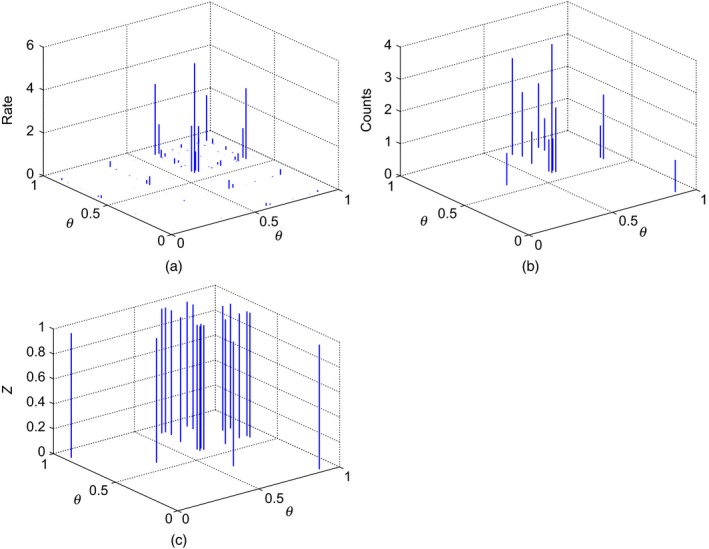
Example of (a) the product measure $\tilde{W}=W\times W$ for CRM *W*, (b) a draw of the directed multigraph measure *D*|*W*∼PP (*W*×*W*) and (c) the corresponding undirected measure $Z=\Sigma_{i=1}^{\infty }\Sigma_{j=1}^{\infty }\min\left(n_{ij}+n_{ji},1\right)\delta_{\left(\theta_{i},\theta_{j}\right)}$

This process is depicted graphically in Fig. 4.

To see the equivalence between this formulation and that specified in equation (6), note that, for *i*≠*j*, $\text{Pr}\left(z_{ij}=1|w\right)=\text{Pr}\left(n_{ij}+n_{ji}>0|w\right)$. By properties of the Poisson process, *n*_*ij*_ and *n*_*ji*_ are independent random variables conditioned on *W*. The sum of two Poisson random variables, each with rate *w*_*i*_ *w*_*j*_, is again Poisson with rate 2*w*_*i*_ *w*_*j*_. Result (6) arises from the fact that $\text{Pr}\left(n_{ij}+n_{ji}>0|w\right)=1-\text{Pr}\left(n_{ij}+n_{ji}=0|w\right)$. Likewise, the *i*=*j* case arises by using a similar reasoning for $\text{Pr}\left(z_{ii}=1|w\right)=\text{Pr}\left(n_{ii}>0|w\right)$.

We note that our computational strategy of Section 7 relies on this interpretation of our model for undirected graphs as a transformation of a directed multigraph. In particular, we introduce the directed edge counts as latent variables and do inference over these counts.

### 3.4 Bipartite graphs

The above construction can also be extended to bipartite graphs. Let *V*=(*θ*_1_,*θ*_2_,…) and $V^{\prime }=\left(\theta_{1}^{\prime },\theta_{2}^{\prime },\ldots \right)$ be two countably infinite sets of nodes with $\theta_{i},\theta_{i}^{\prime }\in R_{+}$. We assume that only connections between nodes of different sets are allowed.

We represent the *directed bipartite multigraph* of interest by using an atomic measure on $R_{+}^{2}$:

(13) $$D=\sum_{i=1}^{\infty }\sum_{j=1}^{\infty }n_{ij}\delta_{\left(\theta_{i},\theta_{j}^{\prime }\right)},$$

where *n*_*ij*_ counts the number of directed edges from node $\theta_{i}$ to node $\theta_{j}^{\prime }$. Similarly, the *bipartite graph* is represented by an atomic measure

$$Z=\sum_{i=1}^{\infty }\sum_{j=1}^{\infty }z_{ij}\delta_{\left(\theta_{i},\theta_{j}^{\prime }\right)}.$$

Our bipartite graph formulation introduces two independent CRMs, *W*∼CRM(*ρ*,*λ*) and *W* ^′^∼CRM(*ρ* ^′^,*λ*), whose jumps correspond to sociability parameters for nodes in sets *V* and *V* ^′^ respectively. The generative model for the bipartite graph mimics that of the non‐bipartite graph:

(14) $$\left.\begin{array}{c} W=\sum_{i=1}^{\infty }w_{i}\delta_{\theta_{i}}\;\;W∼\text{CRM}\left(\rho ,\lambda \right),\\ W^{\prime }=\sum_{j=1}^{\infty }w_{j}^{\prime }\delta_{\theta_{j}^{\prime }}\;\;W^{\prime }∼\text{CRM}\left(\rho^{\prime },\lambda \right),\\ D=\sum_{i=1}^{\infty }\sum_{j=1}^{\infty }n_{ij}\delta_{\left(\theta_{i},\theta_{j}^{\prime }\right)}\;\;D|W,W^{\prime }∼\text{PP}\left(W\times W^{\prime }\right),\\ Z=\sum_{i=1}^{\infty }\sum_{j=1}^{\infty }\min\left(n_{ij},1\right)\delta_{\left(\theta_{i},\theta_{j}^{\prime }\right)}. \end{array}\right\}$$

Model (14) has been proposed by Caron (2012) in a slightly different formulation. In this paper, we recast this model within our general framework, enabling new theoretical and practical insights.

### 3.5 Interpretation of $\theta_{i}$

We can think of the positive, continuous valued node index $\theta_{i}$ as representing the time at which a *potential node* enters the network and has the opportunity to link with other existing nodes $\theta_{j}<\theta_{i}$. We use the terminology ‘potential node’ here to clarify that this node need not form any observed connections with other nodes existing before time $\theta_{i}$. We emphasize that an observed link between $\theta_{i}$ and some other node $\theta_{k}>\theta_{i}$ will eventually occur almost surely as time progresses. This could represent, for example, signing onto a social networking service before your friends do and only forming a link once they join. On the basis of our CRM specification, we have almost surely an infinite number of potential nodes as time goes to ∞. For infinite activity CRMs, we have almost surely an infinite set of potential nodes even at any finite time.

In Section 5, we examine properties of the network process across time, and we describe methods for simulating networks at any finite time. There, our focus is on the observed link process from this set of potential nodes. For example, sparsity is examined with respect to the set of nodes with degree at least 1, not with respect to the set of potential nodes. Since we need not think of $\theta_{i}$ as a time index, but rather just a general construct of our formulation, we also generically refer to $\theta_{i}$ as the node *location* in the remainder of the paper.

## 4 Related work

There has been extensive work over recent years on flexible Bayesian non‐parametric models for networks, allowing complex latent structures of unknown dimension to be uncovered from real world networks (Kemp *et al*., 2006; Miller *et al*., 2009; Lloyd *et al*., 2012; Palla *et al*., 2012; Herlau *et al*., 2014). However, as mentioned in the unifying overview of Orbanz and Roy (2015), these methods all fit in the Aldous–Hoover framework and as such produce dense graphs.

Norros and Reittu (2006) proposed a conditionally Poissonian multigraph process with similarities to be drawn to our multigraph process. In their formulation, each node has a given sociability parameter and the number of edges between two nodes *i* and *j* is drawn from a Poisson distribution with rate the product of the sociability parameters, normalized by the sum of the sociability parameters of all the nodes. The normalization makes this model similar to models based on rescaling of the graphon and, as such, does not define a projective model, as explained in Section 1. See van der Hofstad (2014) for a review of this model and Britton *et al*. (2006) for a similar model.

As pointed out by Jacobs and Clauset (2014) in their discussion of an earlier version of this paper, another related model is the degree‐corrected random‐graph model (Karrer and Newman, 2011), where edges of the multigraph are drawn from a Poisson distribution whose rate is the product of node‐specific sociability parameters and a parameter tuning the interaction between the latent communities to which these nodes belong. When the sociability parameters are assumed to be IID from some distribution, this model yields an exchangeable adjacency matrix and thus a dense graph.

Additionally, there are similarities to be drawn with the extensive literature on latent space modelling (e.g. Hoff *et al*. (2002), Penrose (2003) and Hoff (2009)). In such models, nodes are embedded in a low dimensional, continuous latent space and the probability of an edge is determined by a distance or similarity metric of the node‐specific latent factors. In our case, the continuous node index $\theta_{i}$ is of no importance in forming edge probabilities. It would, however, be possible to extend our approach to time‐ or location‐dependent connections by considering inhomogeneous CRMs.

Finally, as we shall detail in Section 5.5, our model admits a construction with connections to the configuration model (Bollobás, 1980; Newman, 2010), which is a popular model for generating simple graphs with a given degree sequence.

The connections with this broad set of past work place our proposed network model within the context of existing literature. Importantly, however, to the best of our knowledge this work represents the first fully generative and projective approach to sparse graph modelling (see Section 5), and with a notion of exchangeability that is essential for devising our scalable statistical estimation procedure, as shown in Section 7.

## 5 General properties and simulation

We provide general properties of our network model depending on the properties of the Lévy measure *ρ*.

### 5.1 Exchangeability under the Kallenberg framework

Proposition 1 (joint exchangeability of undirected graph measure). For any CRM *W*∼CRM(*ρ*,*λ*), the point process *Z* defined by equation (12), or equivalently by equation (6), is jointly exchangeable.

The proof is given in Appendix B. In the adjacency matrix representation, we think of exchangeability as invariance to node orderings. Here, we have invariance to the time of arrival of the nodes, thinking of $\theta_{i}$ as a time index.

We now reformulate our network process in the Kallenberg representation (5). Because of exchangeability, we know that such a representation exists. What we show here is that our CRM‐based formulation has an analytic and interpretable representation. In particular, the CRM *W* can be constructed from a two‐dimensional unit rate Poisson process on $R_{+}^{2}$ by using the inverse Lévy method (Khintchine, 1937; Ferguson and Klass, 1972). Let (*θ*_*i*_,*ϑ*_*i*_) be a unit rate Poisson process on $R_{+}^{2}$. Let $\overset{¯}{\rho }(x)$ be the *tail Lévy intensity*

(15) $$\overset{¯}{\rho }\left(x\right)=\int_{x}^{\infty }\rho \left(dw\right).$$

Then the CRM $W=\Sigma_{i}w_{i}\delta_{\theta_{i}}$ with Lévy measure *ρ*(d*w*)d*θ* can be constructed from the bidimensional point process by taking $w_{i}={\overset{¯}{\rho }}^{-1}\left(\vartheta_{i}\right)$. Note that the *inverse Lévy intensity* ${\overset{¯}{\rho }}^{-1}$ is a monotone function. It follows that our undirected graph model can be formulated under representation (5) by selecting any *α*_0_, *β*_0_=*γ*_0_=0, *g*=*g* ^′^=0, *h*=*h* ^′^=*l*=*l* ^′^=0 and

(16) $$f\left(\alpha_{0},\vartheta_{i},\vartheta_{j},\zeta_{\left\{i,j\right\}}\right)=\left\{\begin{array}{cc} 1 & \zeta_{\left\{i,j\right\}}⩽M\left(\vartheta_{i},\vartheta_{j}\right),\\ 0 & \text{otherwise} \end{array}\right.$$

where $M:R_{+}^{2}\to \left[0,1\right]$ is defined by

$$M\left(\vartheta_{i},\vartheta_{j}\right)=\left\{\begin{array}{cc} 1-\exp\{-2{\overset{¯}{\rho }}^{-1}\left(\vartheta_{i}\right){\overset{¯}{\rho }}^{-1}\left(\vartheta_{j}\right)\} & \text{if}\;\vartheta_{i}\neq \vartheta_{j},\\ 1-\exp\{-{\overset{¯}{\rho }}^{-1}{\left(\vartheta_{i}\right)}^{2}\} & \text{if}\;\vartheta_{i}=\vartheta_{j}. \end{array}\right.$$

**Figure 5:**
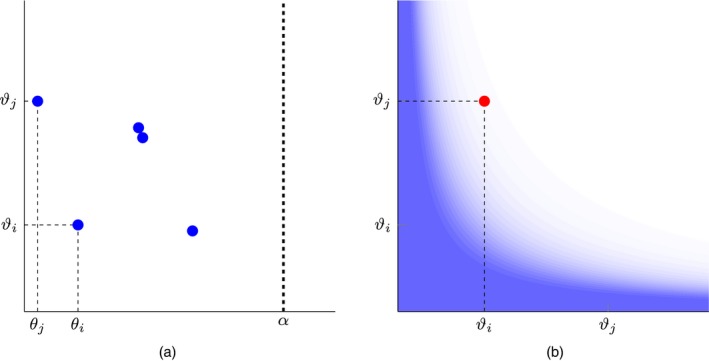
Illustration of the model construction based on the Kallenberg representation: (a) a unit rate Poisson process $\left(\theta_{i},\vartheta_{i}\right)$, i∈N, on $\left[0,\alpha \right]\times R_{+}$; (b) for each pair $\left\{i,j\right\}\in \tilde{N}{}^{2}$, set *z*_*ij*_=*z*_*ji*_=1 with probability *M*(*ϑ*_*i*_,*ϑ*_*j*_) (here, *M* is indicated by the blue shading (darker shading indicates higher value) for a stable process (GGP with *τ*=0); in this case there is an analytic expression for ${\overset{¯}{\rho }}^{-1}$ and therefore *M*)

In Section 6, we provide explicit forms for $\overset{¯}{\rho }$ depending on our choice of Lévy measure *ρ*. Expression (16) represents a direct analogue to that arising from the Aldous–Hoover framework. In particular, *M* here is akin to the graphon *ω* of expression (47) in Appendix A.1, and thus allows us to connect our CRM‐based formulation with the extensive literature on graphons. An illustration of the network construction from the Kallenberg representation, including the function *M*, is in Fig. 5. Note that, if we had started from the Kallenberg representation and selected an *f* (or *M*) arbitrarily, we would probably not have obtained a network model with the normalized CRM interpretation that enables both interpretability and analysis of network properties.

For the bipartite graph, Kallenberg's representation theorem for *separate* exchangeability (Kallenberg (1990) and Kallenberg (2005), theorem 9.23) can likewise be applied.

### 5.2 Interactions between groups

For any disjoint set of nodes $A,B\subset R_{+}$, *A*∩*B*=∅, the probability that there is at least one connection between a node in *A* and a node in *B* is given by

Pr{Z(A×B)>0|W}=1−exp{−2W(A)W(B)},

i.e. the probability of a between‐group edge depends on the sum of the sociabilities in each group, *W*(*A*) and *W*(*B*).

### 5.3 Graph restrictions

Let us consider the restriction of our process to the square [0,*α*]^2^. For finite activity CRMs, there will be a finite number of potential nodes (jumps) in the interval [0,*α*]. For infinite activity CRMs, we shall have an infinite number of potential nodes. We are interested in the properties of the process as *α* grows, where we can think of *α* as representing *time* and observing the process as new potential nodes and any resulting edges enter the network. We note that, in the limit of *α*→∞, the number of edges approaches ∞ since $W(R_{+})=\infty$ almost surely.

Let *D*_*α*_ and *Z*_*α*_ be the restrictions of *D* and *Z* respectively to the square [0,*α*]^2^. Then, (*D*_*α*_)_*α*⩾0_ and (*Z*_*α*_)_*α*⩾0_ are measure‐valued stochastic processes, indexed by *α*. We also denote by *W*_*α*_ and *λ*_*α*_ the corresponding CRM and Lebesgue measure on [0,*α*]. In what follows, our interests are in studying how the following quantities vary with *α*:

- $N_{\alpha }$, the number of nodes with degree at least one in the network, and
- $N_{\alpha }^{\left(e\right)}$, the number of edges in the undirected network.

We refer to $N_{\alpha }$ as the number of *observed nodes*. In our construction, recall that ${\left(N_{\alpha }\right)}_{\alpha ⩾0}$ and ${\left(N_{\alpha }^{\left(e\right)}\right)}_{\alpha ⩾0}$ are non‐decreasing, integer‐valued stochastic processes corresponding to the number of nodes with at least one connection in *Z*_*α*_ and the number of edges in *Z*_*α*_ respectively. Formally,

(17) $$N_{\alpha }=\text{card}\left(\left\{\theta_{i}\in \left[0,\alpha \right]|Z\left(\left\{\theta_{i}\right\}\times \left[0,\alpha \right]\right)>0\right\}\right),$$

(18) $$N_{\alpha }^{\left(e\right)}=Z\left[\left\{\left(x,y\right)\in R_{+}^{2}|0⩽x⩽y⩽\alpha \right\}\right].$$

**Figure 6:**
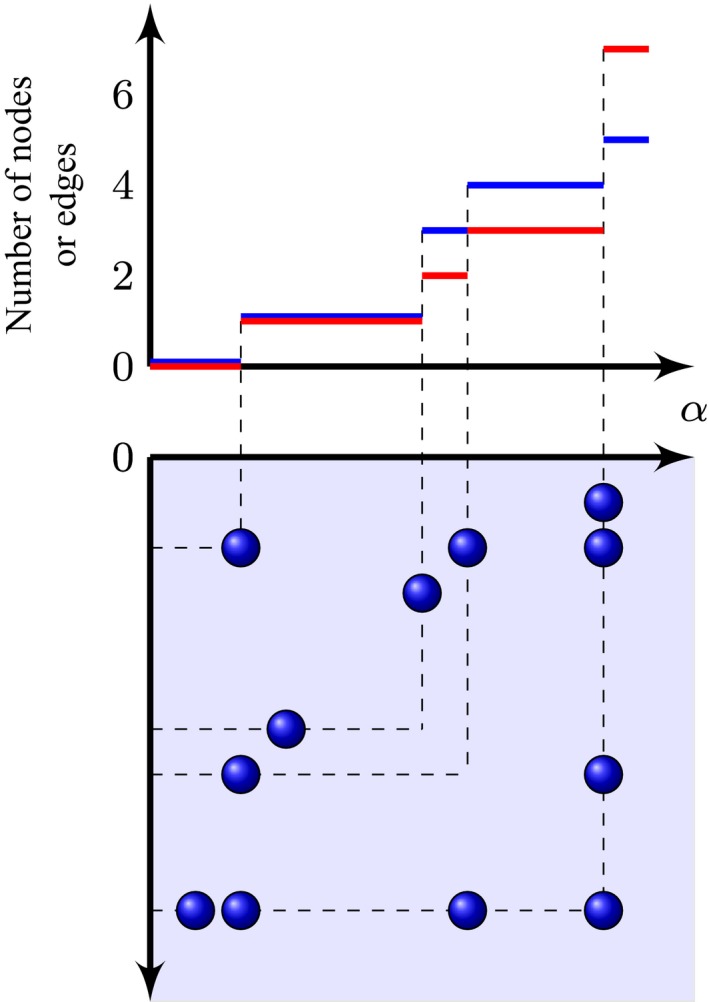
Example of point process *Z* and above it the associated integer‐valued stochastic processes for the number of observed nodes ${\left(N_{\alpha }\right)}_{\alpha ⩾0}$ (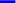) and edges ${\left(N_{\alpha }^{\left(e\right)}\right)}_{\alpha ⩾0}$ (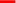)

The two processes have the same jump times, which correspond to the addition of one or more new nodes with at least one connection in the graph. An example of these processes is represented in Fig. 6. In later sections we use $Z_{\alpha }^{*}=Z_{\alpha }\left({\left[0,\alpha \right]}^{2}\right)$ to denote the total mass on [0,*α*]^2^, and similarly for $D_{\alpha }^{*}$ and $W_{\alpha }^{*}$.

### 5.4 Sparsity

In this section we state the sparsity properties of our graph model, which relate to the properties of the Lévy measure *ρ*. In particular, we are interested in the relative asymptotic behaviour of the number of edges $N_{\alpha }^{\left(e\right)}$ with respect to the number of observed nodes $N_{\alpha }$ as *α*→∞. Henceforth, we consider $\int_{0}^{\infty }\rho \left(dw\right)>0$, since the case of $\int_{0}^{\infty }\rho \left(dw\right)=0$ trivially gives $N_{\alpha }^{\left(e\right)}=N_{\alpha }=0$ almost surely.

In theorem 2 we characterize the sparsity of the graph with respect to the properties of its Lévy measure: graphs obtained from infinite activity CRMs are sparse, whereas graphs obtained from finite activity CRMs are dense. The rate of growth can be further specified when *ρ* is a *regularly varying* Lévy measure (Feller, 1971; Karlin, 1967; Gnedin *et al*., 2006, 2007), as defined in Appendix A.2. We follow the notation of Janson (2011) for probability asymptotics (see Appendix C.1 for details).

Theorem 2 Consider a point process *Z* representing an undirected graph. Let $N_{\alpha }^{\left(e\right)}$ be the number of edges and $N_{\alpha }$ be the number of observed nodes in the point process restriction *Z*_*α*_ (see equations (17) and (18)). Assume that the defining Lévy measure is such that $\int_{0}^{\infty }w\rho \left(dw\right)<\infty$. If the CRM *W* is *finite activity*, i.e.

$$\int_{0}^{\infty }\rho \left(dw\right)<\infty ,$$

then the number of edges scales quadratically with the number of observed nodes

(19) $$N_{\alpha }^{\left(e\right)}=\Theta \left(N_{\alpha }^{2}\right)$$

almost surely as *α*→∞, implying that the graph is *dense*.

If the CRM is *infinite activity*, i.e.

$$\int_{0}^{\infty }\rho \left(dw\right)=\infty ,$$

then the number of edges scales subquadratically with the number of observed nodes

(20) $$N_{\alpha }^{\left(e\right)}=o\left(N_{\alpha }^{2}\right)$$

almost surely as *α*→∞, implying that the graph is *sparse*.

Furthermore, if the Lévy measure *ρ* is *regularly varying* (see definition 1 in Appendix A.2), with exponent *σ* ∈ (0,1) and slowly varying function *l* satisfying $\lim\inf_{t\to \infty }l\left(t\right)>0$, then

(21) $$N_{\alpha }^{\left(e\right)}=O\left(N_{\alpha }^{2/\left(1+\sigma \right)}\right)\;\;\text{almost surely as}\;\alpha \to \infty .$$

Theorem 2 is a direct consequence of two theorems that we state now and prove in Appendix C. The first theorem states that the number of edges grows quadratically with *α*, whereas the second states that the number of nodes scales superlinearly with *α* for infinite activity CRMs, and linearly otherwise.

Theorem 3 Consider the point process *Z*. If $\int_{0}^{\infty }w\rho \left(dw\right)<\infty$, then the number of edges in *Z*_*α*_ grows quadratically with *α*:

(22) $$N_{\alpha }^{\left(e\right)}=\Theta \left(\alpha^{2}\right)$$

almost surely. Otherwise, $N_{\alpha }^{\left(e\right)}=\Omega \left(\alpha^{2}\right)$ almost surely.

Theorem 4 Consider the point process *Z*. Then

(23) $$N_{\alpha }=\left\{\begin{array}{cc} \Theta (\alpha ) & \text{if}\;W\;\text{is a finite activity CRM},\\ \omega (\alpha ) & \text{if}\;W\;\text{is an infinite activity CRM} \end{array}\right.$$

almost surely as *α*→∞. In words, the number of nodes with degree at least 1 in *Z*_*α*_ scales linearly with *α* for finite activity CRMs and superlinearly with *α* for infinite activity CRMs. Furthermore, for a regularly varying Lévy measure with slowly varying function *l* such that ${\lim\inf}_{t\to \infty }l\left(t\right)>0$, we have

(24) $$N_{\alpha }=\Omega \left(\alpha^{\sigma +1}\right)\;\;\text{almost surely as}\;\alpha \to \;\infty .$$

We finally give the expressions of the expectations for the number of edges and nodes in the model. The proof is given in Appendix C.4. (Equations (26) and (27) could alternatively be derived as particular cases of the results in Veitch and Roy (2015).)

Theorem 5 The expected number of edges $D_{\alpha }^{*}$ in the multigraph, edges $N_{\alpha }^{\left(e\right)}$ in the undirected graph and observed nodes $N_{\alpha }$ are given as follows:

(25) $$E\left[D_{\alpha }^{*}\right]=\alpha^{2}{\left\{\int_{0}^{\infty }w\rho \left(dw\right)\right\}}^{2}+\alpha \int_{0}^{\infty }w^{2}\rho \left(dw\right),$$

(26) $$E\left[N_{\alpha }^{\left(e\right)}\right]=\frac{1}{2}\alpha^{2}\int_{0}^{\infty }\psi \left(2w\right)\rho \left(dw\right)+\alpha \int_{0}^{\infty }\left\{1-\exp\left(-w^{2}\right)\right\}\rho \left(dw\right),$$

(27) $$E\left[N_{\alpha }\right]=\alpha \int_{0}^{\infty }\left[1-\exp\left\{-w^{2}-\alpha \psi \left(2w\right)\right\}\right]\rho \left(dw\right),$$

where $\psi \left(t\right)=\int_{0}^{\infty }\left\{1-\exp\left(-wt\right)\right\}\rho \left(dw\right)$ is the Laplace exponent. Additionally, if *ρ* is a regularly varying Lévy measure with exponent *σ* ∈ [0,1) and slowly varying function *l*, and $\int_{0}^{\infty }w\rho \left(dw\right)<\infty$ then

(28) $$E\left[N_{\alpha }\right]\overset{\alpha ↑\infty }{∼}\alpha^{1+\sigma }l\left(\alpha \right)\Gamma \left(1-\sigma \right){\left\{2\int_{0}^{\infty }w\rho \left(dw\right)\right\}}^{\sigma }.$$

### 5.5 Simulation

#### 5.5.1 Direct simulation of graph restrictions

By definition, the directed multigraph restriction *D*_*α*_ is drawn from a Poisson process with finite mean measure *W*_*α*_×*W*_*α*_, where *W*_*α*_∼CRM(*ρ*,*λ*_*α*_). Leveraging standard properties of the CRM and Poisson process, we can first simulate the total number of directed edges $D_{\alpha }^{*}$ based on the total mass $W_{\alpha }^{*}$:

(29) $$D_{\alpha }^{*}|W_{\alpha }^{*}∼\text{Poisson}\left(W_{\alpha }^{*\;2}\right).$$

For $k=1,\ldots ,D_{\alpha }^{*}$ a particular edge is drawn by sampling a pair of nodes

(30) $$U_{kj}|W_{\alpha }\overset{\mathrm{IID}}{∼}\frac{W_{\alpha }}{W_{\alpha }^{*}}j=1,2,$$

where $W_{\alpha }/W_{\alpha }^{*}$ is called a *normalized CRM*. We form directed edges (*U*_*k*1_,*U*_*k*2_), resulting in

(31) $$D_{\alpha }=\sum_{k=1}^{D_{\alpha }^{*}}\delta_{\left(U_{k1},U_{k2}\right)}.$$

Because of the discreteness of *W*_*α*_, there will be ties between the (*U*_*k*1_,*U*_*k*2_), and the number of such ties corresponds to the multiplicity of that edge. In particular, a total of $2D_{\alpha }^{*}$ nodes *U*_*kj*_ are drawn but result in some $N_{\alpha }⩽2D_{\alpha }^{*}$ distinct values. We overload the notation $N_{\alpha }$ here because this quantity also corresponds to the number of nodes with degree at least 1 in the resulting undirected network. Recall that the undirected network construction simply forms an undirected edge between a set of nodes if there is at least one directed edge between them. If we consider unordered pairs {*U*_*k*1_,*U*_*k*2_}, the number of such unique pairs takes a number $N_{\alpha }^{\left(e\right)}⩽D_{\alpha }^{*}$ of distinct values, where $N_{\alpha }^{\left(e\right)}$ corresponds to the number of edges in the undirected network.

The construction above enables us to re‐express our Cox process model in terms of normalized CRMs (Regazzini *et al*., 2003). This is very attractive both practically and theoretically. As we show in Section 6 for special cases of CRMs, one can use the results surrounding normalized CRMs to derive an exact simulation technique for our directed and undirected graphs.

Remark 1 The construction above enables us to draw connections with the configuration model (Bollobás, 1980; Newman, 2010), which proceeds as follows. First, the degree *k*_*i*_ of each node *i*=1,…,*n* is specified such that the sum of *k*_*i*_ is an odd number. Each node *i* is given a total of *k*_*i*_ stubs, or *demiedges*. Then, we repeatedly choose pairs of stubs uniformly at random, without replacement, and connect the selected pairs to form an edge. The simple graph is obtained either by discarding the multiple edges and self‐loops (an *erased* configuration model), or by repeating the above sampling until obtaining a simple graph. In our case, we have an infinite set of (potential) nodes and do not prespecify the node degrees. Furthermore, each node in the pair (*U*_*k*1_,*U*_*k*2_) is drawn from a normalized CRM rather than the pair being selected uniformly at random. However, at a high level, there is a similar flavour to our construction.

#### 5.5.2 Urn‐based simulation of graph restrictions

We now describe an urn formulation that allows us to obtain a finite dimensional generative process. Recall that, in practice, we cannot sample *W*_*α*_∼CRM(*ρ*,*λ*_*α*_) if the CRM is infinite activity since there will be an infinite number of jumps.

Let $\left(U_{1}^{\prime },\ldots ,U_{2D_{\alpha }^{*}}^{\prime }\right)=\left(U_{11},U_{12},\ldots ,U_{D_{\alpha }^{*}1},U_{D_{\alpha }^{*}2}\right)$. For some classes of Lévy measure *ρ*, it is possible to integrate out the normalized CRM $\mu_{\alpha }=W_{\alpha }/W_{\alpha }^{*}$ in expression (30) and to derive the conditional distribution of $U_{n+1}^{\prime }$ given $\left(W_{\alpha }^{*},U_{1}^{\prime },\ldots ,U_{n}^{\prime }\right)$. We first recall some background on random partitions. As *μ*_*α*_ is discrete with probability 1, variables $U_{1}^{\prime },\ldots ,U_{n}^{\prime }$ take *k*⩽*n* distinct values ${\tilde{\theta }}_{j}$, with multiplicities $1⩽{\tilde{m}}_{j}⩽n$. The distribution on the underlying partition is usually defined in terms of an exchangeable partition probability function (EPPF) (Pitman, 1995) $\Pi_{n}^{\left(k\right)}\left({\tilde{m}}_{1},\ldots ,{\tilde{m}}_{k}|W_{\alpha }^{*}\right)$ which is symmetric in its arguments. The predictive distribution of $U_{n+1}^{\prime }$ given $\left(W_{\alpha }^{*},U_{1}^{\prime },\ldots ,U_{n}^{\prime }\right)$ is then given in terms of the EPPF:

(32) $$\begin{array}{cc} U_{n+1}^{\prime }|\left(W_{\alpha }^{*},U_{1}^{\prime },\ldots ,U_{n}^{\prime }\right) & ∼\frac{\Pi_{n+1}^{\left(k+1\right)}\left({\tilde{m}}_{1},\ldots ,{\tilde{m}}_{k},1|W_{\alpha }^{*}\right)}{\Pi_{n}^{\left(k\right)}\left({\tilde{m}}_{1},\ldots ,{\tilde{m}}_{k}|W_{\alpha }^{*}\right)}\frac{1}{\alpha }\lambda_{\alpha }\\ & \;+\sum_{j=1}^{k}\frac{\Pi_{n+1}^{\left(k\right)}\left({\tilde{m}}_{1},\ldots ,{\tilde{m}}_{j}+1,\ldots ,{\tilde{m}}_{k}|W_{\alpha }^{*}\right)}{\Pi_{n}^{\left(k\right)}\left({\tilde{m}}_{1},\ldots ,{\tilde{m}}_{k}|W_{\alpha }^{*}\right)}\delta_{{\tilde{\theta }}_{j}}. \end{array}$$

Using this urn representation, we can rewrite our generative process as

$$\begin{array}{cc} & W_{\alpha }^{*}∼P_{W_{\alpha }^{*}},\\ & D_{\alpha }^{*}|W_{\alpha }^{*}∼\text{Poisson}\left(W_{\alpha }^{*\;2}\right),\\ & {\left(U_{kj}\right)}_{k=1,\ldots ,D_{\alpha }^{*};j=1,2}|W_{\alpha }^{*}∼\text{urn process}\;\left(32\right), \end{array}$$

(33) $$\begin{array}{cc} & D_{\alpha }=\sum_{k=1}^{D_{\alpha }^{*}}\delta_{\left(U_{k1},U_{k2}\right)}, \end{array}$$

where $P_{W_{\alpha }^{*}}$ is the distribution of the CRM total mass $W_{\alpha }^{*}$. Representation (33) can be used to sample *exactly* from our graph model, assuming that we can sample from $P_{W_{\alpha }^{*}}$ and evaluate the EPPF. In Section 6 we show that this is indeed possible for specific CRMs of interest.

#### 5.5.3 Approximate simulation of graph restrictions

If we cannot sample from $P_{W_{\alpha }^{*}}$ in expression (33) and evaluate the EPPF in expression (32), we resort to approximate simulation methods. In particular, we harness the directed multigraph representation and approximate the draw of *W*_*α*_. For our undirected graphs, we simply transform the (approximate) draw of a directed multigraph as described in Section 3.3.

One approach to approximate simulation of *W*_*α*_, which is possible for some Lévy measures *ρ*, is to resort to adaptive thinning (Lewis and Shedler, 1979; Ogata, 1981; Favaro and Teh, 2013). A related alternative approximate approach, but applicable to any Lévy measure *ρ* satisfying condition (9), is the inverse Lévy method. This method first defines a threshold *ɛ* and then samples the weights $\Omega =\{w_{i}|w_{i}>\varepsilon \}$ by using a Poisson measure on [*ɛ*,∞]. One then simulates *D*_*α*_ using these truncated weights Ω.

A naive application of this truncated method that considers sampling directed or undirected edges as in expression (12) or expression (6) respectively can prove computationally problematic since a large number of possible edges must be considered (one Poisson or Bernoulli draw for each $\left(\theta_{i},\theta_{j}\right)$ pair for the directed or undirected case). Instead, we can harness the Cox process representation and resulting sampling procedure of expression (29)–(30) to sample first the total number of directed edges and then their specific instantiations. More specifically, to simulate approximately a point process on [0,*α*]^2^, we use the inverse Lévy method to sample

(34) $$\Pi_{\alpha ,\varepsilon }=\left\{\left(w,\theta \right)\in \Pi ,0<\theta ⩽\alpha ,w>\varepsilon \right\}.$$

Let $W_{\alpha ,\varepsilon }=\Sigma_{i=1}^{K}w_{i}\delta_{\theta_{i}}$ be the associated truncated CRM and $W_{\alpha ,\varepsilon }^{*}=W_{\alpha ,\varepsilon }\left(\left[0,\alpha \right]\right)$ its total mass. We then sample $D_{\alpha ,\varepsilon }^{*}$ and *U*_*kj*_ as in expression (29)–(30), and set $D_{\alpha ,\varepsilon }=\Sigma_{k=1}^{D_{\alpha ,\varepsilon }^{*}}\delta_{\left(U_{k1},U_{k2}\right)}$. The undirected graph measure *Z*_*α*,*ɛ*_ is set to the manipulation of *D*_*α*,*ɛ*_ as in expression (12).

## 6 Special cases

In this section, we examine the properties of various models and their link to classical random‐graph models depending on the Lévy measure *ρ*. We show that, in the GGP case, the resulting graph can be either dense or sparse, with the sparsity tuned by a single hyperparameter. Furthermore, exact simulation is possible via expression (33). We focus on the undirected graph case, but similar results can be obtained for directed multigraphs and bipartite graphs.

### 6.1 Poisson process

Consider a Poisson process with fixed increments $w_{0}>0$:

$$\rho \left(dw\right)=\delta_{w_{0}}\left(dw\right).$$

This measure *ρ* defines a finite activity CRM. Recalling the definition $\overset{¯}{\rho }\left(x\right)=\int_{x}^{\infty }\rho \left(dw\right)$, in this case, we have

$$\overset{¯}{\rho }\left(x\right)=\left\{\begin{array}{cc} 1 & \text{if}\;x<w_{0},\\ 0 & \text{otherwise}. \end{array}\right.$$

Ignoring self‐edges, the graph construction can be described as follows. To sample *W*_*α*_∼CRM(*ρ*,*λ*_*α*_), we generate *n*∼Poisson(*α*) and then sample $\theta_{i}∼\text{Unif}\left(\left[0,\alpha \right]\right)$ for *i*=1,…,*n*. We then sample edges according to expression (6): for 0<*i*<*j*<*n*, set *z*_*ij*_=*z*_*ji*_=1 with probability $1-\exp(-2w_{0}^{2})$ and 0 otherwise. The model is therefore equivalent to the Erdoós–Rényi random‐graph model *G*(*n*,*p*) with *n*∼Poisson(*α*) and $p=1-\exp(-2w_{0}^{2})$. Therefore, this choice of *ρ* leads to a *dense* graph, as our theory suggests, where the number of edges grows quadratically with the number of nodes *n*.

### 6.2 Compound Poisson process

A compound Poisson process is a process where

ρ(dw)=h(w)dw

and $h:R_{+}\to R_{+}$ is such that $\int_{0}^{\infty }h\left(w\right)dw=1$ and defines a finite activity CRM. In this case, we have $\overset{¯}{\rho }(x)=1-H(x)$ where *H* is the distribution function that is associated with *h*. Here, we arrive at a framework that is similar to the standard graphon. Leveraging the Kallenberg representation (16), we first sample *n*∼Poisson(*α*). Then, for *i*=1,…,*n* we set *z*_*ij*_=*z*_*ji*_=1 with probability *M*(*U*_*i*_,*U*_*j*_) where *U*_*i*_ are uniform [0,1] variables and *M* is defined by

$$M\left(U_{i},U_{j}\right)=1-\exp\left\{-2H^{-1}\left(U_{i}\right)H^{-1}\left(U_{j}\right)\right\}.$$

This representation is the same as with the Aldous–Hoover theorem, except that the number of nodes is random and follows a Poisson distribution. As such, the resulting random graph is either trivially empty or *dense*, again agreeing with our theory.

### 6.3 Generalized gamma process

The GGP (Hougaard, 1986; Aalen, 1992; Lee and Whitmore, 1993; Brix, 1999) is a flexible two‐parameter CRM with interpretable parameters and remarkable conjugacy properties (James, 2002; Lijoi and Prünster, 2003; Lijoi *et al*., 2007; Caron *et al*., 2014). The process is also known as the Hougaard process (Hougaard, 1986) when *λ* is the Lebesgue measure, as in this paper, but we shall use the more standard term GGP in the rest of this paper. The Lévy measure of the GGP is given by

(35) $$\rho \left(dw\right)=\frac{1}{\Gamma (1-\sigma )}w^{-1-\sigma }\exp\left(-\tau w\right)dw,$$

where the two parameters (*σ*,*τ*) satisfy

(36) $$\left(\sigma ,\tau \right)\in \left(\right.-\infty ,0]\times \left(0,\infty \right)\;\text{or}\;\left(\sigma ,\tau \right)\in \left(0,1\right)\times \left[\right.0,\infty ).$$

The GGP has different properties if *σ*⩾0 or *σ*<0. When *σ*<0, the GGP is a finite activity CRM (i.e. a compound Poisson process); more precisely, the number of jumps in [0,*α*] is finite with probability 1 and drawn from a Poisson distribution with rate −(*α*/*σ*)*τ* ^*σ*^ whereas the jumps *w*_*i*_ are IID gamma(−*σ*,*τ*).

When *σ*⩾0, the GGP has an infinite number of jumps over any interval [*s*,*t*]. It includes as special cases the gamma process (*σ*=0, *τ*>0), the stable process (*σ* ∈ (0,1), *τ*=0) and the inverse Gaussian process ($\sigma =\frac{1}{2}$, *τ*>0).

**Figure 7:**
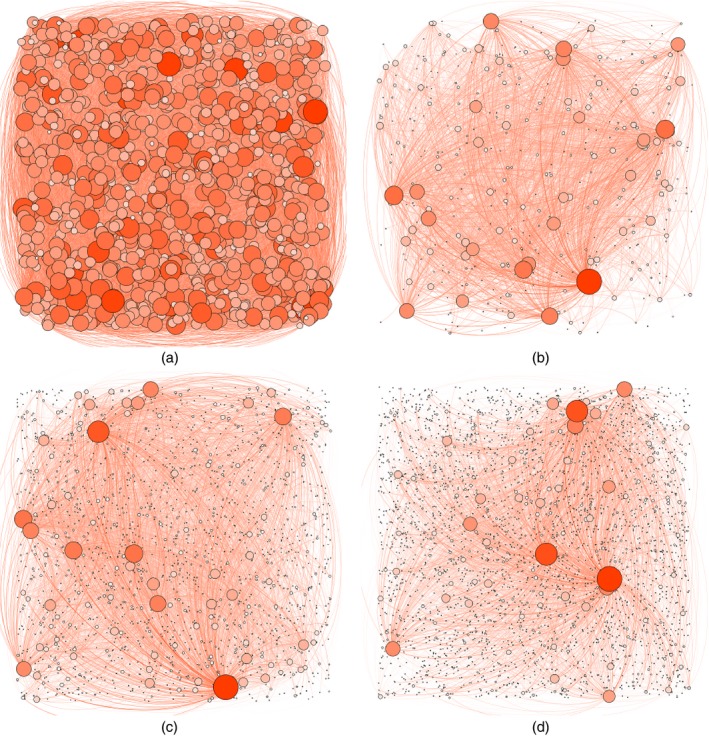
Sample graphs: (a) Erdoós–Rényi graph *G*(*n*,*p*) with *n*=1000 and *p*=0.05, and GGP graphs GGP(*α*,*τ*,*σ*) with (b)–(d) *α*=100, *τ*=2 and (b) *σ*=0, (c) *σ*=0.5 and (d) *σ*=0.8 (the size of a node is proportional to its degree; the graphs were generated with the software Gephi (Bastian *et al*., 2009))

The tail Lévy intensity of the GGP is given by

$$\overset{¯}{\rho }\left(x\right)=\int_{x}^{\infty }\frac{1}{\Gamma (1-\sigma )}w^{-1-\sigma }\exp\left(-\tau w\right)dw=\left\{\begin{array}{cc} \frac{\tau^{\sigma }\Gamma \left(-\sigma ,\tau x\right)}{\Gamma (1-\sigma )} & \text{if}\;\tau >0,\\ \frac{x^{-\sigma }}{\Gamma (1-\sigma )\sigma } & \text{if}\;\tau =0, \end{array}\right.$$

where Γ(*a*,*x*) is the incomplete gamma function. Example realizations of the process for various values of *σ*⩾0 are displayed in Fig. 7 alongside a realization of an Erdös–Rényi graph.

#### 6.3.1 Exact sampling via an urn approach

In the case *σ*>0, $W_{\alpha }^{*}$ is an exponentially tilted stable random variable, for which exact samplers exist (Devroye, 2009). As shown by Pitman (2003) (see also Lijoi *et al*. (2008)), the EPPF conditional on the total mass $W_{\alpha }^{*}=t$ depends only on the parameter *σ* (and not *τ* and *α*) and is given by

(37) $$\Pi_{k}^{\left(n\right)}\left({\tilde{m}}_{1},\ldots ,{\tilde{m}}_{k}|t\right)=\frac{\sigma^{k}t^{-n}}{\Gamma \left(n-k\sigma \right)g_{\sigma }\left(t\right)}\int_{0}^{t}s^{n-k\sigma -1}g_{\sigma }\left(t-s\right)ds\prod_{i=1}^{k}\frac{\Gamma ({\tilde{m}}_{i}-\sigma )}{\Gamma (1-\sigma )},$$

where *g*_*σ*_ is the probability density function of the positive stable distribution. Plugging the EPPF (37) into expression (32) yields the urn process for sampling in the GGP case. In particular, we can use the generative process (33) to sample exactly from the model.

In the special case of the gamma process (*σ*=0), $W_{\alpha }^{*}$ is a gamma(*α*,*τ*) random variable and the resulting urn process is given by (Blackwell and MacQueen, 1973; Pitman, 1996)

(38) $$U_{n+1}^{\prime }|\left(W_{\alpha }^{*},U_{1}^{\prime },\ldots ,U_{n}^{\prime }\right)∼\frac{\alpha }{\alpha +n}\frac{\lambda_{\alpha }}{\alpha }+\sum_{j=1}^{k}\frac{{\tilde{m}}_{j}}{\alpha +n}\delta_{{\tilde{\theta }}_{j}}.$$

When *σ*<0, the GGP is a compound Poisson process and can thus be sampled exactly.

#### 6.3.2 Sparsity

Appealing to theorem 2, we use the following facts about the GGP to characterize the sparsity properties of this special case.

- For *σ*<0, the CRM is *finite activity* with $\int_{0}^{\infty }w\rho \left(dw\right)<\infty$; thus theorem 2 implies that the graph is *dense*.
- When *σ*⩾0 the CRM is *infinite activity*; moreover, for *τ*>0, $\int_{0}^{\infty }w\rho \left(dw\right)<\infty$, and thus theorem 2 implies that the graph is *sparse*.
- For *σ*>0, the tail Lévy intensity has the asymptotic behaviour
  $$\overset{¯}{\rho }\left(x\right)\overset{x↓0}{∼}\frac{1}{\sigma \Gamma (1-\sigma )}x^{-\sigma }$$
  and, as such, is *regularly varying* with exponent *σ* and constant slowly varying function.

We thus conclude that

(39) $$N_{\alpha }^{\left(e\right)}=\left\{\begin{array}{cc} \Theta (N_{\alpha }^{2}) & \text{if}\;\sigma <0,\\ o(N_{\alpha }^{2}) & \text{if}\;\sigma =0,\tau >0,\\ O(N_{\alpha }^{2/(1+\sigma )}) & \text{if}\;\sigma \in (0,1),\tau >0, \end{array}\right.$$

almost surely as *α*→∞, i.e. the GGP parameter *σ* tunes the sparsity of the graph. The underlying graph is sparse if *σ*⩾0 and dense otherwise.

Remark 2 The proof technique of theorem 2 requires $\int_{0}^{\infty }w\rho \left(dw\right)<\infty$ and thus excludes the stable process (*τ*=0,*σ* ∈ (0,1)), although we conjecture that the graph is also sparse in that case.

Additionally, applying theorem 5, we obtain

$$E\left[N_{\alpha }\right]\underset{\alpha ↑\infty }{∼}\left\{\begin{array}{cc} \alpha \frac{-\tau^{\sigma }}{\sigma } & \text{if}\;\sigma <0,\\ \alpha \log(\alpha ) & \text{if}\;\sigma =0,\\ \alpha^{1+\sigma }\frac{2^{\sigma }\tau^{\sigma (\sigma -1)}}{\sigma } & \text{if}\;\sigma >0,\tau >0. \end{array}\right.$$

#### 6.3.3 Empirical analysis of graph properties

**Figure 8:**
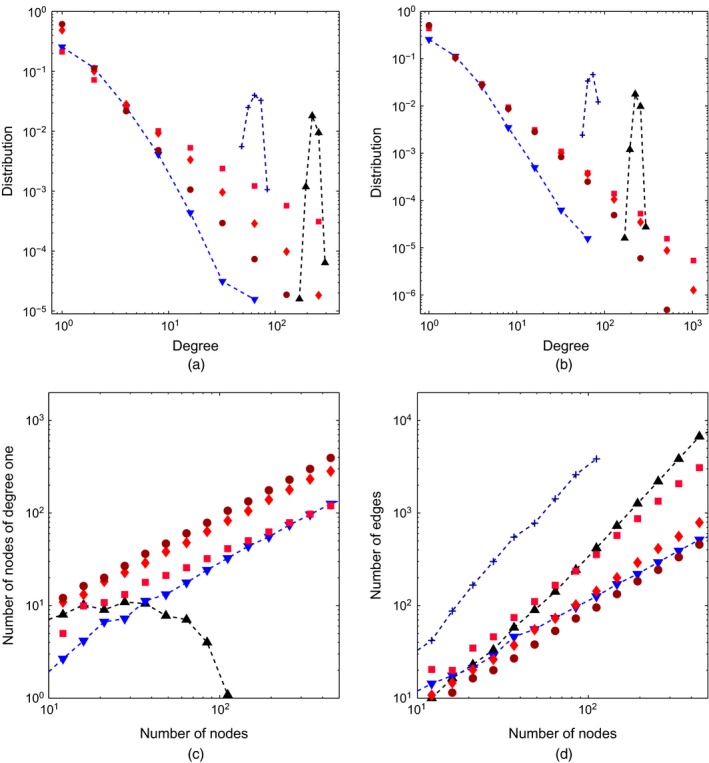
Examination of the GGP undirected network properties (averaging over graphs with various *α*) in comparison with an Erdoós–Rényi *G*(*n*,*p*) model with *p*=0.05 (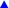), the preferential attachment model of Barabási and Albert (1999) (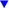) and the non‐parametric formulation of Lloyd *et al*. (2012) (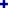): (a) degree distribution on a log–log‐scale for (a) various values of *σ* (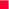, *σ*=0.2; 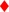, *σ*=0.5; 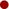, *σ*=0.8) (*τ*=10^−2^) and (b) various values of *τ* (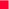, *τ*=10^−1^; 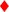, *τ*=1; 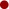, *τ*=5) (*σ*=0.5) for the GGP; (c) number of nodes with degree 1 *versus* number of nodes on a log–log‐scale (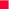, *σ*=0.2; 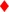, *σ*=0.5; 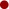, *σ*=0.8) (note that the Lloyd method leads to dense graphs such that no node has only degree 1); (d) number of edges *versus* number of nodes (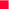, *σ*=0.2; 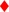, *σ*=0.5; 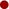, *σ*=0.8) (here we note growth at a rate *o*(*n* ^2^) for our GGP graph models, and *Θ*(*n* ^2^) for the Erdoós‐Rényi and Lloyd models (dense graphs))

For the GGP‐based formulation, we provide an empirical analysis of our network properties in Fig. 8 by simulating undirected graphs by using the approach that was described in Section 5.5 for various values of *σ* and *τ*. We compare with an Erdoós–Rényi random graph, preferential attachment (Barabási and Albert, 1999) and the Bayesian non‐parametric network model of Lloyd *et al*. (2012). The particular features that we explore are as follows.

- *Degree distribution*: Fig. 8(a) suggests empirically that the model can exhibit power law behaviour providing a heavy‐tailed degree distribution. As shown in Fig. 8(b), the model can also handle an exponential cut‐off in the tails of the degree distribution, which is an attractive property (Clauset *et al*., 2009; Olhede and Wolfe, 2012).
- *Number of degree 1 nodes*: Fig. 8(c) examines the fraction of degree 1 nodes *versus* the number of nodes.
- *Sparsity*: Fig. 8(d) plots number of edges *versus* number of nodes. The larger *σ*, the sparser the graph is. In particular, for the GGP random‐graph model, we have network growth at a rate *O*(*n* ^*a*^) for 1<*a*<2 whereas the Erdös–Rényi (dense) graph grows as *Θ*(*n* ^2^).

#### 6.3.4 Interpretation of hyperparameters

On the basis of the properties derived and illustrated empirically in this section, we see that our hyperparameters have the following interpretations.

- ***σ***—from Figs 8(a) and 8(d), *σ* relates to the slope of the degree distribution in its power law regime and the overall network sparsity. Increasing *σ* leads to higher power law exponent and sparser networks.
- ***α***—from theorem 5, *α* provides an overall scale that affects the number of nodes and directed interactions, with larger *α* leading to larger networks.
- ***τ***—from Fig. 8(b), *τ* determines the exponential decay of the tails of the degree distribution, with *τ* small looking like pure power law. This is intuitive from the form of *ρ*(d*w*) in equation (35), where we see that *τ* affects large weights more than small weights.

## 7 Posterior characterization and inference

In this section, we consider the posterior characterization and MCMC inference of parameters and hyperparameters in our statistical network models.

Assume that we have observed a set of undirected connections ${\left(z_{ij}\right)}_{1⩽i,j⩽N_{\alpha }}$ or directed connections ${\left(n_{ij}\right)}_{1⩽i,j⩽N_{\alpha }}$ where $N_{\alpha }$ is the observed number of nodes with at least one connection. Without loss of generality, we assume that the locations of these nodes $0<\theta_{1}<\ldots <\theta_{N_{\alpha }}<\alpha$ are ordered, and we write $w_{i}=W\left(\left\{\theta_{i}\right\}\right)$ as their associated sociability parameters. For simplicity, we are overloading notation here with the *unordered* nodes in $W=\Sigma_{i}w_{i}\delta_{\theta_{i}}$ of equation (7).

We aim to infer the sociability parameters *w*_*i*_, $i=1,\ldots ,N_{\alpha }$, for each of the observed nodes. We also aim to infer the sociability parameters of the nodes with no connections (the difference between the set of potential nodes and those with observed interactions). We refer to these as *unobserved nodes*. Under our framework, the number of such nodes is either finite but unknown or infinite. The observed connections, however, provide information about only the sum of their sociabilities, denoted w_*_. The node locations $\theta_{i}$ of both observed and unobserved nodes are also not likelihood identifiable and are thus ignored. We additionally aim to estimate *α* and the hyperparameters of the Lévy intensity *ρ* of the CRM; we write *ϕ* for the set of hyperparameters. We therefore aim to approximate the posterior $p\{w_{1},\ldots ,w_{N_{\alpha }},w_{*},\phi |{\left(z_{ij}\right)}_{1<i,j<N_{\alpha }}\}$ for an observed undirected graph and $p\{w_{1},\ldots ,w_{N_{\alpha }},w_{*},\phi |{\left(n_{ij}\right)}_{1<i,j<N_{\alpha }}\}$ for an observed directed graph. (Formally, this density is with respect to a product measure that has a Dirac mass at 0 for w_*_, as detailed in Appendix F.)

### 7.1 Directed multigraph posterior

In theorem 6, we characterize the posterior in the directed multigraph case. This plays a key role in the undirected case that is explored in Section 7.2 as well.

Theorem 6 For $N_{\alpha }⩾1$, let $\theta_{1}<\ldots <\theta_{N_{\alpha }}$ be the set of support points of the measure *D*_*α*_ such that $D_{\alpha }=\Sigma_{1⩽i,j⩽N_{\alpha }}n_{ij}\delta_{\left(\theta_{i},\theta_{j}\right)}$. Let $w_{i}=W_{\alpha }\left(\left\{\theta_{i}\right\}\right)$ and $w_{*}=W_{\alpha }^{*}-\Sigma_{i=1}^{N_{\alpha }}w_{i}$. We have

(40) $$\begin{array}{cc} & P\{{\left(w_{i}\in dw_{i}\right)}_{1⩽i⩽N_{\alpha }},w_{*}\in dw_{*}|{\left(n_{ij}\right)}_{1⩽i,j⩽N_{\alpha }},\phi \}\\ & \propto \exp\left\{-{\left(\sum_{i=1}^{N_{\alpha }}w_{i}+w_{*}\right)}^{2}\right\}\left\{\prod_{i=1}^{N_{\alpha }}w_{i}^{m_{i}}\rho \left(dw_{i}\right)\right\}G_{\alpha }^{*}\left(dw_{*}\right) \end{array}$$

where $m_{i}=\Sigma_{j=1}^{N_{\alpha }}\left(n_{ij}+n_{ji}\right)>0$ for $i=1,\ldots ,N_{\alpha }$ are the node degrees of the multigraph and $G_{\alpha }^{*}$ is the probability distribution of the random variable $W_{\alpha }^{*}$, with Laplace transform

(41) $$E[\text{exp}\left(-tW_{\alpha }^{*}\right)]=\exp\left\{-\alpha \psi (t)\right\}.$$

Additionally, conditionally on observing an empty graph, i.e. $N_{\alpha }=0$, we have

(42) $$P\left(w_{*}\in dw_{*}|N_{\alpha }=0,\phi \right)\propto \exp\left(-w_{*}^{2}\right)G_{\alpha }^{*}\left(dw_{*}\right).$$

The proof builds on posterior characterizations for normalized CRMs (James, 2002, 2005; Prünster, 2002; Pitman, 2003; James *et al*., 2009) using the hierarchical construction of expression (29)–(30). See Appendix E.

The conditional distribution of $\left(w_{1},\ldots ,w_{N_{\alpha }},w_{*}\right)$ given ${\left(n_{ij}\right)}_{1⩽i,j⩽N_{\alpha }}$ does not depend on the locations $\left(\theta_{1},\ldots ,\theta_{N_{\alpha }}\right)$ because we considered a homogeneous CRM. This fact is important since the locations $\left(\theta_{1},\ldots ,\theta_{N_{\alpha }}\right)$ are typically not observed, and our algorithm outlined below will not consider these terms in the inference.

### 7.2 Markov chain Monte Carlo sampling for generalized gamma process based directed and undirected graphs

We now specialize to the case of the GGP, for which we derive an MCMC sampler for posterior inference. Let *ϕ*=(*α*,*σ*,*τ*) be the set of hyperparameters that we also want to estimate. We assume improper priors on the hyperparameters:

(43) $$\left.\begin{array}{c} p(\alpha )\propto 1/\alpha ,\\ p(\sigma )\propto 1/\left(1-\sigma \right),\\ p(\tau )\propto 1/\tau . \end{array}\right\}$$

To emphasize the dependence on the hyperparameters of the Lévy measure and distribution of the total mass w_*_, we write *ρ*(*w*|*σ*,*τ*) and $G_{\alpha ,\sigma ,\tau }^{*}\left(dw_{*}\right)$.

In the case of an undirected graph, we simply impute the missing directed edges in the graph. For each *i*⩽*j* such that *z*_*ij*_=1, we introduce latent variables ${\overset{¯}{n}}_{ij}=n_{ij}+n_{ji}$ with conditional distribution

(44) $${\overset{¯}{n}}_{ij}|z,w∼\left\{\begin{array}{cc} \delta_{0} & \text{if}\;z_{ij}=0,\\ \text{tPoisson}(2w_{i}w_{j}) & \text{if}\;z_{ij}=1,i\neq j, \end{array}\right.$$

and $n_{ii}|z_{ii}=1,w_{i}∼\text{tPoisson}\left(w_{i}^{2}\right)$, where tPoisson(*λ*) is the zero‐truncated Poisson distribution with probability mass function

$$\frac{\lambda^{k}\exp\left(-\lambda \right)}{\{1-\exp(-\lambda )\}k!},\text{for}\;k=1,2,\ldots .$$

By convention, we set ${\overset{¯}{n}}_{ij}={\overset{¯}{n}}_{ji}$ for *j*<*i* and $m_{i}=\Sigma_{j=1}^{N_{\alpha }}{\overset{¯}{n}}_{ij}$.

For scalable exploration of the target posterior, we propose to use HMC (Duane *et al*., 1987; Neal, 2011) within Gibbs sampling to update the weights $\left(w_{1},\ldots ,w_{N_{\alpha }}\right)$. The HMC step requires computation of the gradient of the log‐posterior, which in our case, letting *ω*_*i*_= log (*w*_*i*_), is given by

(45) $${\left[\nabla_{\omega_{1:N_{\alpha }}}\log\left\{p\left(\omega_{1:N_{\alpha }},w_{*}|D_{\alpha }\right)\right\}\right]}_{i}=m_{i}-\sigma -w_{i}\left(\tau +2\sum_{j=1}^{N_{\alpha }}w_{j}+2w_{*}\right).$$

For the update of the total mass w_*_ and hyperparameters *ϕ*, we use a Metropolis–Hastings step. Unless *σ*=0 or $\sigma =\frac{1}{2}$, $G_{\alpha ,\sigma ,\tau }^{*}\left(dw_{*}\right)$ does not admit any tractable analytical expression. We therefore use a specific proposal for w_*_ based on exponential tilting of $G_{\alpha ,\sigma ,\tau }^{*}$ that alleviates the need to evaluate this probability density function in the Metropolis–Hasting ratio (see the details in Appendix F). To summarize, the MCMC sampler is defined as follows.

*Step 1*: update the weights $\left(w_{1},\ldots ,w_{N_{\alpha }}\right)$ given the rest by using an HMC update.
*Step 2*: update the total mass w_*_ and hyperparameters *ϕ*=(*α*,*σ*,*τ*) given the rest by using a Metropolis–Hastings update.
*Step 3*: (undirected graph) update the latent counts $\left({\overset{¯}{n}}_{ij}\right)$ given the rest by using the conditional distribution (44) or a Metropolis–Hastings update.

The computational bottlenecks lie in steps 1 and 3, which roughly scale linearly in the number of nodes and edges respectively, although one can parallelize step 3 over edges. If *L* is the number of leapfrog steps in the HMC algorithm and *n*_iter_ the number of MCMC iterations, the overall complexity is in $O\{n_{\mathrm{iter}}\left(LN_{\alpha }+N_{\alpha }^{\left(e\right)}\right)\}$. We show in Section 8 that the algorithm scales well to large networks with hundreds of thousands of nodes and edges. To scale the HMC algorithm to even larger collections of nodes of edges, one could explore the methods of Chen *et al*. (2014).

## 8 Experiments

### 8.1 Simulated data

**Figure 9:**
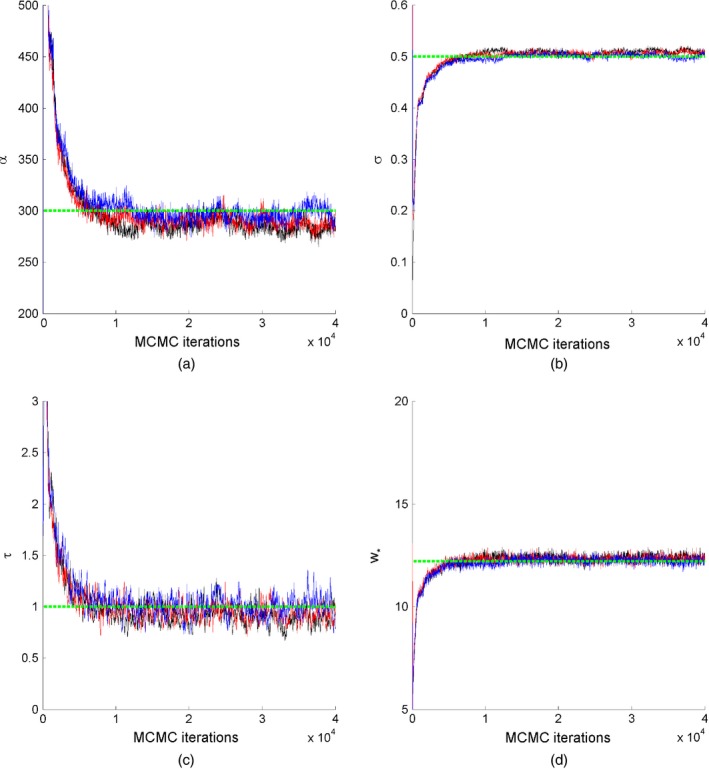
MCMC trace plots of parameters (a) *α*, (b) *σ*, (c) *τ* and (d) w_*_ for a graph generated from a GGP model with parameters *α*=300,*σ*=0.5 and *τ*=1: 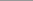, chain 1; 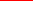, chain 2; 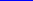, chain 3; 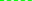, true

**Figure 10:**
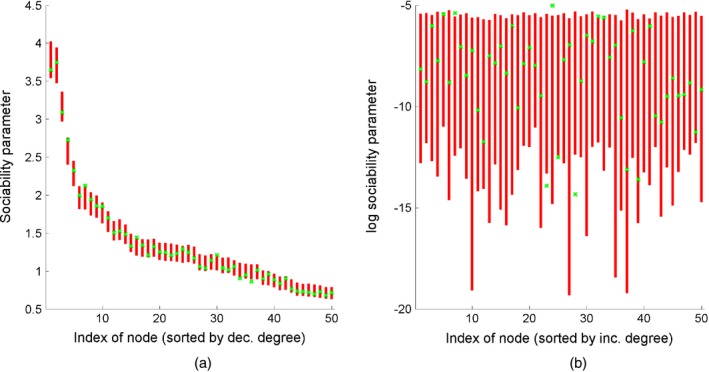
95% posterior intervals (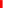) of (a) the sociability parameters *w*_*i*_ of the 50 nodes with highest degree and (b) the log‐sociability parameter  log (*w*_*i*_) of the 50 nodes with lowest degree, for a graph generated from a GGP model with parameters *α*=300,*σ*=0.5 and *τ*=1: 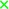, true values

We first study the convergence of the MCMC algorithm on simulated data where the graph is simulated from our model. We simulated a GGP undirected graph with parameters *α*=300, *σ*=0.5 and *τ*=1, which places us in the sparse regime. The sampled graph resulted in 13995 nodes and 76605 edges. We ran three MCMC chains each with 40000 iterations and with different initial values and *L*=10 leapfrog steps; the step size of the leapfrog algorithm was adapted during the first 10000 iterations to obtain an acceptance rate of 0.6. Standard deviations of the random‐walk Metropolis–Hastings steps for  log (*τ*) and  log (1−*σ*) were set to 0.02. The computing time for running the three chains successively was 10 min using a MATLAB implementation on a standard computer (central processor unit at 3.10 GHz; four cores). Trace plots of the parameters *α*,* σ*,* τ* and w_*_ are given in Fig. 9. We computed the potential scale factor reduction (Brooks and Gelman, 1998; Gelman *et al*., 2014) for all 13999 parameters $\left(\right.w_{1:N_{\alpha }},w_{*},\alpha ,\sigma$ and *τ*) and found a maximum value of 1.01, suggesting convergence of the algorithm. This is quite remarkable as the MCMC sampler actually samples from a target distribution of dimension 13995 + 76605 + 4 = 90604. Posterior credible intervals of the sociability parameters *w*_*i*_ of the nodes with highest degrees and log‐sociability parameters  log (*w*_*i*_) of the nodes with lowest degrees are displayed in Figs 10(a) and 10(b) respectively, showing the ability of the method to recover sociability parameters of both low and high degree nodes accurately.

To show the versatility of the GGP graph model, we now examine our approach when the observed graph is actually generated from an Erdoós–Rényi model with *n*=1 *pt*000 and *p*=0.01. The generated graph had 1000 nodes and 5058 edges. We ran three MCMC chains with the same specifications as above. In this dense graph regime, the following transformation of our parameters *α*,* σ* and *τ* is more informative: *ς*_1_=−(*α*/*σ*)*τ* ^*σ*^, *ς*_2_=−*σ*/*τ* and *ς*_3_=−*σ*/*τ* ^2^. When *σ*<0, *ς*_1_ corresponds to the expected number of nodes, *ς*_2_ to the mean of the sociability parameters and *ς*_3_ to their variance (see Section 6.3). In contrast, the parameters *σ* and *τ* are only weakly identifiable in this case. The potential scale reduction factor is computed on $\left(w_{1:N_{\alpha }},w_{*},\varsigma_{1},\varsigma_{2},\varsigma_{3}\right)$, and its maximum value was 1.01, suggesting convergence.

The value of *ς*_1_ converges around the true number of nodes and *ς*_2_ to the true sociability parameter $\surd \left\{-\frac{1}{2}\log\left(1-p\right)\right\}$ (constant across nodes for the Erdoós–Rényi model), whereas *ς*_3_ is close to 0 as the variance over the sociability parameters is very small. The total mass is also very close to 0, indicating that there are no nodes with degree 0.

**Figure 11:**
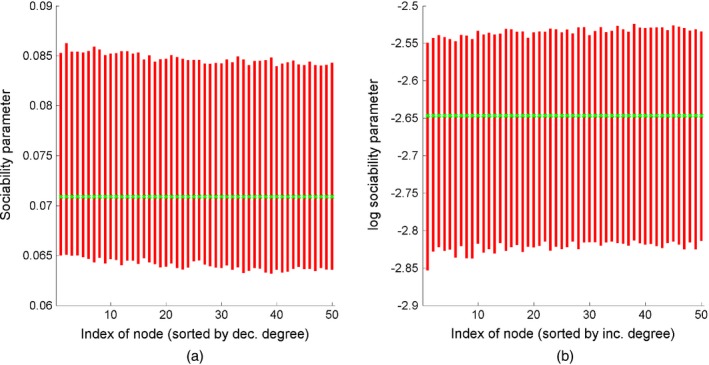
95% posterior intervals (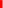) of (a) sociability parameters *w*_*i*_ of the 50 nodes with highest degree and (b) log‐sociability parameters  log (*w*_*i*_) of the 50 nodes with lowest degree, for a graph generated from an Erdoós‐Rényi model with parameters *n*=1000 and *p*=0.01: in this case, all nodes have the same true sociability parameter $\surd \left\{-\frac{1}{2}\log\left(1-p\right)\right\}$ (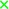)

Posterior credible intervals for the nodes with highest and lowest degrees are in Fig. 11, showing that the model can accurately recover sociability parameters of both low and high degree nodes in the dense regime as well.

### 8.2 Assessing properties of real world graphs

We now turn to using our methods to assess properties of a collection of real world graphs, including their degree distributions and aspects of sparsity. For the latter, evaluation based on a single finite graph is notoriously challenging as sparsity relates to the asymptotic behaviour of the graph. Measures of sparsity from finite graphs exist but can be costly to implement (Nešetřil and Ossona de Mendez, 2012). On the basis of our GGP‐based formulation and associated theoretical results described in Section 6, we consider Pr(*σ*⩾0|*z*) as informative of the connectivity structure of the graph since the GGP graph model yields dense graphs for *σ*<0, and sparse graphs for *σ* ∈ [0,1) (see equation (39)). For our analyses, we consider improper priors on the unknown parameters (*α*,*σ*,*τ*). We report Pr(*σ* ⩾ 0|*z*) based on a set of observed connections ${\left(z_{ij}\right)}_{1<i,j<N_{\alpha }}$, which can be directly approximated from the MCMC output. We consider 12 different data sets:

- *facebook107*—social circles in Facebook (https://snap.stanford.edu/data/egonetsFacebook.html) (McAuley and Leskovec, 2012);
- *polblogs*—political blogosphere (February 2005) (http://www.cise.ufl.edu/research/sparse/matrices/Newman/polblogs) (Adamic and Glance, 2005);
- *USairport*—US airport connection network in 2010 (http://toreopsahl.com/datasets/) (Colizza *et al*., 2007);
- *UCirvine*—social network of students at the University of California, Irvine (http://toreopsahl.com/datasets/) (Opsahl and Panzarasa, 2009);
- *yeast*—yeast protein interaction network (http://www.cise.ufl.edu/research/sparse/matrices/Pajek/yeast.html) (Bu *et al*., 2003);
- *USpower*—network of the high‐voltage power grid in the western states of the USA (https://snap.stanford.edu/data/emailEnron.html) (Watts and Strogatz, 1998);
- *IMDB*—actor collaboration network based on acting in the same movie (http://www.cise.ufl.edu/research/sparse/matrices/Pajek/IMDB.html);
- *cond‐mat1*—co‐authorship network (https://snap.stanford.edu/data/emailEnron.html) (Newman, 2001), based on preprints posted to condensed matter of arXiv between 1995 and 1999, obtained from the bipartite preprints–authors network using a one‐mode projection;
- *cond‐mat2*—as in cond‐mat1, but using Newman's projection method;
- *Enron*—Enron collaboration network from a multigraph e‐mail network (https://snap.stanford.edu/data/emailEnron.html);
- *internet*—connectivity of Internet routers (http://www.cise.ufl.edu/research/sparse/matrices/Pajek/internet.html);
- *www*—linked World Wide Web pages in the nd.edu domain (http://lisgi1.engr.ccny.cuny.edu/~makse/soft_data.html).

**Figure 12:**
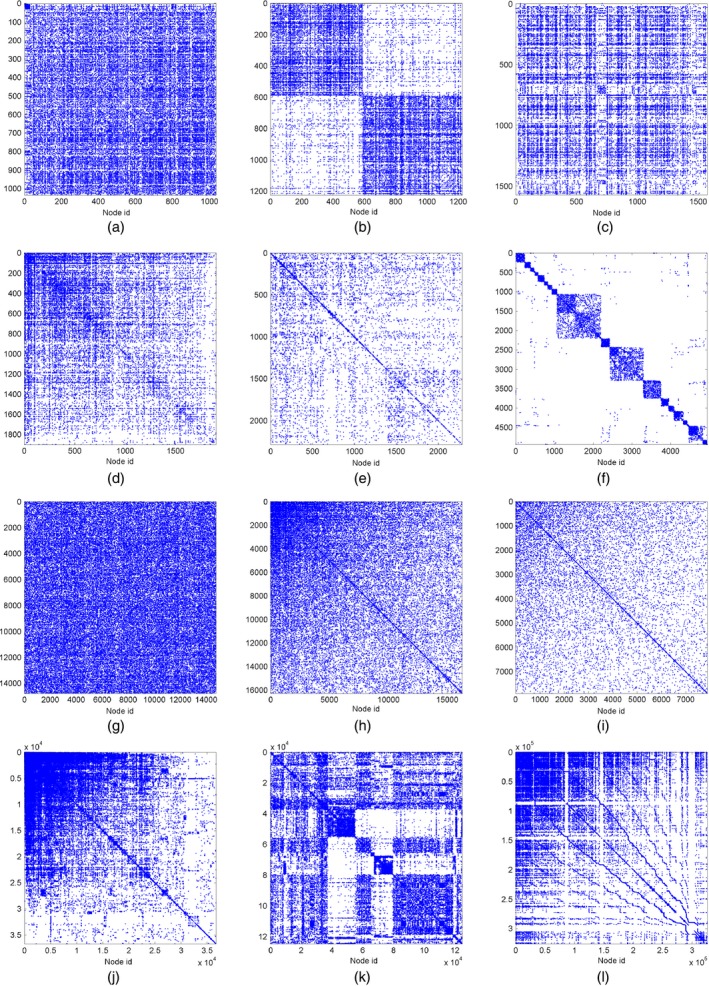
Adjacency matrices for various real world networks: (a) facebook107; (b) polblogs; (c) USairport; (d) UCirvine; (e) yeast; (f) USpower; (g) IMDB; (h) cond‐mat1; (i) cond‐mat2; (j) Enron; (k) internet; (l) www

**Figure 13:**
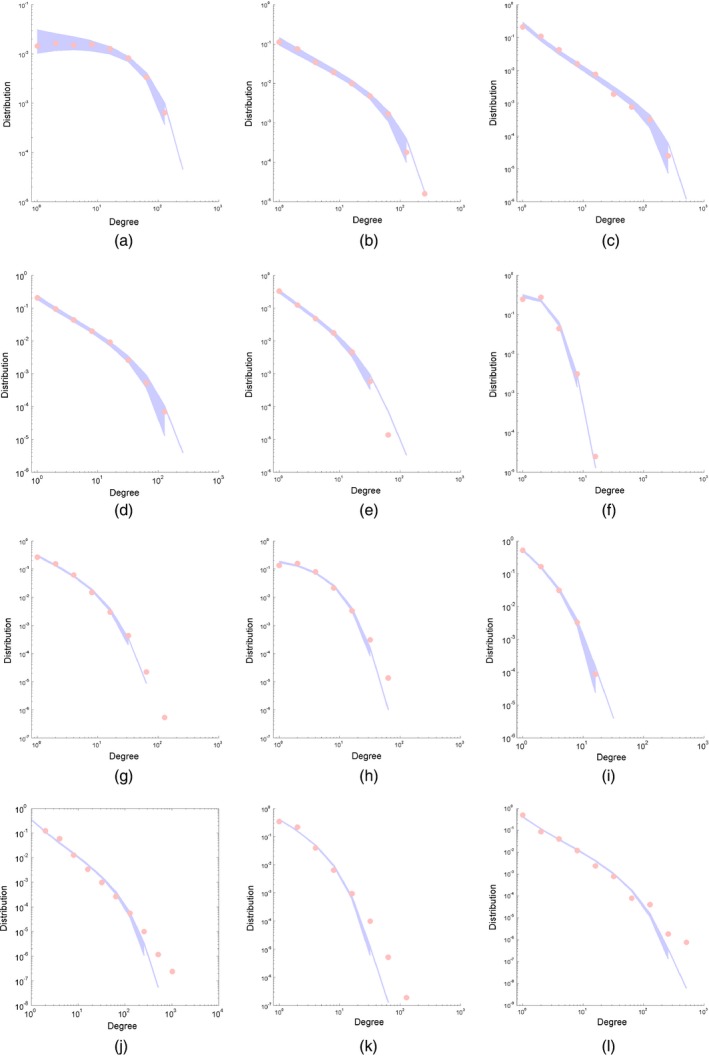
Empirical degree distribution (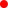) and posterior predictive (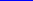) for various real world networks: (a) facebook107; (b) polblogs; (c) USairport; (d) UCirvine; (e) yeast; (f) USpower; (g) IMDB; (h) cond‐mat1; (i) cond‐mat2; (j) Enron; (k) internet; (l) www

The sizes of the various data sets are given in Table 2 and range from a few hundred nodes or edges to a million. The adjacency matrices for these networks are plotted in Fig. 12 and empirical degree distributions in Fig. 13 (red).

**Table 2:** Size of real world data sets and posterior probability of sparsity

*Data set*	*Number of*	*Number of*	*Time*	*Pr*(*σ*⩾0|*z*)	*99% credible*
	*nodes*	*edges*	*(min)*		*interval σ*
facebook107	1034	26749	1	0.00	[−1.06,−0.82]
polblogs	1224	16715	1	0.00	[−0.35,−0.20]
USairport	1574	17215	1	1.00	[0.10,0.18]
UCirvine	1899	13838	1	0.00	[−0.14,−0.02]
yeast	2284	6646	1	0.28	[−0.09,0.05]
USpower	4941	6594	1	0.00	[−4.84,−3.19]
IMDB	14752	38369	2	0.00	[−0.24,−0.17]
cond‐mat1	16264	47594	2	0.00	[−0.95,−0.84]
cond‐mat2	7883	8586	1	0.00	[−0.18,−0.02]
Enron	36692	183831	7	1.00	[0.20, 0.22]
internet	124651	193620	15	0.00	[−0.20,−0.17]
www	325729	1090108	132	1.00	[0.26,0.30]

We ran three MCMC chains for 40000 iterations with the same specifications as above and report the estimate of Pr(*σ*⩾0|*z*) and 99% posterior credible intervals of *σ* in Table 2; we additionally provide run times. MCMC trace plots suggested rapid convergence of the sampler. Since sparsity is an asymptotic property of a graph, and we are analysing finite graphs, our inference of *σ* here simply provides insight into some structure of the graph and is not formally a test of sparsity. From Table 2, we note that we infer negative *σ*‐values for many of the smaller networks. This might indicate that these graphs have dense connectivity; for example, our facebook107 data set represents a small social circle that is probably highly interconnected and the polblogs data set represents two tightly connected political parties. We infer positive *σ*‐values for three of the data sets (USairport, Enron and www); note that two of these data sets are in the top three largest networks considered, where sparse connectivity is more commonplace. In the remaining large network, internet, a question is why the inferred *σ* is negative. This may be due to dense subgraphs or spots (for example, spatially proximate routers may be highly interconnected, but sparsely connected outside the group) (Borgs *et al*., 2014b). This relates to the idea of *community structure*, though not every node need be associated with a community. As in many sparse network models that assume no dense spots (Bollobás and Riordan, 2009; Wolfe and Olhede, 2013), our approach does not explicitly model such effects. Capturing such structure remains a direction of future research that is likely to be feasible within our generative framework. However, our current method has the benefit of simplicity with three hyperparameters tuning the network properties. Finally, we note in Table 2 that our analyses finish in a remarkably short time although the code base was implemented in MATLAB on a standard desktop machine, without leveraging possible opportunities for parallelizing and other mechanisms for scaling the sampler (see Section 7 for a discussion).

To assess our fit to the empirical degree distributions, we used the methods that were described in Section 5.5 to simulate 5000 graphs from the posterior predictive distribution in each scenario. Fig. 13 provides a comparison between the empirical degree distributions and those based on the simulated graphs. In all cases, we see a reasonably good fit. For the largest networks, Figs 13(j)–13(l), we see a slight underestimate of the tail of the distribution, i.e. we do not capture as many high degree nodes as truly present. This may be because these graphs exhibit a power law behaviour, but only after a certain node degree (Clauset *et al*., 2009), which is not an effect that is explicitly modelled by our framework. Instead, our model averages the error in the low and high degree nodes. Another reason for underestimating the tails might be dense spots, which we also do not explicitly model. However, our model does capture power law behaviour with possible exponential cut‐off in the tail. We see a similar trend for cond‐mat1, but not cond‐mat2. Based on the bipartite articles–authors graph, cond‐mat1 uses the standard one‐mode projection and sets a connection between two authors who have co‐authored a paper; this projection clearly creates dense spots in the graph. In contrast, cond‐mat2 uses Newman's projection method (Newman *et al*., 2001). This method constructs a weighted undirected graph by counting the number of papers that were co‐authored by two scientists, where each count is normalized by the number of authors on the paper. To construct the undirected graph, an edge is created if the weight is equal to or greater than 1; cond‐mat1 and cond‐mat2 thus have a different number of edges and nodes, as only nodes with at least one connection are considered. It is interesting that the projection method that was used for the cond‐mat data set has a clear influence on the sparsity of the resulting graph, cond‐mat2 being less dense than cond‐mat1 (see Figs 13(h) and 13(i)). The degree distribution for cond‐mat1 is similar to that of internet, thus inheriting the same issues as previously discussed. Overall, it appears that our model better captures homogeneous power law behaviour with possible exponential cut‐off in the tails than it does a graph with perhaps structured dense spots or power law after a point behaviour.

## 9 Conclusion

We proposed a class of statistical network models building on exchangeable random measures. Using this representation, we showed how it is possible to specify models with properties that are different from those of models based on exchangeable adjacency matrices. As an example, we considered a model building on the framework of CRMs that can yield sparse graphs while maintaining attractive exchangeability properties. For a choice of CRMs, our fully generative formulation can yield networks ranging from dense to sparse, as tuned by a single hyperparameter.

In this paper, exchangeability is in the context of random measures for which we appealed to the Kallenberg representation in place of the Aldous–Hoover theorem for exchangeable arrays. Using this framework, we arrived at a structure that is analogous to the graphon, which opens up new modelling and theoretical analysis possibilities beyond those of the special case that is considered herein. Importantly, through the exchangeability of the underlying random measures and leveraging HMC sampling, we devised a scalable algorithm for posterior computations. This scheme enables inference of the graph parameters, including the parameter determining the sparsity of the graph. We examined our methods on a range of real world networks, demonstrating that our model yields a practically useful statistical tool for network analysis.

We believe that the foundational modelling tools and theoretical results that we presented represent an important building block for future developments. Such developments can be divided along two dimensions:

- modelling advances, such as incorporating notions of community structure and node attributes, within this framework and
- theoretical analyses looking at the properties of the corresponding class of networks.

For the latter, we considered just one simplified version of the Kallenberg representation; examining a more general form could yield graphs with additional structure. Building on an initial version of this paper (Caron and Fox, 2014), initial forays into advances on the modelling side can be found in Herlau *et al*. (2016) and Todeschini and Caron (2016) and theoretical analyses in Veitch and Roy (2015) and Borgs *et al*. (2016).

## Discussion on the paper by Caron and Fox

**Ginestra Bianconi** (*Queen Mary University of London*)

I am delighted and honoured to open the discussion on this paper which provides an ideal starting point to reflect on the benefits provided by the interdisciplinary character of network science.

From brain research to social networks ‘big data’ in the form of networks are permeating the sciences and our own everyday life. Therefore there is an urgent need to develop new methods and techniques to extract information from network data. Network science has developed as the fast growing interdisciplinary field that addresses this problem with the aim of obtaining predictive models for social, physical and biological phenomena occurring in networks.

The success of network science (Barabási, 2016; Newman, 2010; Dorogovtsev and Mendes, 2002) is rooted in the following two characteristics of the field:

- the ubiquitous presence of networks describing complex interacting systems in social, technological and biological contexts;
- the ability of the field to adopt methods and techniques coming from different theoretical disciplines such as statistical mechanics, graph theory and statistical network modelling.

Although already at the beginning of the field the first aspect was shown to be essential for the characterization of the universal properties of networks (Barabási and Albert, 2009; Watts and Strogatz, 1998), more recently it has become clear that network science can lead to a comprehensive understanding of network phenomena only if the methods and techniques that are used to study networks reflect different theoretical perspectives.

Initially statistical mechanics has been the most prosperous and successful approach to network modelling. In this framework we distinguish between a non‐equilibrium approach of growing network models evolving through the *preferential attachment* rule (Dorogovtsev and Mendes, 2002; Barabási and Albert, 2009) and equilibrium approaches characterizing network ensembles enforcing either hard constraints (such as the configuration model) or soft constraints (such as exponential random graphs) (Park and Newman, 2004; Anand and Bianconi, 2009, 2010).

Most statistical mechanics models describe the regime in which the average degree 〈*k*〉 does not depend on the number of nodes *N*, i.e. 〈*k*〉=*O*(1). Particular focus has been addressed to scale‐free networks in this regime having degree distribution *P*(*k*)≃*Ck* ^−*γ*^ for *k*≫1 and a power law exponent in the range *γ* ∈ (2,3]. The particular emphasis given to the regime in which the average degree is constant with increasing network size is justified by the fact that indeed the vast majority of network data from the Internet to actor collaboration networks belong to this class of networks (Barabási, 2016).

However, there is evidence that several network data from social on‐line networks (Seyed‐Allaei *et al*., 2006) to neuroscience (Bonifazi *et al*., 2009) have a power law degree distribution *P*(*k*)≃*Ck* ^−*γ*^ with *γ* ∈ (1,2] implying very heterogenous degrees, very significant hubs and a diverging average degree. Despite the recent interest in these classes of networks (Seyed‐Allaei *et al*., 2006; Lambiotte *et al*., 2016; Timár *et al*., 2016) historically these networks have been disregarded or neglected in the statistical mechanics community (Barabási, 2016; Del Genio *et al*., 2011).

From the statistical perspective, recently we have been witnessing a new renaissance of network modelling (Goldenberg *et al*., 2010) emphasizing the relevance of having exchangeable and projective network models. Exchangeable models guarantee that the order in which nodes are sampled is irrelevant. Projective models guarantee that a network inference performed on a sample of *N* nodes can be used to infer properties of a sample obtained including more nodes. Interestingly the statistical mechanics models including non‐equilibrium growing network models and network ensembles do not have either of these two properties.

Exchangeable projective models generated by a joint exchangeable adjacency matrix are described by the *graphon* and generate dense networks where the average degree is increasing linearly with the network size, i.e. 〈*k*〉=*O*(*N*). Given a joint exchangeable adjacency matrix the Aldous–Hoover representation can be expressed as the existence of a graphon, implying that the network is dense 〈*k*〉=*O*(*N*). Since the vast majority of real network data sets are sparse and have an average degree increasing sublinearly with the number of nodes, one of the major problems of the field was to overcome this limitation of the graphon.

In their paper Caron Fox formulate for the first time a generative exchangeable and projective model for sparse networks.

The major step to overcome the limitation of the graphon has been considering a point process on the plane instead of an exchangeable matrix. Thanks to the Kallenberg representation theorem for random measurable functions this model admits a representation as a mixture of random functions that naturally extend the graphon to the sparse regime.

Caron and Fox's model, based on the assumption of the existence of a latent space (the sociability of a node), achieves the following three major results.

- The model generates either dense networks with 〈*k*〉=*O*(*N*) or sparse networks with 〈*k*〉=*O*(*N* ^*θ*^) and *θ* ∈ (0,1). Therefore the model constitutes a significant advance on exchangeable and projective statistical network modelling.
- The model generates scale‐free networks with diverging average degree
  (76) $$P\left(k\right)≃Ck^{-\gamma }$$
  and *γ* ∈ (1,2]. Therefore the model shows that in the framework of statistical network modelling these networks emerge *naturally*, enabling us to describe networks that have been historically neglected in the statistical mechanics approach to networks.
- The model enables an efficient method for inference of network data. Analysed network data include network data sets of sizes up to *N*= 300000 nodes. It should be noted that the code is freely available from the author's Web page.

In conclusion Caron and Fox's paper opens a new scenario in statistical network modelling enabling the treatment of exchangeable and projective sparse network models. Additionally this work is a beautiful example of the benefits that can be obtained by an interdisciplinary approach to network science.

Many prospects can be envisaged for future research. These include the extension to more general latent spaces (Todeschini and Caron, 2016), the proposal of new network exchangeable models to preserve privacy (Borgs *et al*., 2015) and the investigation of the regime 〈*k*〉=*O*(1) for exchangeable network models that would finally clarify whether it is possible to have a global framework to study both networks with constant average degree and diverging average degree.

It therefore gives me great pleasure to propose the vote of thanks.

**Karthik Bharath** (*University of Nottingham*)

I applaud Caron and Fox's efforts in the paper for developing a novel approach to represent and model networks based on point processes, which since its first version in 2014 has inspired several new forays into the literature on graph limits. In a certain sense the authors’ approach is reminiscent of the *objective method* (Aldous and Steele, 2004) for combinatorial objects, and continuum limits of discrete objects, wherein of chief interest is the study of local properties of the graphs unlike global properties like sparsity. However, the utility of such probabilistically interesting continuous objects as statistical models for network or graph data is suspect; this was recently explored in Bharath *et al*. (2017) in the context of tree‐structured data (acyclic graphs) using the continuum random tree (Aldous, 1993). My own view is that statistical models that aim to capture and quantify variability in characteristics of real world networks need necessarily be compatible with its inherently discrete nature, and the fundamental statistical notion of sampling from a population network (e.g. adding or deleting vertices).

I am hence sceptical of the conceptual underpinning of their approach from a purely modelling perspective: the appropriateness of the notion of exchangeability—which is the *leitmotiv* of the paper—of point processes (within the Kallenberg framework) for network models is unclear. Depending on the question of interest the basic statistical datum on a network is identified (vertex, edge, triangle or other motifs), and exchangeability is then defined as (probabilistic) invariance to their labelling, which usually is arbitrary. The authors’ continuous space representation of a network as a point process $Z=\Sigma_{i,\;j}z_{ij}\delta \left(\theta_{i},\theta_{j}\right)$ on the plane implies that the distribution of the number of points that fall within rectangles *A*_*i*_×*A*_*j*_ where *A*_*i*_:=[*h*(*i*−1),*hi*],*h*>0, is invariant to *relabelling of the rectangles* formed by intervals. This begs the question: what exactly is exchangeable on an observed network with vertices and edges represented by the measure‐valued process {*Z*_*a*_,*α*>0}? The answer becomes trickier when isolated vertices are deleted, as done in the paper. In fact the sequence of networks {*Z*_*α*_,*α*>0} is unlabelled for the observed atoms! A similar issue also arises in the desirable notion of projectivity under subsampling: for $\alpha_{2}<\alpha_{1}$ projectivity of the restriction of the law of $Z_{\alpha_{1}}$ to [0,*α*_2_]^2^ is not compatible with operations such as deletion and addition of vertices or edges, and I am unsure whether a sensible analogous interpretation is available within the point process setting. In this context recently developed notions of edge and relational exchangeability appear particularly cogent (Crane and Dempsey, 2015, 2016).

The flexibility in their approach to generate dense and sparse graphs governed by the choice of the Lévy measure is attractive and points towards development of interesting inferential tools for networks. The data sets that are considered in the paper are pregnant with interesting questions on functional relationships between vertices and transport phenomena in networks that are indicative of its functionality. Disappointingly, the authors restrict their attention only to assessing whether a network is sparse. The apparent utility (or lack thereof) of the point process representation for inference on real networks is not examined at all. For example, extreme events on the USairport or www data sets could be formulated by using random walks on the network. A vertex could be classified as experiencing an extreme event if the average number of walks traversing it at a given time is greater than some threshold (Kishore *et al*., 2011). The average then could be computed with respect to the stationary distribution of a Markov chain with an *n*‐step transition probability of a walk from vertex *i* to vertex *j*, given by

$$P_{ij}\left(n+1\right)=\sum_{r}\;\left\{1-\exp\left(2w_{r}w_{j}\right)\right\}P_{ir}\left(n\right).$$

Posterior samples of *w*_*i*_ could conceivably be used in estimating the stationary distribution, which then allows for probabilistic statements of such extreme events. Assessing power law behaviour through the double‐logarithm plot is shown to be dubious in Clauset *et al*. (2009); surprisingly, the authors use this method of assessment despite referring to Clauset *et al*. (2009). Can the overall model fit be assessed through a posterior predictive check?

The interpretation (and identifiability) of *α* in *Z*_*α*_ within the context of an observed network is not clear since *α* is the upper bound for the $\theta_{i}s$—this is exacerbated in the sparse regime when *α*→∞. The interpretation of the credible intervals for the *σ*‐parameter (and hence the question of sparsity) is also unclear since the parameter *α* is unobserved and is estimated. It might be interesting to explore the structural properties of the graphs if the completely random measure *W* is allowed to be inhomogeneous such that *v*(d*w*,d*θ*)=*ρ*(d*w*,*θ*)*λ*(d*θ*); indeed, this brings about additional computational and interpretability issues. Despite the elegant theoretical foundation, the point process model for a *finite* network is perhaps ‘too rich’—whereas the Lévy measure *ρ* and jumps *w*_*i*_ are profitably used, the latent parameters $\theta_{i}$ (whose natural ordering on $R^{+}$ is discarded in the interests of the exchangeability) and *α* throw up some uncomfortable questions on model interpretability and identifiability.

The vote of thanks was passed by acclamation.

**Wilfrid S. Kendall** (*University of Warwick, Coventry*)

Caron and Fox are to be congratulated on producing an inspiring and elegant blend of the creative probabilistic theory of exchangeability and the practical demands of statistical network analysis. I am impressed by the way in which practical considerations of tunability guide them towards exchangeable and completely random measures and theorems of Kallenberg and Aldous–Hoover.

Here I share recent work on rather different random networks, having a pronounced spatial element. Aldous (Aldous and Ganesan, 2013; Aldous, 2014) proposed an axiomatization of what one might call a scale invariant random spatial network (SIRSN). Motivated by on‐line maps, one postulates a random entity producing a unique set of connections *N*(**x**_1_,…,**x**_*n*_) between any prescribed set of points **x**_1_,…,**x**_*n*_ on the plane: *N*(**x**_1_,…,**x**_*n*_) is composed of routes **r**(**x**_*i*_,**x**_*j*_) between each pair of points **x**_*i*_ and  **x**_*j*_. It is required that the network be statistically invariant under translation, rotation and scaling, that the mean distance of a route between two specified points be finite and, finally, that if one considers a network connecting all points of an (independent) Poisson point process of intensity *λ* then the ‘long distance’ parts of the routes (say, **r**(**x**_*i*_,**x**_*j*_)∖ball(**x**_*i*_,1)∖ball(**x**_*j*_,1) should form a fibre process of finite mean length per unit area. (This is the ‘weak SIRSN property’; the ‘strong SIRSN property’ requires the bound on mean length per unit area to hold uniformly in *λ*.) In effect, long distance routes are reused by many different point pairs

It is a highly non‐trivial matter to prove that SIRSNs can exist, but we now have two different examples. Aldous (2014) produced a construction based on randomized grids. Kahn (2016) and Kendall (2017) put together arguments which showed that an SIRSN can be constructed using Poisson line processes—improper scale invariant line processes marked by speeds, with routes formed using fastest possible paths in the network. Remarkably, the Poisson line process construction also works in 3‐space and beyond.

It is salutary to compare this with the work of Caron and Fox. SIRSNs provide toy models for real world spatial transportation networks, and a theoretical justification for the helpful notion of ‘transit nodes’ (Bast *et al*., 2007). But, although there is typically a single scalar parameter *γ* expressing the scale invariance, current SIRSN models cannot be said to be very tunable, nor to accommodate possibilities for data modelling; a clear challenge for future work.

**Benjamin Bloem‐Reddy** (*Columbia University, New York*)

Research in this area is progressing rapidly, and there are connections to some concurrent work. See also Janson (2017a), section 5.1, for related ideas. For every model based on an exchangeable random measure (ERM) (see also Veitch and Roy (2015, 2016), Borgs *et al*. (2016) and Janson (2016)), we can construct a so‐called *edge exchangeable* model (Crane and Dempsey, 2015, 2016; Williamson, 2016; Cai *et al*., 2016; Janson, 2017a) that coincides with a finite restriction of the ERM model. The converse is true only in some cases (see Cai *et al*. (2016) for an example). I consider the completely random measure (CRM) case for concreteness.

Let *X*_1_,*X*_2_,… be an exchangeable sequence of edges, and let $E_{n}$ be the directed multigraph composed of the first *n* edges labelled in order of appearance. If the edge(s) connecting a pair of vertices are given a unique ‘colour’, Kingman's paintbox theorem shows that every such graph can be generated by sampling

$$\Phi ∼\mu \;\;\text{and}\;\;X_{i}|\Phi \overset{\mathrm{IID}}{∼}\Phi \;\;\text{for}\;\;i=1,\ldots ,n,$$

where Φ is a random discrete probability measure sampled from a mixing measure *μ* (Crane and Dempsey, 2016). Denote such a graph by $E_{n}^{\Phi }$.

Consider when Φ is the normalized CRM product measure (see section 5.5.1) $\Phi_{\alpha }:=W_{\alpha }\times W_{\alpha }/W_{\alpha }^{*2}$. Then the graph *D*_*α*_, with $D_{\alpha }^{*}$ edges, and $E_{D_{\alpha }^{*}}^{\Phi_{\alpha }}$ have the same conditional law,

$$L\left(D_{\alpha }|W_{\alpha },D_{\alpha }^{*}\right)=L\left(E_{D_{\alpha }^{*}}^{\Phi_{\alpha }}|\Phi_{\alpha },D_{\alpha }^{*}\right).$$

If *μ* places mass only on normalized CRMs with mean measure *ρ*(d*w*)*λ*_*α*_(d*θ*), then equality in distribution holds unconditionally. However, an ERM model and its counterpart edge exchangeable model coincide only for a particular *α*. *D*_*α*_ and $E_{D_{\alpha }^{*}}^{\Phi_{\alpha }}$ grow differently: fix *ɛ*>0, and let *D*_*α*_ grow to *D*_*α*+*ɛ*_, denoting by $D_{\alpha ,\;\varepsilon }^{*}:=D_{\alpha +\varepsilon }^{*}-D_{\alpha }^{*}$ the number of additional edges. Then

$$L\left(D_{\alpha +\varepsilon }|D_{\alpha },\;W_{\alpha +\varepsilon },D_{\alpha ,\;\varepsilon }^{*}\right)\neq L\left(E_{D_{\alpha }^{*}+D_{\alpha ,\;\;\varepsilon }^{*}}^{\Phi_{\alpha }}|E_{D_{\alpha }^{*}}^{\Phi_{\alpha }},\Phi_{\alpha },D_{\alpha ,\;\varepsilon }^{*}\right).$$

The inequality reflects a fundamental difference in how the two model classes encode the notion of growth and offers guidance for choosing an appropriate model class. Edge exchangeable models posit that graphs grow one edge at a time; growth in ERM models is by a random number of edges as *α* increases. As a consequence, in an edge exchangeable graph growing to arbitrary size, an edge may occur between two vertices that were previously not connected. This is not true for ERM models, which require that new edges form only between new vertices, inducing a growing population of edges from which to sample. Conversely, edge exchangeable models sample from a fixed (possibly infinite) population.

**Patrick Rubin‐Delanchy** (*University of Bristol*)

This is a very creative and thought‐provoking paper on network modelling, opening many avenues for future research. With this second point in mind, it would be useful to set down formally what this new definition of graph exchangeability should be in general: is an arbitrary model for an infinite graph (*z*_*ij*_)_*i*,*j*=1,2…_ exchangeable in this sense if *there is a coupling* $\left(\left(z_{ij}\right),{\left(\theta_{i}\right)}_{i=i,\;2,\;\ldots }\right)$ so that, marginally, *z* follows the specified model and ($\theta_{i}$) is a unit rate Poisson process, and, jointly, the point process $\Sigma z_{ij}\delta \left(\theta_{i},\theta_{j}\right)$ is exchangeable?

Some popular approaches to network modelling rely on latent positions (*X*_*i*_)_*i*=1,2_,… for each of the nodes, where, conditionally on *X*_*i*_, we have *z*_*ij*_∼^ind^ Bernoulli {*f*(*X*_*i*_,*X*_*j*_)} for each *i* and *j* for some function *f*. Under the usual infinite exchangeability assumption, the Aldous–Hoover theorem suggests a Bayesian treatment of *f*, and the *X*_*i*_ are independent uniform positions on [0,1]. However, Hoff *et al*. (2002) assumed that *f*(*X*_*i*_,*X*_*j*_) is logistic in the distance ‖*X*_*i*_−*X*_*j*_‖ (ignoring covariates), with the *X*_*i*_ independent and identically distributed (IID) on $R^{d}$ (this being part of the prior rather than the model), and the random dot product graph (Nickel, 2006; Young and Scheinerman, 2007; Athreya *et al*., 2016) assumes that $f\left(X_{i},X_{j}\right)=X_{i}^{T}X_{j}$, with the *X*_*i*_ IID on a convex set $X\subset R^{d}$. The stochastic block model (Holland *et al*., 1983) and mixed membership stochastic block model (Airoldi *et al*., 2008) can also be written as latent position models. In the present paper, such models were mentioned as possible extensions where, if I understand correctly, the $\theta_{i}$ could represent these latent positions. Instead, I think of the *w*_*i*_ as latent positions, and indeed in a follow‐up paper these parameters live in higher dimensions (Todeschini and Caron, 2016). What I expect in general is that the assumption of IID latent positions, present in each of the earlier models, must be dispensed with, and instead the *X*_*i*_ must now form a more complicated point process on $R^{d}$ (or whichever space is appropriate), just as the *w*_*i*_ are not IID but instead form a more complex point process on the positive line.

It is then interesting to ask how are estimates of these positions affected, starting with the simplest question of whether even the *w*_*i*_ in this paper are estimated consistently? But, to give a more interesting example, for the random dot product graph, Athreya *et al*. (2016) have shown that the estimation error under adjacency spectral embedding goes as the usual rate *n* ^−1/2^ and is asymptotically Gaussian, but here the IID assumption on the *X*_*i*_ is important. If the graph is ‘sparsified’, using the ideas of this paper, how are these spectral estimates affected?

The following contributions were received in writing after the meeting.

**Julyan Arbel** (*Inria Grenoble Rhône‐Alpes*)

The paper by Caron and Fox is a very fine methodological work which illustrates once again the huge modelling flexibility and versatility of discrete Bayesian non‐parametric priors. They target here sparsity in graphs, the level of which can be neatly assessed according to the stability parameter *σ* of the discrete process under consideration.

The posterior distribution of *σ* is notoriously highly concentrated in the context of Bayesian non‐parametric inference for species sampling problems. The credible intervals’ narrowness obtained for the real world graphs suggests that the same holds here. Caron and Fox validate their methodology via posterior predictive checks such as the fit to the empirical degree distribution. Another type of validation, theoretical though, which is not considered by them is through *posterior consistency*. In the present setting, the graph is given and interest is in assessing graph properties such as sparsity. Posterior consistency here amounts to asking whether the model is capable of recovering a sparsity index *σ*, in other words: if the true graph‐generative process is assumed to have a sparsity index *σ*_0_, then does the posterior of a contract to a point mass at *σ*_0_ when the size of the graph increases to ∞? The sparsity index *σ*_0_ can be defined in the spirit of equation (21) by the asymptotic relationship between the number of nodes $N_{\alpha }$ and the number of edges $N_{\alpha }^{\left(e\right)}$:

(77) $$N_{\alpha }^{\left(e\right)}/N_{\alpha }^{2/\left(1+\sigma_{0}\right)}\to 1$$

as the graph size grows to ∞. According to the definitions given in equations (17) and (18), the *graph size* can be equivalently measured by *α*, $N_{\alpha }$ or $N_{\alpha }^{\left(e\right)}.$ The true graph‐generative process could be the generalized gamma process itself (well‐specified setting) or any other graph process satisfying condition (77) for some *σ*_0_ (misspecified setting).

In recent research (Arbel *et al*., 2017), we introduce conditions on the Lévy intensity of the completely random measure to ensure consistent estimation in species sampling problems. Though the setting is quite different, our conditions are similar to the tail assumptions made by Caron and Fox in the derivation of $N_{\alpha }$ and $N_{\alpha }^{\left(e\right)}$ asymptotic behaviours. Admittedly, the consistency assumption of a true generative model with a given fixed level of sparsity is an idealized assumption which cannot account for real world graphs oddities such as local effects underlined by the authors: dense subgraphs (spots) and community structure. However, we believe that consistency properties could shed some light on why *σ* were estimated to be negative for most of the real world applications in the paper, thus concluding on dense graphs.

**Sayantan Banerjee** (*Indian Institute of Management Indore*) **and Subhashis Ghosal** (*North Carolina State University, Raleigh*)

Caron and Fox are to be congratulated for an interesting construction of random graphs through completely random measures on the real line and also for extending the ideas to directed multigraphs through Poisson processes with intensity measures as the product of completely random measures on the real line. Sociability parameters are used to generate edges which are further generated through completely random measures. The most interesting aspect of such constructions is the dichotomy of density or sparsity, depending on finite or infinite mass of the intensity measure of the completely random measure driving the construction. In particular, the example of the generalized gamma process is fascinating since the whole spectrum of activity can be captured through a single tuning parameter. In this context, it will be interesting to characterize the normalized graph Laplacian and its eigenvalues which give valuable information on the number of connected components. Using some appropriate graph partitioning algorithm (see for example, Von Luxburg (2007)), different communities in the graph are possible to detect. It will be interesting to study the probabilistic behaviour of the communities thus obtained from a random graph constructed by the authors. This may facilitate scale‐dependent community detection in graphs, where the different scales refer to the local or global neighbourhood of the individual nodes of the graph. For example, in the case of a Swiss roll manifold generated from Gaussian mixtures, some scales can recover the underlying Gaussian components and some scales can detect communities which respect the underlying geometry of the manifold.

From a more general perspective, the model proposed by the authors seems to be an interesting way of selecting a particular graphon model *P*(*z*_*ij*_=1)=*g*(*U*_*i*_,*U*_*j*_) for the probability of an edge through latent variables *U*_1_,*U*_2_,…. By setting a particular point a reference point, tying the graphon to zero at it and making the latent variables concentrate near this point should induce sparsity. Can this general graphon model be obtained through a transformation on the plane from the model proposed by the authors? If so, can this connection be used to characterize the sparsity‐generating mechanism in the general graphon model?

Another question potentially of interest and topic of further study is the limiting distribution of normalized numbers of edges. A normal approximation in the dense case and a Poisson approximation in the sparse case are expected.

**Marco Battiston** (*Oxford University*) **andStefano Favaro** (*University of Turin and Collegio Carlo Alberto, Turin*)

We congratulate Caron and Fox for their interesting contribution, which has already attracted much interest in the statistical community. Here we would like to point to new developments related to privacy issues in network modelling. Network data usually contain sensitive information about individuals, e.g. medical status, wages, friendships or sexual or political preferences. A noteworthy example of privacy disclosure is in Narayanon and Shmatikov (2009), who showed how to identify users in the Netflix data set, which can be modelled by a weighted bipartite graph, even after users and movies labels had been removed. Privacy problems are concerned with providing mechanisms to transform raw data into a privatized data set to be released. A popular measure to check whether a mechanism can privatize a data set is *differential privacy,* which was initially proposed in Dwork *et al*. (2006) and recently considered in graph theory for network data. A mechanism A is said to be *node private* if an intruder looking at the output released by the mechanism cannot correctly guess with high probability whether a node (individual) is in the data set or is not and figure out which are his edges (links). A formal definition of *ε‐node privacy* is that, for all subsets *S* of the output space, the mechanism A must satisfy

(78) $$\text{Pr}\left\{A\left(G\right)\in S\right\}⩽\exp\left(\epsilon \right)\text{Pr}\left\{A\left(G^{\prime }\right)\in S\right\}$$

for all graphs *G* and *G* ^′^ which can be obtained one from another by removing a vertex and its adjacent edges.

To our knowledge, the only attempt to study an *ε*‐*node private* mechanism is in Chayes *et al*. (2015). They considered sparse graphs obtained by rescaling a dense graphon with the network size, and they proposed a mechanism that releases as output a step graphon that satisfies condition (78). We believe that an interesting line of research would be to study how privacy constraints could be handled within the sparse graphs setting proposed by Caron and Fox. Specifically, is *ε*‐node privacy a good measure of disclosure for graphs or are better notions needed in the sparse regime? How do we construct mechanisms satisfying these privacy notions? Will the privatized network data set that is obtained by this mechanism preserve enough statistical utility? As pointed out in Narayanon and Shmatikov (2009), sparsity facilitates disclosure and at the same time it makes statistical inference more difficult. Therefore, on the one hand, we might need quite a stringent notion of privacy for sparse graphs, but on the other hand this requirement may drastically affect the statistical utility of the released network data set. As a consequence, a clear trade‐off between privacy guarantees and statistical utility arises, particularly in sparse settings. Natural questions are how to formalize this trade‐off mathematically and then how to solve it.

**C. Borgs and J. T. Chayes** (*Microsoft Research*)

This paper is an important contribution to statistical network modelling, giving a framework to formulate exchangeability for sparse graphs by embedding them in random measures on $R_{+}^{2}$ . It has already inspired several other works, including ours with H. Cohn and N. Holden (Borgs *et al*., 2016).

We put the Caron–Fox model into a broader context of *graphon processes* as follows. First, rather than viewing *w*_*i*_ as a ‘sociability’ parameter of node *i*, we think of it as a feature in some *σ*‐finite space Ω (e.g. **R** ^*d*^ with a locally finite measure, a discrete but not necessarily finite space, or a combination of the two). Second, rather than a product function, we consider an arbitrary two‐variable function (graphon) *W*:Ω×Ω→[0,1] over the feature space.

We construct a time‐dependent family (*G*_*t*_)_*t*⩾0_ of graphs by first generating a Poisson point process with intensity *tρ*, where *ρ* is an arbitrary *σ*‐finite measure on the feature space, then connecting two points with features *w*_*i*_ and *w*_*j*_ with probability *W*(*w*_*i*_,*w*_*j*_) and finally deleting points unconnected up to time *t*.

If we choose the feature space to be **R**_+_ and *W*(*w*,*w* ^′^)=1− exp (−2*ww* ^′^), we obtain the Caron–Fox model. If we take the space to be **R**_+_×[*k*], and *W* the product of the Caron–Fox graphon with a *k*×*k* matrix, we obtain the block model version of Herlau *et al*. (2016). Taking it to be **R**_+_ times the *k*‐simplex, we obtain a sparse, degree‐corrected mixed membership model, etc. Hence, our work represents a substantial extension of the modelling aspect of the authors’ work.

Our work also sheds light on the theoretical questions alluded to by the authors. First, it generalizes both their model and the standard, dense exchangeable model. Second, it completes the Caron–Fox picture by giving an *if and only if* characterization of exchangeability, whereas previous work (except for the simultaneous independent work of Veitch and Roy (2015)) gave only an *if* statement. Explicitly, labelling edges by the birth times of their end points, we obtain an exchangeable random measure on $R_{+}^{2}$ from an arbitrary graphon process. More significantly, we prove the *only if* statement: under mild decay conditions, all such measures can be obtained from graphon processes, extending the classic Aldous–Hoover theory for dense graphs to graphon processes.

**Alexandre Bouchard‐Côté and Creagh Briercliffe** (*University of British Columbia, Vancouver*)

First, we congratulate Caron and Fox for this impressive contribution, which is already starting to have significant influence in the field. In what follows, we discuss some computational aspects of posterior inference.

In the general case, the authors describe that the computational bottlenecks of posterior inference lie in updating the weights *w*_*i*_ and the latent counts ${\overset{¯}{n}}_{ij}$. Although there are more latent count variables than weight variables, sampling the former can be trivially parallelized, so, for sufficiently sparse graphs, we expect the weight updates to dominate.

The authors used Hamiltonian Monte Carlo within Gibbs sampling to perform the joint sampling of *w*_1:*Nα*_ given the rest of the variables, which seems a reasonable approach validated by solid experimental results. At the same time, given the prevalence of graph data of increasing size, we believe it is productive also to keep the door open to alternatives. In particular, the target log‐density has a simple structure in the generalized gamma process case, so it is tempting to ask whether this structure can be leveraged.

We investigate one potential direction to do so by using two variants of the bouncy particle sampler (BPS) algorithm (Bouchard‐Côté*et al*., 2017). Since the local variant of the BPS has been shown to outperform state of the art Hamiltonian Monte Carlo algorithms in sparse factor graphs, this appears *a priori* an appropriate tool for inference in a sparse network model. Surprisingly, however, the factor graph induced by sampling the *w*_*i*_s is in fact fully connected. We therefore resort to the global variant of the BPS algorithm.

We use the same reparameterization as the authors and compute collision times via superposition and adaptive thinning. We run the global BPS algorithm and Stan (Stan Development Team, 2016), each on a simulated graph of $N_{\alpha }=2558$ nodes, with the other parameters fixed to the values used to generate the data (w_*_=6.5427,*τ*=1 and *σ*=0.5). We observe that the latter outperformed the former (3.2 and 5.8 effective samples per second respectively). This is consistent with previous observations that BPS algorithms are most attractive when they can exploit sparsity (Bouchard‐Côté *et al*., 2017).

Perhaps sparsity of the inference problem might reappear in more complex models that build on the work of Caron and Fox. For example, it might be interesting to modify the follow‐up work of Todeschini and Caron (2016) to allow some community‐specific weights to be 0. Alternative constructions designed to accommodate covariates or node attributes might also potentially create additional sparsity structure.

**Trevor Campbell and Tamara Broderick** (*Massachusetts Institute of Technology, Cambridge*)

We congratulate Caron and Fox on their paper, which has already inspired further theory (Veitch and Roy, 2015; Borgs *et al*., 2016; Palla *et al*., 2016), an alternative class of models using independent and identically distributed data sampling of edges from a fixed random measure (Cai *et al*., 2016; Crane and Dempsey, 2015, 2016; Campbell *et al*., 2016a; Janson, 2017a), and models for dynamic networks (Palla *et al*., 2016), link prediction (Williamson, 2016), and block structure learning (Herlau *et al*., 2016).

The authors focus on the case where ‘vertex sociabilities’ are generated by the jumps of a Poisson process on $R_{+}$. They marginalize the latent Poisson process to devise an urn scheme and thereby a tractable Markov chain Monte Carlo sampler. However, in previous work, practitioners have often found it useful to represent the latent process explicitly: it is required by modern methods such as variational inference and Hamiltonian Monte Carlo sampling (Blei and Jordan, 2006; Neal, 2011); it can make inference simpler or tractable for hierarchical models; and it often facilitates parallel computing. The authors’ results, though, imply that there is a challenge to instantiating the Poisson process in the case of sparse graphs. In particular, they show that, if a graph sequence is sparse, the Poisson process must have a countable infinity of atoms in any restriction window [0,*α*] (theorem 2). But we cannot store infinitely many values in memory, or update infinitely many values in finite time, and therefore we must use an approximation.

One approximation involves replacing the full Poisson process with only finitely many jumps. The authors suggest weight‐based thresholding (Muliere and Tardella, 1998; Argiento *et al*., 2016) where jumps *x* ∈ [0,*ε*) are removed, but this requires dealing with non‐standard truncated probability distributions on $R_{+}$. We highlight another option: to *truncate a sequential representation* of the Poisson process (Campbell *et al*., 2016b). This technique has the advantage that it typically involves only well‐known exponential family distributions and thereby allows variational algorithms with simple closed form updates (Blei and Jordan, 2006; Doshi‐Velez *et al*., 2009). The approximation error of truncated sequential representations has recently been thoroughly characterized (Campbell *et al*., 2016b). However, these results do not immediately extend to the present network model, as it involves the product of a Poisson process with itself. Nonetheless, we conjecture that variational inference based on a truncated sequential representation would enjoy similar benefits for the network model as for previous applications in Bayesian non‐parametrics.

**Roberto Casarin** (*University Ca’ Foscari of Venice*), **Matteo Iacopini** (*University Ca’ Foscari of Venice and Université Paris 1—Panthéon‐Sorbonne*) **and Luca Rossini** (*University Ca’ Foscari of Venice and Free University of Bozen‐Bolzano*)

Caron and Fox are to be congratulated on their excellent research, which has culminated in the development of a new class of random‐graph models. The node degree and the degree distribution fail in giving a unique characterization of network complexity (Estrada, 2010). For this reason global connectivity measures, such as communicability (Estrada and Hatano, 2008, 2009) and centrality (Borgatti and Everett, 2006) are used to analyse a graph. In this discussion we contribute to the analysis of the generalized gamma process (GGP) model compared with the Erdoós‐Rényi and the preferential attachment (Barabási and Albert, 1999) models. Our analysis is far from being exhaustive but shows that more theoretical aspects of the GGP model are to be investigated.

A connected component of the *n*‐nodes graph *G*=(*V*,*E*) is a subgraph in which any two vertices *v*_*i*_ and *v*_*j*_ are connected by paths. The number of connected components equals the multiplicity of the null eigenvalue of the graph Laplacian *L*, where the (*i*,*j*) entry of *L* is

$$L_{ij}=\left\{\begin{array}{cc} d(v_{i}) & \;\text{if}\;i=j,\\ -1 & \;\text{if}\;i\neq j\;\text{and}\;(v_{i},v_{j})\in E,\\ 0 & \text{otherwise}, \end{array}\right.$$

with *d*(*v*_*i*_) the degree of *v*_*i*_.

The global clustering coefficient measures the tendency of nodes to cluster and is defined as

$$C=\frac{\text{number of triangle loops}}{\text{number of connected triples of vertices}}.$$

The assortativity coefficient between pairs of linked nodes is given by

$$r=\frac{\sum_{j=1}^{n}\sum_{k=1}^{n}jk\left(e_{jk}-q_{j}q_{k}\right)}{\sigma_{q}^{2}},$$

where *q*_*k*_ and *e*_*jk*_ are the distribution and the joint excess degree probability of the remaining degrees respectively, for the two vertices *v*_*j*_ and *v*_*k*_, and *σ*_*q*_ is the standard deviation of *q*_*k*_.

Finally, given the partition of the network into two non‐overlapping subgraphs (core and periphery) that maximizes the number or weight of within‐core‐group edges, we compute the share of nodes in the core.

**Figure 15:**
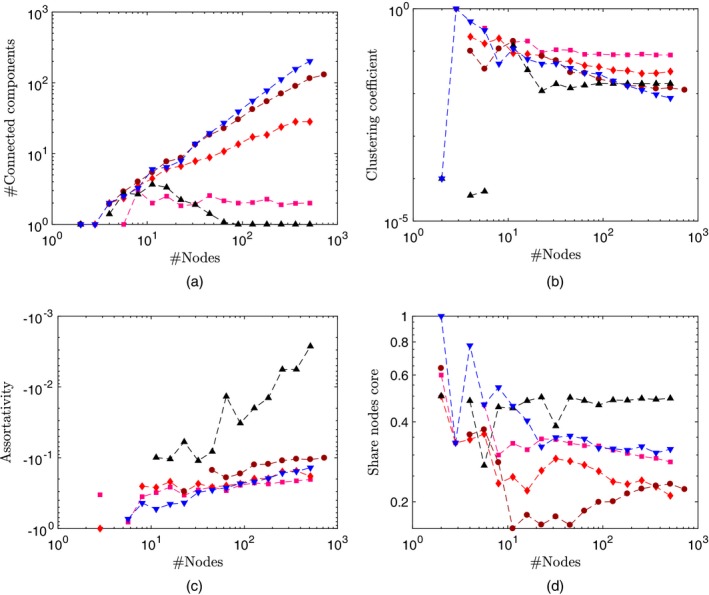
Netwok statistics *versus* number of nodes for the GGP undirected network (▪,σ=0;⧫,σ=0.5;∙,σ=0.8), the Erdoós–Rényi (▴) and the preferential attachment model of Barabási and Albert (1999) (▾): (a) number of connected components; (b) clustering coefficient; (c) assortativity coefficient; (d) share nodes core

According to Fig. 15 (a), the GGP couples with the preferential attachment model and performs slightly worse than the Erdoós–Rényi random graph in terms of the number of connected components. Figs 15(b) and 15(c) highlight that the clustering structure of GGP does not vary too much with *σ*. The clustering coefficient is in line with the two benchmarks while the assortativity of the Erdoós–Rényi model is not attained. For *σ*=0.5,0.8, the GGP exhibits a lower share of nodes in the core (Fig. 15(d)) than in the benchmarks and mimics the preferential attachment model for *σ*=0.

Overall, the GGP can replicate typical behaviours of real world sparse networks and some fundamental features of random graphs generated from the preferential attachment model, making it suitable for a variety of applications in different fields.

We are very pleased to thank the authors for their work.

**I. Castillo and T. Rebafka** (*Université Pierre et Marie Curie—Paris 6*)

The random‐graph model proposed by Caron and Fox has some remarkable properties, among others the possibility to combine sparsity and exchangeability and the scalability of computations within the associated Bayesian framework. In our view, their work raises some interesting questions on *statistical inference*. Adopting a frequentist approach, what can be said about posterior convergence assuming the data have been generated under a ‘true’ parameter?

Form a posterior distribution Π[·|*Z*] on $w\in {\left(R^{+}\right)}^{N^{+}}=:W$ from

$$Z|w∼\overset{n}{\underset{i=1}{⨂}}\text{Bernoulli}\left\{1-\exp\left(-2w_{i}w_{j}\right)\right\}=:P_{w},w∼\Pi .$$

Consider the behaviour of Π[·|*Z*] under two frequentist settings:

- *well specified*, where $Z∼P_{0}=P_{w_{0}}$ for an unknown fixed $w_{0}\in W$, and
- possibly *misspecified,* where $Z∼P_{0}=Q$ for an arbitrary graph distribution *Q*.

In a simulation study, we considered estimation of two simple functionals; the edge density and the density of triangles,

$$\psi_{1}=\frac{1}{\left(\begin{array}{c} n\\ 2 \end{array}\right)}E_{P_{0}}\left[\sum_{i<j}Z_{ij}\right]\;,$$

$$\psi_{2}=\frac{1}{\left(\begin{array}{c} n\\ 3 \end{array}\right)}E_{P_{0}}\left[\sum_{i<j<k}Z_{ij}Z_{jk}Z_{ki}\right]\;.$$

We used the default code under the generalized gamma process with improper priors on hyperparameters.

One reason to consider setting (b) is the specific exponential form of the link function considered in the paper, which may not hold for the data. Suppose for instance that *Z* has actually been generated from a stochastic block model with two groups, equiproportions and connectivity parameters

$$\alpha =\left(\begin{array}{cc} 0.8 & 0.1\\ 0.1 & 0.8 \end{array}\right)\;\;.$$

Simulations suggest that the posterior is consistent for *ψ*_1_, but inconsistent for *ψ*_2_.

**Figure 16:**
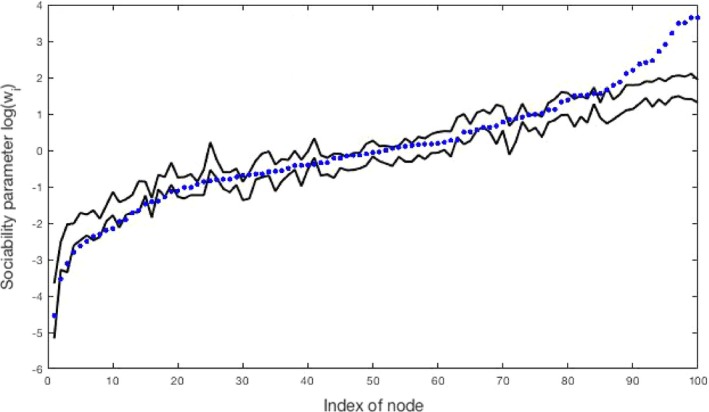
95% credible intervals (―) for log(*w*_*i*_) and true values (•) in a graph with 100 nodes (nodes are ordered by increasing values of log(*w*_*i*_))

In setting (a), we considered two cases. Case 1 is an equiproportions stochastic block model with connectivity matrix

$$\alpha =\left(\begin{array}{cc} 0.8 & \approx \frac{1}{3}\\ \approx \frac{1}{3} & 0.1 \end{array}\right)$$

compatible with the exponential link function. Bayesian and frequentist behaviours of *ψ*_1_ and *ψ*_2_ are remarkably close and rapidly converging (Table 3), suggesting that a Bernstein–von Mises theorem holds. Case 2 is a graph with ‘correctly specified’ link function and *w* a sample from the Cauchy distribution. The posterior still estimates the functionals *ψ*_1_ and *ψ*_2_ well but seems to underestimate large values of *w*_0,*i*_ (Fig. 16). To study sparsity, we repeated the two previous experiments but replacing $w_{0}$ by $\rho_{n}w_{0}$, where *ρ*_*n*_→0. We noted that the posterior on *σ* was concentrated on negative values, which suggests that *σ* may not universally quantify sparsity.

**Table 3:** Stochastic block model with equiproportions and compatible link function, true *ψ*_1_=0.3938 and *ψ*_2_=0.1026: mean lengths of 95% credible or confidence intervals and bias of posterior mean and frequentist estimates of *ψ*_1_ and *ψ*_2_ over 120, 120, 90 and 60 simulated graphs

*Number of*	*Credible*	^ψ1bayes	^ψ1freq	^ψ1freq	*Credible*	^ψ2bayes	^ψ2freq	^ψ2freq
*nodes n*	*interval*	*bias*	*interval*	*bias*	*interval*	*bias*	*interval*	*bias*
	*length*		*length*		*length*		*length*	
30	0.0794	−0.0025	0.0816	−0.0012	0.0502	−0.0058	0.0485	−0.0053
50	0.0470	−0.0011	0.0486	−0.0010	0.0302	−0.0043	0.0322	−0.0037
100	0.0234	0.0003	0.0234	0.0001	0.0153	−0.0018	0.0149	−0.0013
300	0.0078	−0.0003	0.0078	−0.0004	0.0051	−0.0007	0.0054	−0.0005

It would be interesting to determine which aspects of *w* (e.g. real‐valued functionals, or the complete vector *w*) can be estimated at minimax rate by using priors as in the paper. Another question would be to extend the model and priors to cover arbitrary link functions, and possibly more general forms of the law of *w*.

**Suman Chakraborty** (*University of North Carolina at Chapel Hill*)

The model proposed in this paper is aimed at providing a unified framework to incorporate desirable properties observed in real world networks such as exchangeability and sparsity. The model is defined for a graph for countably infinite vertices. A typical node *i* is codified via a function $\theta_{i}$ where *i* is a natural number and $\theta_{i}$ lies on the non‐negative half of the real line and can be seen as the time that node *i* entered the network. Finally the network is represented as the point process

$$Z=\sum_{ij}z_{ij}\delta_{\left(\theta_{i},\;\theta_{j}\right)},$$

where *z*_*ij*_=1 indicates that there is an edge between nodes *i* and *j*. Interestingly Kallenberg's exchangeable completely random measures theory is devised on the appearance time of the nodes; formally the following distributional equality holds:

$$Z\left(A_{i}\times A_{j}\right)=Z\left(A_{\pi (i)}\times A_{\tilde{\pi }\left(j\right)}\right),$$

for all natural numbers *i* and *j*,* π* and $\tilde{\pi }$ are arbitrary permutations of natural numbers $(\pi =\tilde{\pi }$ for joint exchangeability) and *A*_*i*_=[*h*(*i*−1),*hi*] for any *h*>0. Finally to introduce node inhomogeneity a set of parameters {*w*_*i*_} is introduced and particular link probabilities are assumed.

It seems that theorems 2 and 3 are the main advantage of this modelling approach. Roughly it enables us to produce graphs with the desired level of sparsity by tuning a single parameter. This property can be appealing for applications. Also the sparsity parameter might be useful to obtain scalings of important functions of the graph, such as the number of triangles. The projectivity is also a salient feature of the model as it can be used to draw inference on the unobserved part of the population. It would be interesting to analyse how much information is necessary to make valid inference about the whole network.

The form of exchangeability of the model is cleverly devised and enabled the authors to use known representations of Kallenberg (1990), which is a crucial component of the paper. It will be helpful for the reader to have a few more comments from the authors on the statistical interpretations and scope of the notion of exchangeability used here. Finally as suggested by Fig. 8(a) the power‐law‐like tail behaviour of the empirical degree distribution, the projectivity and tunable sparsity parameter make the model attractive to both theoretical and practical applications.

**Harry Crane** (*Rutgers University, Piscataway*)

Caron and Fox tout their proposal as

‘the first fully *generative* and *projective* approach to *sparse* graph modelling [  …] with a notion of *exchangeability* that is essential for devising our scalable statistical estimation procedure’

(at the end of Section 4; emphasis added). In calling theirs the *first* such approach, they brush aside prior work of Barabási and Albert (1999), whose model is also generative and projective, and produces sparse graphs. The Barabási–Albert model is not exchangeable, but nor is the authors’. And, although the Barabási–Albert model is inadequate for most statistical purposes, the model proposed is not obviously superior, especially with respect to the criteria highlighted above.

*Generative*

Though amenable to simulation, the obscure connection between Kallenberg's theory of exchangeable completely random measures and the manner in which real networks form makes it difficult to glean much practical insight from this model. At least Barabási and Albert's preferential attachment mechanism offers a clear explanation for how sparsity and power law might arise in nature. I elicit no such clarity from the Caron–Fox model.

*Projective*

Proiectivity is important for relating observed network to unobserved population and is therefore crucial in applications for which inferences extend beyond the sample. Without a credible sampling interpretation, however, the statistical salience of projectivity is moot. Here projectivity involves restricting point processes in $R_{+}^{2}$ to bounded rectangles [0,*α*]^2^, whose best‐known interpretation via *p*‐sampling (Veitch and Roy, 2016) seems unnatural for most conceivable networks applications, including those in Section 8.

*Exchangeability and sparsity*

A certain nonchalance about whether and how this model actually *models* real networks betrays an attitude that sees sparsity as an end in itself and exchangeability as a means to that end. Even the authors acknowledge that ‘exchangeability of the point process [  …] does not imply exchangeability of the associated adjacency matrix’ (on the third page). So why is there all the fuss about exchangeability if its primary role here is purely utilitarian? To me, the pervasiveness of ‘exchangeability’ throughout the paper is but a head fake for unsuspecting statisticians who, unlike many modern machine learners, understand that exchangeability is far more than a computational contrivance.

*Final comment*

The Society's Research Section is all too familiar with the Crane–Dempsey edge exchangeable framework, which meets the above four criteria while staying true to its intended application of interaction networks. For brevity I refer the reader to Crane and Dempsey (2015, 2016) for further discussion.

**Daniele Durante** (*University of Padova*)

There is increasing interest in modelling network data under a Bayesian non‐parametric perspective, and the paper from Caron and Fox provides an appealing contribution in this direction. In fact, as they effectively demonstrate, many Bayesian non‐parametric representations not explicitly motivated by network data can indeed provide powerful methods for studying relevant patterns underlying real world connectivity structures.

Consistent with this focus, the growing demand for more flexible and realistic statistical models for networks is a main motivation underlying the stochastic point process representation of Caron and Fox, implying less restrictive exchangeability assumptions. Their model is carefully parameterized to provide flexible inference on sparsity and the degree distribution, which represent the quantities of main interest. However, a natural question when combining Bayesian non‐parametric representations and complex data is whether the resulting Bayesian formulation induces large support priors for a wider set of expected network features—e.g. clustering coefficient and average shortest path length. These are relevant measures in characterizing other realistic properties, such as small world architectures, and an improved characterization of prior support may provide insights on which properties the model can flexibly incorporate.

Looking at this problem from a broader perspective, Caron and Fox consider settings in which a single network is observed. In such cases the information is clearly not sufficient to learn highly flexible representations, and although restricting the focus to a specific class of distributions—by incorporating reasonable structure—is a clever approach, it does not protect from misspecification issues. For instance the novel exchangeability assumption proposed by the authors effectively improves flexibility in characterizing sparsity but could substantially restrict the support on other relevant classes of distributions. Therefore, looking at the model from an applied perspective, it is fundamental to understand the extent to which their Bayesian non‐parametric formulation can be viewed as truly non‐parametric. Indeed, without prior support about the true distribution of the random graph, the posterior can never concentrate around the truth, thereby affecting quality of inference.

A possible answer to this issue is to generalize the model from Caron and Fox to obtain a fully flexible characterization of the entire distribution for random graphs. Motivated by the availability of replicated networks, Durante *et al*. (2017) addressed this goal with a focus on latent space models (e.g. Hoff *et al*. (2002)), and I am excited to see how the inspiring contribution from Caron and Fox can open new avenues also in this direction.

**Chao Gao** (*University of Chicago*)

I congratulate Caron and Fox for their results that give a solid foundation for exchangeable network analysis. This paper has already inspired some important subsequent works (Veitch and Ray, 2015; Borgs *et al*., 2016; Crane and Dempsey, 2016; Broderick and Cai, 2015). My discussion will focus on the potential influence on the development of methods and algorithms under this new framework.

*Within‐sample inference*

Given a network of finite size *D*_*α*_, the locations are labelled *θ*_[1:*n*]_=(*θ*_1_,…,*θ*_*n*_), with *n*∼ Poisson(*α*), Consider the problem of estimating *w*_[1:*n*]_=(*w*_1_,…,*w*_*n*_) in a frequentist way. The conditional likelihood function $p({\left\{n_{ij}\right\}}_{1⩽i,\;j⩽n}|\theta_{\left[1:n\right]},w_{\left[1:n\right]},n)$ is

$$\prod_{1⩽i\neq j⩽n}\frac{{\left(w_{i}w_{j}\right)}^{n_{ij}}}{n_{ij}!}\exp\left(-w_{i}w_{j}\right)\times \prod_{1⩽i⩽n}\frac{w_{i}^{2n_{ii}}}{n_{ii}!}\exp\left(-w_{i}^{2}\right).$$

This is the same likelihood function as in Karrer and Newman (2011) under the node exchangeable framework (or its non‐exchangeable sparse version with a scaling parameter (Bickel and Chen, 2009). Another important problem is community detection. An analogous block model was proposed by Herlau *et al*. (2016) under the framework of exchangeable random measures. Again, given (*θ*_[1:*n*]_,*w*_[1:*n*]_,*n*), the conditional likelihood of Herlau *et al*. (2016) is the same as that of Karrer and Newman (2011) for degree‐corrected block models. The two examples show that, for within‐sample inference, existing methods, algorithms and even theorems in the framework of Bickel and Chen (2009) can generally be applied to the new framework of Caron and Fox without change, at least from a frequentist perspective.

*Out‐of‐sample inference*

Given the discussion in the previous paragraph, I believe that the development of new methods and algorithms should be emphasized on out‐of‐sample inference. The exchangeability framework of Caron and Fox makes rigorous inference on the population or future observations possible for the first time in sparse networks, for which they should be congratulated again. After all, the models of Caron and Fox and later extensions (Herlau *et al*., 2016; Todeschini and Caron, 2016) are defined on the entire population, whereas the degree‐corrected block model (Karrer and Newman, 2011) is defined only on a finite set of nodes.

*Subgraph densities*

Recently, it has been shown by Gao and Lafferty (2017) that statistics based on small subgraph frequencies can lead to optimal testing procedures between Erdős–Rényi models and stochastic block models. Empirical studies also illustrate that structures of social networks can be reflected by statistics of small subgraph frequencies (Ugander *et al*., 2013). It is not very clear to me how analogous ideas can be developed in the framework of exchangeable random measures. For example, let *T* be the total number of triangles in a given network *Z*_*α*_. To define the empirical triangle frequency, should *T* be normalized by $\left(\begin{array}{c} {}^{n}\\ {}_{3} \end{array}\right)$, by $\left(\begin{array}{c} {}^{N_{\alpha }}\\ {}_{3} \end{array}\right)$ or by *α* ^3^? What is a proper definition of the population triangle density? Inference for population subgraph densities are out of sample and thus deserves future investigations under the framework of Caron and Fox.

**Jim E. Griffin and Fabrizio Leisen** (*University of Kent, Canterbury*)

We congratulate Caron and Fox for this stimulating paper which proposes a new perspective on network models. The use of completely random measures (CRMs) provides a tractable and rich framework for modelling graphs. CRMs have been intensively studied in Bayesian non‐parametrics and this provides a ready‐made toolkit for the development of novel and tractable network models. One example is the development of vectors of CRMs; see Leisen and Lijoi (2011), Griffin *et al*. (2013), Griffin and Leisen (2017) and the references therein. Section 3.2 of the paper illustrates the use of the methodology for modelling directed multigraphs. We believe that vectors of CRMs can be employed in this setting for modelling multiple types of interactions (such as ‘message’, ‘SMS’, ‘like’ and ‘tag’ as in the paper). This can be achieved by considering different but related multigraphs for each interaction. In the spirit of compound random measures (Griffin and Leisen, 2017), we model the *k*th interaction (from *p* possible interactions) as

$$D_{k}=\sum_{i=1}^{\infty }\sum_{j=1}^{\infty }n_{ijk}\delta_{\left(\theta_{i},\;\theta_{j}\right)}$$

where

$$D_{k}|W_{1},\ldots ,W_{p}∼\text{PP}\left(W_{k}\times W_{k}\right)W_{k}=\sum_{i=1}^{\infty }w_{i}m_{ki}\delta_{\theta_{i}}.$$

The *w*_*i*_s and the *m*_*ki*_s are drawn from the *directing Lévy measure*

$$W=\sum_{i=1}^{\infty }w_{i}\delta_{\theta_{i}}∼\text{CRM}\left(\rho ,\lambda \right),$$

and the *score* distribution *h* respectively. Extending the interpretation in the paper, the *w*_*i*_s represent an overall sociability and the *m*_*ki*_s adjust for differences in the levels of different types of interaction. This approach has been employed by Todeschini and Caron (2016) for modelling networks with overlapping communities. An attractive feature of this framework is that the parametric score distribution *h* can easily be extended, in standard ways, to include covariates or time variation.

We believe that this paper will become a cornerstone of network modelling as well as a natural application of Bayesian non‐parametric techniques.

**Lancelot F. James** (*Hong Kong University of Science and Technology*) **and Creighton K. Heaukulani** (*Hong Kong*)

We congratulate Caron and Fox on a paper that has generated considerable interest. This work has influenced our thought process on some projects we are working on (Bloem‐Reddy *et al*., 2017), and this discussion reflects some of those elements. We ask one question and offer some comments. Generalizing Section 5.5 (see equation (31)), we can construct processes of the form, for *q*=1,2,3,…

$$D_{\alpha ,\;q}\overset{d}{=}\sum_{k=1}^{\infty }b_{k}\delta_{\left(w_{k,\;1},\;\ldots ,\;w_{k,\;\;q}\right)}\overset{d}{=}\sum_{k=1}^{D_{\alpha ,\;q}^{*}}\delta_{\left(U_{k,\;1},\;\ldots ,\;U_{k,\;\;q}\right)}$$

where $\left(b_{k}\right)|\left(p_{k}\right){∼}^{\mathrm{ind}}\text{Bernoulli}\left(p_{k}\right)$, and (*p*_*k*_) are points of a Poisson process with intensity *τ*, satisfying $\int_{0}^{1}v\tau \left(v\right)dv=1$. This implies that $D_{\alpha ,\;q}^{*}|W_{\alpha }^{*}$ is $\text{Poisson}(W_{\alpha }^{*q})$. Hence, for *q*=1, we might associate *D*_*α*,1_ with a hierarchical Indian buffet process, and *D*_*α*,2_ equates with the marginal structure considered by the authors. In this regard, we refer to these processes as *hierarchical q‐latent feature models*. A question to the authors is how might we interpret *D*_*α*,*q*_ for *q*⩾3? Now, by the Bayes rule, there is a conditional density of $W_{\alpha }^{*}|D_{\alpha ,\;q}^{*}=l$, given by

(79) $$f_{\alpha }^{\left(l,\;q\right)}\left(t\right):=\frac{t^{ql}\exp\left(-t^{q}\right)f_{W_{\alpha }^{*}}\left(t\right)}{E[W_{\alpha }^{*ql}\exp\left(-W_{\alpha }^{*q}\right)]}.$$

Then, $F_{\alpha }\left(y\right):=W_{\alpha }\left(y\right)/W_{\alpha }^{*}:=\Sigma_{k=1}^{\infty }Q_{k}I_{\left\{V_{k,\;\alpha }⩽y\right\}}$ given $D_{\alpha ,\;q}^{*}=l$ is such that $\left(Q_{k}\right)|D_{\alpha ,\;q}^{*}=l$ has a mixed Poisson–Kingman distribution denoted $\text{PK}\left(\rho ,f_{\alpha }^{\left(l,\;q\right)}\right)=\int_{0}^{\infty }\text{PK}\left(\rho |t\right)f_{\alpha }^{\left(l,\;q\right)}\left(t\right)dt$ (see Pitman (2003)). When *l*>0, the density (0.1) also corresponds to that of $W_{\alpha }^{*}|G_{l}^{1/q}/W_{\alpha }^{*}=1$, where *G*_*l*_ is a gamma(*l*,1) variable. So, for *q*=2, another contribution of the paper of Caron and Fox is to identify classes of unexplored Poisson–Kingman distributions that naturally appear in an interesting context. The appearance of $\text{PK}(\rho ,f_{\alpha }^{\left(l,\;2\right)})$ is a little more obvious in an earlier draft of the authors’ work. In general, one samples $U_{1}^{\prime },\ldots ,U_{ql}^{\prime }|F_{\alpha },D_{\alpha ,\;q}^{*}=l{∼}^{\mathrm{IID}}F_{\alpha }$, where $F_{\alpha }|D_{\alpha ,\;q}^{*}=l∼\text{PK}\left(\rho ,f_{\alpha }^{\left(l,\;q\right)}\right)$. A subtle point about this sampling scheme, inherent to latent feature models, is that, unlike the Bayesian non‐parametric statistics latent class setting where one samples *n* points from *F*_*α*_, different from *ql*, the sample size *ql*, dictated by $D_{\alpha ,\;q}^{*}=l$, always agrees with the superscripts in $\text{PK}(\rho ,f_{\alpha }^{\left(l,\;q\right)})$. This explains the cancellations in equation (71). See James *et al*. (2009) (and also James (2002)), which exhibits all the pertinent structures for *q*=1.

**Svante Janson** (*Uppsala University*)

I find the random‐graph model introduced here by Caron and Fox very interesting. Apart from its potential use in applications, it has novel and interesting mathematical properties. Moreover, it has been an inspiration of important generalizations developed after the first version of the present paper by, in particular, Borgs *et al*. (2016) and Veitch and Roy (2015).

The relationship with Kallenberg's characterization of exchangeable random measures is interesting, and presumably useful in further developments of the theory, but I would like to stress that, for the content of the present paper, Kallenberg's highly technical theorem may serve as a (possibly important) inspiration for the model, but it is not needed for the formal construction of the model and the study of its properties.

Furthermore, the basic construction can be stated in several, equivalent, ways. I prefer to see the basic construction in the paper as follows, including generalizations by Borgs *et al*. (2016) and Veitch and Roy (2015). Let (S,μ) be a *σ*‐finite measure space, and let *F*(*x*,*y*) be a fixed symmetric measurable function S×S→[0,1]. Generate a random countable point set ${\left(w_{i},\theta_{i}\right)}_{1}^{\infty }$ of points in $S\times R_{+}$ by taking a Poisson point process in $S\times R_{+}$ with intensity *μ*×*λ*. Regard the $\theta_{i}$ as (labels of) vertices, and add an edge $\theta_{i}\theta_{j}$ with probability *F*(*w*_*i*_,*w*_*j*_), independently for all pairs $\left(\theta_{i},\theta_{j}\right)$ with *i*⩽*j*. (Finally, eliminate isolated vertices.) The version in the present paper constructs ${\left(w_{i},\theta_{i}\right)}_{1}^{\infty }$ by a completely random measure, which is equivalent to choosing $S=R_{+}$ with *μ* the Lévy measure; furthermore, *F* is chosen as *F*(*x*,*y*)=1− exp (−2*xy*) (for *x*≠*y*). Kallenberg's theorem yields the same random graphs by a canonical choice $\left(S,\mu \right)=\left(R_{+},\lambda \right)$, but a different *F*; see Section 5.1. Other choices of *F* yield generalizations of the model. Other choices of (S,μ) yield the same random graphs but are sometimes useful, so it seems convenient to allow an arbitrary choice and not to fix it in advance.

Finally, in connection with theorems 3 and 5, note that, if $\int_{0}^{\infty }w\rho \left(dw\right)<\infty$, then $N_{\alpha }^{\left(e\right)}/E\left[N_{\alpha }^{\left(e\right)}\right]\to 1$ almost surely as *α*→∞. This follows easily because the loops can be ignored and, if ${\overset{¯}{N}}_{\alpha }^{\left(e\right)}$ denotes the number of non‐loop edges and the edges are defined by the events *U*_*ij*_⩽*F*(*w*_*i*_,*w*_*j*_) for an independent and identically distributed array (*U*_*ij*_)_*i*⩽*j*_, then ${\overset{¯}{N}}_{\alpha }^{\left(e\right)}/\alpha^{2}$ is a reverse martingale with respect to the *σ*‐fields $F_{t}$ generated by ${\left(w_{i}\right)}_{1}^{\infty }\cup {\left(U_{ij}\right)}_{ij}\cup {\left(\theta_{i}1_{\theta_{i}>t}\right)}_{1}^{\infty }$.

**Kuldeep Kumar** (*Bond University, Gold Coast*)

One of the important contributions of this paper is the development of a scalable algorithm which enables inference of the graph parameters determining the sparsity of the graph. For visual graph display the graphs can be turned from dense to sparse based on a single parameter for a specific choice of completely random measure. One of the problems which is inherent in these kinds of algorithms is the choice of parameters (*α*,*δ*,*γ*). There are many developments in the area of machine learning algorithms specifically in the area of tree decomposition like random forests and stochastic gradient boosting (treenet). Have Caron and Fox compared this algorithm which relies on tree decomposition or the pebble game algorithm as developed by Lee and Streinu (2008)?

**Antonio Lijoi** (*Bocconi University, Milan*), **Ramsés H. Mena** (*Universidad Nacional Autónoma de México, Mexico City*) **and Igor Prünster** (*Bocconi University, Milan*)

We congratulate Caron and Fox for proposing a clever construction of random graphs for networks, which enables us to achieve sparsity in an effective way. This represents another successful instance of Bayesian non‐parametric modelling based on completely random measures (CRMs).

Among several potential developments, of particular interest is the extension to a multisample context with data recorded from two or more networks. Assuming that the data are still conditionally independent but not identically distributed, a natural problem is the derivation of testing procedures to verify whether the probability distributions, or some of their features, are shared across samples. In the generalized gamma process (GGP) case, the parameter *σ* plays a pivotal role: it not only controls the sparsity of the graph, but it also influences posterior clustering of the data. See Lijoi *et al*. (2007). Hence, homogeneity between two networks, directed by independent GGPs, may be assessed by testing for equality of their respective *σ*‐parameters. Along these lines, Lijoi *et al*. (2008) defined a Bayesian test on the discount parameter of the Pitman–Yor process, which plays the same role as *σ* for the GGP.

Alternatively, network comparisons can be faced by assuming a richer model with dependent CRMs (*W*_1,*α*_,*W*_2,*α*_) accommodating a wide spectrum of dependence structures across networks, ranging from exchangeability (i.e. distributional homogeneity) to unconditional independence. In this framework, we may test whether the two distributions themselves are equal. Recently, unrelated to network applications, Bacallado *et al*. (2015) have addressed the issue within a parametric model and provided an insightful discussion on the notion of approximate exchangeability. A natural model that may serve the purpose is based on ideas in Rodríguez *et al*. (2008), where the nested Dirichlet process is introduced for clustering probability curves. Similarly, this approach may be useful for clustering networks on the basis of distributional similarity. Here, however, we need a model that can handle CRMs and a potentially fruitful approach is proposed in Camerlenghi (2015). Let $\tilde{q}$ be a discrete random probability measure on the space of boundedly finite measures $M_{R_{+}}$ on $R_{+}$, and *q*_0_ is the probability distribution of a CRM on $R_{+}$. If $\left(W_{1}^{*},W_{2}^{*},W_{0}^{*}\right)|\tilde{q}∼{\tilde{q}}^{2}\times q_{0}$, define $\left(W_{1,\;\alpha },W_{2,\;\alpha }\right)=\left(W_{1}^{*}+W_{0}^{*},W_{2}^{*}+W_{0}^{*}\right)$. Discreteness of $\tilde{q}$ implies that with positive probability $W_{1}^{*}=W_{2}^{*}$, which in turn yields *W*_1_=*W*_2_. This corresponds to similarity of the networks as they have the same distribution.

**Jorge Mateu** (*University Jaume I, Castellón*) **and Matthias Eckardt** (*Humboldt‐Universität zu Berlin*)

Caron and Fox are to be congratulated on a valuable contribution and thought‐provoking paper on sparse random graphs which examines the exchangeability for a continuous space representation of networks. In particular, they propose a new framework to study random‐graph models based on the notion of modelling the graph as an exchangeable random measure, and appealing to a Kallenberg representation for such exchangeable random measures in connection with planar point processes. For a regularly shaped square lattice, the authors define a point process to be exchangeable if the counted number of edges per grid site is exchangeable for any arbitrary square lattice. This is a very timely topic bridging random‐graph models to the field of spatial point processes. Our discussion focuses on the linkage of random‐graph structures to the spatial domain.

For spatial point processes, we consider a realization of a random sequence of locations on a complete separable metric space equipped with a suitable Borel *σ*‐algebra such that the cardinality of locations that fell in a given area contained in the observation window can be expressed by means of a random measure. This counting measure can refer to a purely spatial point process or, more sophisticated, to a marked spatial point process, e.g. a multivariate spatial point pattern. For such data, one is interested in the structural exploration and the detection or extraction of the characteristics and features within and between distinct sets of events. Although a large body of literature on the analysis of spatial point patterns exists, applications of random‐graph models still remain very limited. One example of such random graphs for spatial point patterns are neighbouring networks which include random structures in the calculation of planar point pattern characteristics (see, for example, Marchette (2004)). See also some recent approaches in Eckardt and Mateu (2017a, b, c).

We note that the concept of exchangeability is quite close to quadrat counting and the definition of complete spatial randomness in point processes. The authors comment on counting the number of edges per grid site, whether they are directed or not (see Section 3.2). We have several versions of counting or sampling in point processes that do have a clear counterpart in the corresponding edge counting process in graphs. Network data are becoming increasingly available and connections between graph models and planar point processes are a welcome line of research, together with extensions to spatiotemporal planar processes.

**Matthew Moores and David Firth** (*University of Warwick, Coventry, and Alan Turing Institute, London*)

Caron and Fox have achieved a major breakthrough in computationally tractable inference for random graphs with hundreds of thousands of nodes. This will enable simulation and Bayesian analysis of data sets that were previously infeasible. The scalability of their approach is due to the representation of the discrete graph structure using a latent continuous model, the generalized gamma process. The sociability parameters *w*_*i*_ of each node can be estimated by using Hamiltonian Monte Carlo sampling, since the gradient of the conditional log‐posterior is available in closed form. Further improvements in scalability might be achieved by taking advantage of parallelism, as well as by rewriting portions of the code in a compiled language.

**Figure 17:**
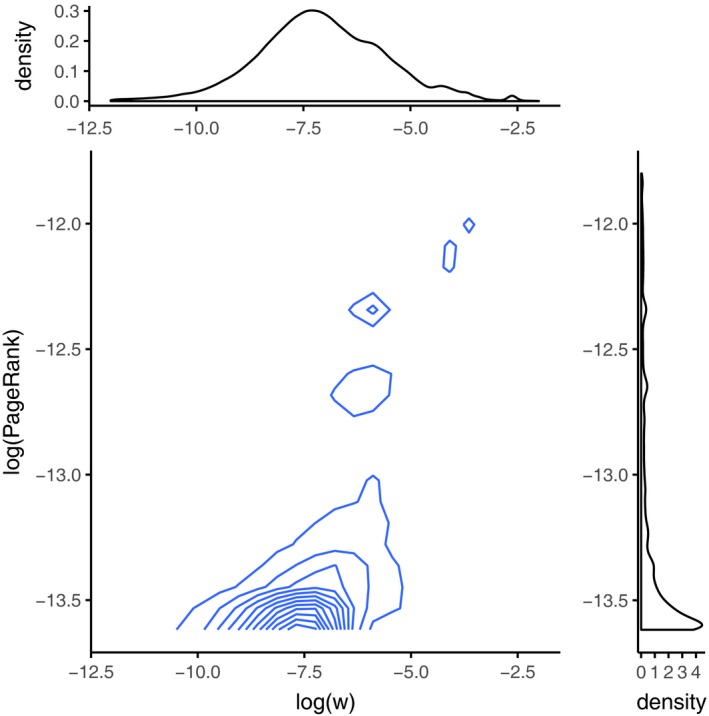
Logarithmic contour plot of PageRank against the sociability parameters *w*_*i*_ for the World Wide Web data set, with marginal densities

The pair potentials $\text{Pr}\left(z_{i,\;j}=1|w_{i},w_{j}\right)=\text{Pr}\left(n_{i,\;j}+n_{j,\;i}>0|w_{i},w_{j}\right)$ assume independence between in‐degree and out‐degree, even in the case of directed multigraphs. This implies a very different generative process from models of adversarial networks, where nodes compete with each other for edges. Examples include the Bradley–Terry model of citation networks (Varin *et al*., 2016) or the Plackett–Luce model of ranking data (Gormley and Murphy, 2006). A non‐parametric Plackett–Luce model has recently been proposed by Caron *et al*. (2014). The contour plot in Fig. 17 compares the distribution of *w*_*i*_ for the World Wide Web data set (Albert *et al*., 1999) with the PageRank of each node (Brin and Page, 1998). PageRank was computed by using the igraph package (Csárdi and Nepusz, 2006; Kolaczyk and Csárdi, 2017) for R (R Core Team, 2017). The difference in emphasis between these two approaches might explain why the tail of the degree distribution was underestimated for this data set, due to violation of the model assumptions. Nevertheless, the authors have demonstrated close agreement between the posterior predictive and the empirical degree distributions of a variety of real world graphs, both dense and sparse.

A curious aspect of the generalized gamma process is the parameter $w_{*}=W_{\alpha }^{*}-\Sigma_{i=1}^{N^{\alpha }}w_{i}$, which represents the left‐over sociability once all of the nodes with degree 1 or greater have been accounted for. This parameter is only partially identified, since it conflates degree 0 nodes with the potentially infinite number of nodes that might enter the network at a future time. Nodes with degree 0 do not contribute to the likelihood in any way, but it seems feasible to sample sociability parameters for these nodes from the prior, as with finite network models (Caron and Doucet, 2012). The parameter w_*_ could be viewed as an upper bound on the sociability of these nodes, particularly in the case where the network is assumed to be a closed system.

**Yang Ni and Peter Müller** (*University of Texas at Austin*)

We congratulate Caron and Fox on a very interesting paper. Our discussion highlights a particular use of the proposed models that we felt was missing in the paper. Implicit in the paper is an assumption that (part of) the random graph *D* (or a derived undirected graph *Z*) is observable. Although this is common for social network data, it is less common in biomedical inference where the goal is often to infer an unknown latent network structure.

The typical inference is set up under a hierarchical model

$$y_{i}∼p\left(y_{i}|\beta \right),\beta ∼p\left(\beta |D\right),p\left(D|\phi \right),\phi ∼p\left(\phi \right),$$

where *p*(*β*|*D*) maps the graph to the parameters *β* (Caron and Fox have already used up all other Greek letters) of the top level sampling model for the observed data **y**. This could be, for example, a Gaussian graphical model for protein activation **y**_*i*_. And we discuss another example below. We suggest the use of the proposed novel models for *p*(*D*|*ϕ*). Good prior regularization is more important in this context than in applications where the network is observed.

We illustrate our suggestion with a small simulation study and an application. Both are based on directed cyclical graphs (Ni *et al*., 2016), a special case of reciprocal graphical models (Koster, 1996). The directed cyclic graph allows inference on a directed graph *G*, possibly including cycles, by setting up a simultaneous equation model and interpreting a directed edge (*l*,*i*) in the graph *G* as an indicator for a non‐zero coefficient of *y*_*l*_ in the equation for *y*_*i*_. In this context we explore the use of a generalized gamma process (GGP) prior *p*(*D*|*ϕ*), including a mapping of a multigraph *D* to a directed graph *G* by mapping $n_{ij}\mapsto I\left(n_{ij}>0\right)$.

**Figure 18:**
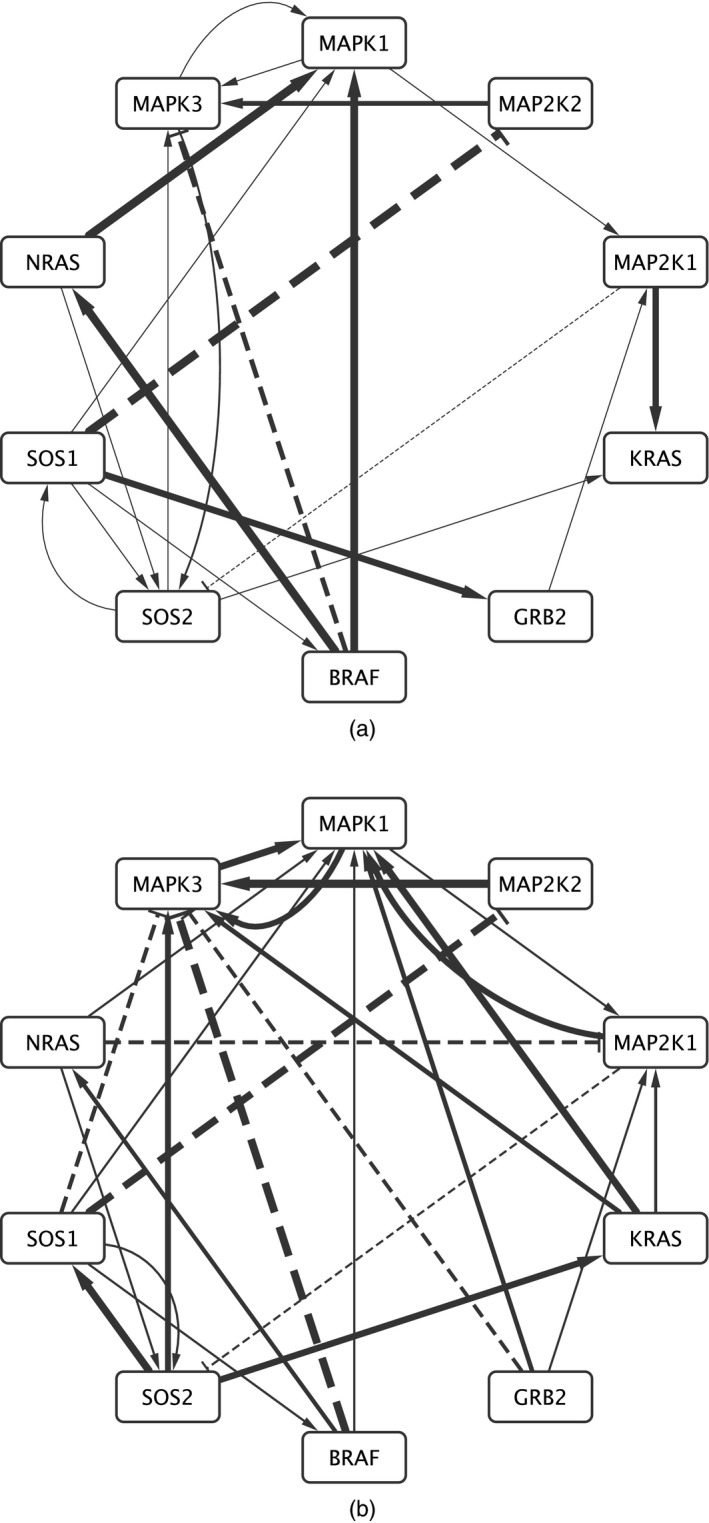
Estimated gene networks for TCGA colon cancer data under (a) the GGP prior and (b) the thresholded prior (Ni *et al*., 2016): the posterior expected false discovery rate is controlled to be less than 10% for both estimations

Table 4 reports summaries for a simulation study with four alternative priors *p*(*D*|*ϕ*). The GGP wins. Fig. 18 shows the estimated graphs in inference for a gene network reported in Ni *et al*. (2016) under the sparsity prior used there (thresholded prior) *versus* the new GGP prior. Also under the realistic conditions of this data analysis the choice of prior matters. Importantly, implementation of posterior inference was quite straightforward, as described in the paper.

**Table 4:** Simulation study: true positive rate TPR and false discovery rate FDR under four prior models—Erdős–Rényi graph with *p*=0.5, ER; Erdős–Rényi graph with *p*∼Be(0.5,0.5), ER+beta; GGP; thresholded prior, TP

	*ER(* $\frac{1}{2}$ *)*	*ER*+*beta*	*GGP*	*TP*
TPR	1.00	1.00	0.87	0.87
FDR	0.58	0.72	0.07	0.18

**Peter Orbanz** (*Columbia University, New York*)

Caron and Fox have set out to address misspecification problems of graphon models, described in Orbanz and Roy (2015). Are they solved? Exchangeable graphs are dense and ‘amorphous’ collections of edges. Caron and Fox's non‐compact modification makes them sparse, but still amorphous, and I would hence argue it addresses one symptom of a deeper problem. Nonetheless, a complete solution would be much to ask for, and I believe that Caron and Fox have made a very valuable contribution.

The vague notion that these graphs lack structure can be made more precise (Orbanz, 2017). Start with a fixed, finite graph *g*_*k*_ of size *k*, invent a randomized algorithm that generates a random subgraph *S*_*n*_(*k*) of size *n* and take the distributional limit *S*_*n*_ in input size *k*→∞. If the algorithm selects vertices independently and reports the induced subgraph, *S*_*n*_ is exchangeable, and the resulting random graphs are those represented by graphons. More generally, consider a randomized subsampling algorithm, and ask what set of transformations leaves the output distribution invariant. This relates the distributional invariance to experimental design for networks (e.g. Kolaczyk (2009)). Invariance, in turn, explains inference from a single realization: if only one network is observed, and modelled as a random graph, it constitutes a sample of size 1. Inference is hence generally ill posed, but invariance can explain averaging *within* a single graph. One can obtain laws of large numbers (Orbanz, 2017). Roughly: if the subsampling algorithm generates output invariant under a nice transformation group **G**, there is an enumeration *ϕ*_1_,*ϕ*_2_,… of the elements of **G** such that

$$\frac{1}{n}\sum_{i=1}^{n}f\left\{\phi_{i}\left(S_{n}\right)\right\}\overset{n\to \infty }{⟶}E\left[f\left(S_{\infty }\right)|\text{input graph}\right]\;\;\;\text{almost surely}$$

for any **L**_1_‐function *f*.

From this perspective, misspecification of exchangeable graphs is a consequence of selecting vertices independently of the edge set. To obtain more structure, sampling can ‘follow edges’ (e.g. extract neighbourhoods), but finding concisely represented models then becomes much harder. The subsampling implicit in Caron and Fox's model is site percolation (Veitch and Roy, 2016): each vertex is selected or deselected independently with fixed probability, which can yield sparsity, but still selects vertices ignoring edges.

If it remains to be seen how widely applicable the proposed models are in actual network analysis problems, I see the merit of this work elsewhere: Veitch and Roy (2016) is a part of a beautiful body of work (Veitch and Roy, 2015; Borgs *et al*., 2016; Janson, 2016) expanding Caron and Fox's non‐compact modification into a fully fledged generalization of graphon theory. Caron and Fox have introduced an idea that has enriched the landscape of network theory, and I wholeheartedly congratulate them.

**Konstantina Palla and Xenia Miscouridou** (*University of Oxford*)

We congratulate Caron and Fox for this excellent work on sparse graphs. It is of great interest to the whole community in statistical machine learning (and beyond)!

The authors have proposed a novel statistical model for networks that builds on exchangeable random measures. Their construction accounts for sparsity; an extremely important property in real world scenarios. In a graph context, a graph is defined as dense if the number of edges grows quadratically with the number of nodes and sparse if it scales subquadratically. Then, in the sparse regime, two nodes chosen at random are unlikely to be connected. Whereas many real world networks are believed to be sparse (Newman, 2009) most of the popular Bayesian models used in network analysis account for dense graphs, i.e. the number of edges grows quadratically in the number of nodes. The fundamental reason for this misspecification is the classical representation of the graph as a random exchangeable array, i.e. the adjacency matrix (Orbanz and Roy, 2015). Exchangeability in the graphs domain has been historically defined as distribution invariance to the permutation of the order that the nodes appear, i.e. relabelling the nodes does not change the distribution of the graph, and is known as *vertex exchangeability*. However, as a corollary of the Aldous–Hoover theorem (Aldous, 1981; Hoover, 1979), exchangeable random arrays are dense or empty and thus not appropriate for most real applications. In an attempt to account for sparse graphs, several models have been proposed but with undesirable properties. These are models that give up either exchangeability (Barabási and Albert, 1999) or projectivity (Bollobás *et al*., 2007; Bollobás and O’Riordan, 2009; Wolfe and Olhede, 2013; Borgs *et al*., 2014). In this excellent work, the authors propose a model that represents graphs as infinite point processes on $R_{+}^{2}$ giving rise to a class of random graphs ranging from dense to sparse, as tuned by a single parameter. For the associated notion of exchangeability of point processes, Kallenberg (2005), chapter 9, provides a representation theorem as the continuous space counterpart of the Aldous–Hoover theorem, enabling a strong theoretical foundation.

The work, proposed by François Caron and Emily Fox, has stimulated exciting research: the framework is being used as the building block for a series of interesting extensions. One example is incorporating structure in the network in terms of community affiliations of the nodes (Todeschini and Caron, 2016) or accounting for node attributes. Also, the dynamic version of the model is of interest, where the node parameters might evolve over time (Palla *et al*., 2016).

**Daniel M. Roy and Victor Veitch** (*University of Toronto*)

Caron and Fox have made several fundamental contributions, leading to significant progress in our understanding of statistical network modelling. In particular, their work has inspired the development of a complete generalization of the dense exchangeable (graphon) framework to a framework that can also capture sparse graphs (Veitch and Roy, 2015; Borgs *et al*., 2016; Veitch and Roy, 2016; Janson, 2017).

The new framework makes use of Caron and Fox's identification of random graphs and random measures, and the associated notion of exchangeability. An application of Kallenberg's representation theorem for exchangeable measures on the plane implies that the distribution of every extremal sparse exchangeable graph admits a simple representation in terms of a structure we call a *graphex*, by analogy with the graphon underlying dense exchangeable graphs.

The essential character of the dense exchangeable model is preserved: edges are formed independently of one another, conditionally on latent variables associated with each vertex. Whereas graphons are associated with probability spaces (and independent and identically distributed sequences of latent representations), graphexes are associated with *σ*‐finite measure spaces (and Poisson point processes of latent representations). Dense (graphon) models can be seen to correspond to graphexes where the measure space is finite. Another key difference is the appropriate notion of size: nested dense graph sequences can be indexed by the integer number of vertices, whereas the number of vertices in nested sparse graph sequences grows by random increments.

The analysis by Caron and Fox and in follow‐up work by other researchers shows that the sparse exchangeable model can capture a wide range of the empirical phenomena observed in real world networks but outside the purview of the dense exchangeable model. As dense graphon models are in routine use in graph limit theory, statistical network analysis and machine learning, sparse exchangeable models yield novel extensions and generalizations of practical interest. Further, the completely random‐measure approach introduced by Caron and Fox gives a practical method for defining sparse exchangeable models, which has already been used to extend important model classes (Herlau *et al*., 2016; Todeschini and Caron, 2016).

**Victor Veitch and Daniel M. Roy** (*University of Toronto*)

Caron and Fox identify random graphs with random measures and use this construction to define *sparse exchangeability*. This notion of exchangeability is central both to their model and to the generalizations it has inspired (Veitch and Roy, 2015; Borgs *et al*., 2016). A different perspective on the random‐measure construction and the associated notion of sparse exchangeability helps to clarify the strengths and limitations of the new modelling approach, and its relationship to exchangeable dense graphs.

Probabilistic symmetries are closely connected to sampling design (Orbanz, 2017). Sparse exchangeability can be understood in terms of *p‐sampling*, a subgraph sampling scheme whereby one produces a subgraph from a larger graph by including each vertex independently with probability *p*>0, and dropping any isolated vertices in the induced subgraph (Veitch and Roy, 2016). For every *β*>*α*>0, if *ξ*_*β*_ is a size *β* graph generated by a graphex W, then an *α*/*β*‐sampling of *ξ*_*β*_ is equal in distribution to a size *α* graph *ξ*_*α*_ generated by W. By contrast, for dense exchangeable graphs, if *G*_*n*_ is an *n*‐vertex random graph generated by a graphon *W*, then a random subgraph induced by sampling *k* vertices at random from *G*_*n*_ is equal in distribution to a *k*‐vertex graph generated by *W*.

The sampling perspective highlights a key distinction between the sparse and dense exchangeable models: the former excludes isolated vertices. This is often not a problem in practice, so the flexibility of the Caron–Fox model and its descendants seems to come at little cost.

Exchangeability has considerable utility: *p*‐sampling yields a graph analogue of the empirical measure, which has been shown to be a consistent non‐parametric estimator for the graphex parameter underlying the sparse exchangeable graphs. This estimator is a powerful tool, and its construction relies critically on sparse exchangeability.

The sampling perspective also highlights a weakness of the sparse exchangeable approach. Independent vertex sampling schemes, including *p*‐sampling, are rarely good models for the data collection process underlying real world network data. The sparse exchangeable models thus retain a key pathology of the dense exchangeable models and, accordingly, seem unlikely to resolve fully the practical problems that motivated Caron and Fox. However, the dense exchangeable models have proven to be very useful in practice, so there is reason to believe that the applicability of the sparse exchangeable model goes beyond its literal remit. The sparse exchangeable model constitutes a significant advancement, and we expect that its development will point the way towards further novel attacks on the core problems.

**Priyantha Wijayatunga** (*Umea University*)

Sometimes we need to construct network or graph models for systems under study, e.g. for network representations of stock markets (Boginski *et al*., 2005) and for functional regionwise connectivity of the human brain (Simpson *et al*., 2013). Then edges of the graph among vertices that are subcomponents of the system, such as returns of stocks and regions of the brain, are inferred by using statistical dependence measures such as Pearson's correlation that are applicable for linear associations, mutual information that can be used for some non‐linear associations and so on. Each edge can be attached with a numerical weight that is determined by the strength of association between two vertices. For subsequent inferential tasks the use of accurate dependence measures can be important. Therefore, some researchers propose to use regression functions in place of dependence measures, mainly because of difficulties in measuring non‐linear relationships accurately (Song *et al*., 2012). For measuring any dependence between discrete random variables, say *X* and *Y*, the so‐called Cramer's *V* or Tchuprow's *T*, or the generalized correlation coefficient proposed in Wijayatunga (2016) can be used, where Wijayatunga's coefficient is defined (following Pearson's correlation coefficient) as a metric‐*M*‐based measure of dependence

$$\rho^{M}\left(X,Y\right)=\frac{M(p^{I},p)}{{\left\{\prod_{p^{X}\in P^{X}}M{\left(p^{I},p^{X}\right)}^{1/|P^{X}|}\prod_{p^{Y}\in P^{Y}}M{\left(p^{I},p^{Y}\right)}^{1/|P^{Y}|}\right\}}^{1/2}}$$

where *p* is the joint probability distribution of *X* and *Y* and *p* ^I^ is that when their independence is assumed, $P^{X}$ denotes the set of all joint probability distributions, each representing a maximal dependence while preserving the marginal distribution of *X* (and similarly for $P^{Y}$), |*A*| is the cardinality of set *A* and *M*(*p*,*q*) is a distance metric between two probability distributions *p* and *q*, e.g. Hellinger distance $M\left(p^{I},p\right)={\left(\frac{1}{2}\Sigma_{x,\;y}{\left[\surd p(x,y)-\surd \left\{p(x)p(y)\right\}\right]}^{2}\right)}^{1/2}$ or similar. Note that this is a normalized metric‐*M*‐based distance between the dependence and the independence, represented by *p*(*x*,*y*) and *p*(*x*)*p*(*y*) respectively. If there are many maximal dependences (when the respective marginal is fixed), i.e. cardinalities of $P^{X}$ or $P^{Y}$ are big, then one can eliminate some of them subjectively. Ideally these cardinalities are 1.

This can be generalized for continuous variables; however, with Hellinger distance the required normalizing constant is 1. Therefore one can use the measure $\rho^{H}\left(X,Y\right)={\left(\frac{1}{2}\int_{x,\;y}{\left[\surd f(x,y)-\surd \left\{f(x)f(y)\right\}\right]}^{2}dxdy\right)}^{1/2}$ similar to that proposed in Granger *et al*. (2004), where *f* represents the respective probability density functions. In these cases, it is tried to measure strengths of dependences accurately; therefore it is interesting to see differences between network models constructed from such measures and those obtained from traditional linear measures of dependences.

**Mingyuan Zhou** (*University of Texas at Austin*)

I congratulate Professor Caron and Dr Fox for a well‐written paper that establishes a novel statistical network modelling framework, which uses completely random measures to model graphs with various levels of sparsity. Although it has been made clear in the paper that the characteristics of the underlying completely random measure, e.g. the discount parameter *σ*<1 of the generalized gamma process, play a crucial role in determining the levels of sparsity of the generated graphs, I should like to call attention to the potentially important role played by the link function *f*(*x*)=1− exp (−*x*), in generating unweighted undirected sparse graphs with $z_{ij}|w_{i},w_{j}∼\text{Bernoulli}\left\{f\left(2w_{i}w_{j}\right)\right\}$ for *i*≠*j*. With this link function, the contribution of *z*_*ij*_ to the negative log‐likelihood of the model can be expressed as

$$-z_{ij}\ln\left\{1-\exp\left(-2w_{i}w_{j}\right)\right\}+2\left(1-z_{ij}\right)w_{i}w_{j},$$

which quickly explodes towards ∞ as the product *w*_*i*_ *w*_*j*_ approaches 0 when *z*_*ij*_=1. Thus the choice of this link function implies an inductive bias towards fitting non‐zero edges *z*_*ij*_=1, while not strongly penalizing zero edges *z*_*ij*_=0 even if their corresponding products *w*_*i*_ *w*_*j*_ are large. The same link function, which is referred to as the Bernoulli–Poisson link, has also been used in Zhou (2015), which constructs non‐parametric Bayesian network models for overlapping community detection and missing link prediction, allowing the computation to scale linearly with the number of edges, rather than quadratically with the number of nodes. It would be of interest to articulate the role of this specific link function in supporting sparse graphs under the framework proposed.

In addition to controlling for sparse graphs how the number of edges increases as a function of the number of nodes, another topic worth further investigation is how to introduce structured sparsity patterns to the graph adjacency matrices, including modelling dense on‐diagonal but sparse off‐diagonal blocks, sparse on‐diagonal but dense off‐diagonal blocks or a mixture of both. It would be interesting to find out whether the new notion of exchangeability could be maintained while achieving these network modelling goals.

The **authors** replied later, in writing, as follows.

We thank the discussants for the very interesting and thought‐provoking comments. As recalled by Bianconi, one of the successes of *network science*—besides the ubiquitous presence of network data—lies in ‘the ability of the field to adopt methods and techniques coming from different theoretical disciplines’. We certainly agree with this statement. The very diverse panel of discussants for this paper, with research interests in statistical mechanics, graph theory, applied probability, mathematical statistics, Bayesian statistics and machine learning, demonstrates this and provides a wide range of insights on our paper.

*Follow‐up theoretical work and generalizations of the graphon*

The first version of the paper appeared in arXiv in January 2014, and this work has since inspired several other works, as pointed out by Borgs and Chayes, Veitch and Roy, Janson and Orbanz. In particular, the work of Veitch and Roy (2015) and Borgs *et al*. (2016) showed how the representation of a graph as an exchangeable point process leads to a natural generalization of the classical (dense) graphon framework as functions over probability spaces to functions over *σ*‐finite spaces. The class of graphs is called *Kallenberg exchangeable graphs* by Veitch and Roy (2015) and *graphon processes* by Borgs *et al*. (2016). Starting from Kallenberg's representation theorem (theorem 1) in the special case of exchangeable point processes, and keeping only the first term on the right‐hand side of equation (5) which captures most of the interesting structure (see Veitch and Roy (2015) for an interpretation of the other terms), the point process admits the representation of equation (1) where

$$z_{ij}|M,{\left(\theta_{k},\vartheta_{k}\right)}_{k=1,\;2,\;\ldots }∼\text{Bernoulli}\left\{M\left(\vartheta_{i},\vartheta_{j}\right)\right\}.$$

Here, $\left\{{\left(\theta_{i},\vartheta_{i}\right)}_{i=1,\;2,\;\ldots }\right\}$ are the points of a unit rate Poisson process on $R_{+}^{2}$. The symmetric measurable function $M:R_{+}^{2}\to \left[0,1\right]$ (Veitch and Roy (2015) and Borgs *et al*. (2016) use the notation *W*, by analogy with standard graphon notation; for consistency with the rest of the paper, we use *M* here as in Section 5.l), which must satisfy some integrability conditions, is the analogue of the (dense) graphon and called a *graphex* by Veitch and Roy (2015), or just *graphon* by Borgs *et al*. (2016). If the function *M* has compact support, this corresponds to the classical (dense) graphon framework. Some properties of this general class of network models, as well as the convergence of the network process to the limiting graphex or graphon object, are analysed in detail by Veitch and Roy (2015), Borgs *et al*. (2016) and Janson (2017b).

Borgs *et al*. (2016) considered a framework where *M*:*S*×*S*→[0,1] with *S* some potentially high dimensional feature space—not necessarily $R_{+}$—and $\left(\theta_{i},\vartheta_{i}\right)$ the points of a Poisson point process on $R_{+}\times S$ with mean measure d*θμ*(d*ϑ*). Although both constructions lead to the same family of random graphs, it may be more natural, as noted by Janson, and Borgs and Chayes, to work with the higher dimensional feature space as this may lead to more interpretable representations.

*More structured models based on exchangeable random measures*

In this paper, we have introduced the general framework of representing random graphs by exchangeable random measures. Whereas the graphon framework involves a transformation of uniform random variables, hence leading to models with random vectors as building blocks, our framework involves transformations of unit rate Poisson processes, hence leading to models with multivariate point processes or completely random measures as building blocks. We focused on a particular class of models within this framework, defined by equations (6)–(8). We showed that it is possible to capture both sparse and dense graphs with interpretable parameters and scalable inference algorithms.

The class of models that we considered is quite simple, with a single parameter *w*_*i*_ tuning the sociability of a node *i*. Although this model can capture sparsity and heavy‐tailed degree distributions, it cannot capture more local structure, such as latent communities. As shown in Section 5.1, our considered model is just a particular example within Kallenberg's representation theorem, which can be used as a recipe to construct more structured graphs, multigraphs or weighted graphs based on exchangeable random measures. As discussed by Griffin and Leisen, Palla and Miscouridou, Roy and Veitch, and Rubin‐Delanchy, more structured models have already been proposed within this framework, including a sparse stochastic block model (Herlau *et al*., 2016) and a mixed membership stochastic block model (Borgs *et al*., 2016), and a generalization to $R_{+}^{p}$ of the model defined by equations (6)–(8) in this paper to capture overlapping communities (Todeschini and Caron, 2016). Zhou also suggests exploring other stochastic block models with structured sparsity properties.

Besides the above work on more structured models for simple graphs, a few discussants mentioned other possible extensions. Durante, and Lijoi, Mena and Prünster suggest models for collections of networks, building on dependent completely random measures. James and Heaukulani suggest an extension of the model to deal with hypergraphs, building on the Indian buffet process. Palla and Miscouridou consider extensions to dynamic networks. Finally, Bharath and Rubin‐Delanchy suggest the use of inhomogeneous completely random measures to deal with covariates. These ideas represent interesting directions to explore and demonstrate how our framework provides a foundation on which it is possible to expand in many ways.

*Sampling and projectivity*

Many discussants commented on the projectivity properties and sampling mechanism that is associated with our network process. In our setting, each node *i* is associated an arrival time $\theta_{i}$, and the model is invariant over any measure preserving transformation of time. Our construction is therefore exchangeable in this sense. For some *α*>0, we consider only interactions between nodes that arrived before time *α*; therefore, *α* tunes the sample size of the graph.

The notion of sampling that is naturally associated with this class of graph processes has been defined in the follow‐up work of Veitch and Roy (2016). As recalled in the comments of Veitch and Roy, and Orbanz: for a given graph, sample a subgraph by choosing each vertex with probability *p* ∈ (0,1); then discard the isolated nodes. If we consider an exchangeable graph of size *α*, then *p*‐sampling gives a subgraph with the same distribution, but of size *pα*. As mentioned by Bharath, Veitch and Roy, Orbanz and Crane, this sampling scheme highlights a structural weakness of the exchangeable graph process approach: independently sampling vertices may appear unrealistic as a network sampling strategy in some situations.

Nonetheless, as recalled by Veitch and Roy, the (dense) graphon models share the same limitations but have proven to be extremely useful in a large number of applications. An important advantage of the approach, which we emphasize in this paper, is that the exchangeability property enables the design of scalable inference algorithms. We believe that this is a key aspect for the method to be widely applied.

*Applications and extensions*

We provide a simple illustration of our approach in Section 8.2 by assessing the sparsity of the graph. As pointed out by Bharath, this applied analysis is rather limited, notably for brevity, and because the focus of the paper is on the theoretical developments of the exchangeable graph process framework and its associated Markov chain Monte Carlo (MCMC) sampling. The point of this section is primarily to demonstrate that the algorithms developed can be applied to quite large real world graphs. We investigate the fit of the model by looking at posterior predictive degree distributions with the goal of showing that the model provides a reasonable fit, not to test whether or not the real networks have power law degree distributions as in Clauset *et al*. (2009).

As mentioned earlier in this rejoinder, more structured models have been used to uncover latent structure and to link prediction (Herlau *et al*., 2016; Todeschini and Caron, 2016). A few discussants mentioned other interesting areas in which the framework proposed can be applied. Ni and Müller suggest using the model as a prior for structure learning in probabilistic graphical models, i.e. our graphs encode the *latent* conditional independence statements that one aims to infer from a set of observations. Bharath suggests an application to extreme event detection, whereas Battiston and Favaro discuss an application to privacy disclosure. We believe that there is a vast set of possible applications where our defined statistical network model—and extensions thereof—will prove useful.

*Related approaches*

A few discussants have mentioned connections with various related network models. Bianconi and Crane discuss the connection of our approach with the Barabasi–Albert (BA) preferential attachment model, which is a generative model that can capture sparsity and power law degree distributions. As mentioned by Bianconi, the BA model can achieve asymptotic power law degree distributions with exponent *γ* ∈ (2,3]. Follow‐up work (Caron and Rousseau, 2017) showed that one can achieve asymptotic power law degree distributions with exponent *γ* ∈ (1,2) in our framework. Crane questions the usefulness of the proposed approach compared with the BA model.

- As mentioned earlier, this paper is part of a line of work that offers a generalization of graphon models to sparse graphs and therefore offers a rich framework for building sparse graphs. Many references have already built on our work to construct more structured models (Herlau *et al*., 2016; Borgs *et al*., 2016; Todeschini and Caron, 2016). Although some extensions of the BA model to capture community structure exist, they are not straightforward to define and do not fit in a unified framework. Therefore, as mentioned by Crane, this has limited their use as statistical models.
- In the (non‐exchangeable) BA model, one needs to know the ordering of the nodes to make inference on the parameters of the model. If this ordering is not known, it needs to be imputed as a latent variable, severely limiting the scalability of the resulting algorithm. Because of the exchangeability property of our approach, we avoid this issue. The labelling of the nodes is irrelevant in our approach, and we can additionally make use of the hierarchical structure to design scalable algorithms, as demonstrated in Section 7.

Another related line of work that was mentioned by several discussants (Bharath, Crane, Campbell and Broderick, and Bloem‐Reddy) are edge exchangeable models (Crane and Dempsey, 2015, 2016; Cai *et al*., 2016; Williamson, 2016; Janson, 2017a). This line of work—which first appeared in arXiv (Crane and Dempsey, 2015) nearly 2 years after our initial arXiv posting—considers a different notion of exchangeability for ‘interaction data’ defining multi(hyper)graphs (Crane and Dempsey, 2016); as with our method, this approach can also produce sparse graphs.

We believe that both approaches have merit, and that one does not subsume nor replace the other. Both serve as important building blocks for statistical network models. As has been noted by several researchers (Cai *et al*., 2016; Janson, 2017a; Williamson, 2016) and in the discussion of Bloem‐Reddy, there are some similarities between the two approaches, especially as relate to our construction of directed multigraphs in Section 3.2. Although we present a construction for multigraphs, the focus of our paper is on simple graphs. However, the general framework still provides a notion of exchangeability that applies to both multigraphs and simple graphs. Interestingly, this is not so for edge exchangeable models. Simple graphs are obtained from multigraphs by discarding multiple edges (Crane and Dempsey (2016), section 5.4). Through this process, the edge exchangeability property is lost.

Alternative ideas for constructing non‐projective sparse multigraphs are presented by Kumar. Kendall, and Mateu and Eckardt discuss other related approaches based on stochastic geometry. Mateu and Eckardt mention the similarities with geometric or neighbour graphs, which also use (spatial) point processes for random graphs. Finally, Kendall discusses the class of scale invariant random spatial networks, which has a different notion of (spatial) invariance and uses (improper) Poisson line processes. Though these constructions are quite different from our own, they offer another illustration of the usefulness of stochastic processes for building network models.

*Further properties of the class of random graphs*

In this paper, we focused on the sparsity of the resulting networks defined by equations (6)–(8) when *ρ* has finite mean. The sparsity properties are not tied to the specific choice of the link function (see the comment by Zhou) and follow‐up work studied the sparsity properties of general Kallenberg graphs. Veitch and Roy (2015) studied the expected number of edges and nodes for the general model, showing that sparsity is achieved whenever the function *M* is integrable with unbounded support; Caron and Rousseau (2017) provide strong laws for the asymptotic number of edges and nodes and degree distributions when *M* is integrable and satisfies some regular variation assumptions. Bianconi asks whether it is possible to achieve the extremely sparse regime in this framework, $N_{\alpha }^{\left(e\right)}=O\left(N_{\alpha }\right)$ almost surely. Caron and Rousseau (2017) showed that it is possible to obtain rates of $N_{\alpha }^{\left(e\right)}=\Theta \left\{N_{\alpha }l^{*}\left(N_{\alpha }\right)\right\}$ where *l* ^*^ is a slowly varying function with $l^{*}\left(t\right)\to \infty$. If *ρ* has finite mean, or more generally if *M* is integrable, $E\left[N_{\alpha }\right]=o\left(\alpha^{2}\right)$; hence one cannot achieve the extremely sparse regime.

It would be of interest to characterize other properties of the special class of graphs defined by equations (6)–(8), and more generally of graphs based on Kallenberg's representation. Casarin, Iacopini and Rossini investigate empirically the distribution of the assortativity coefficient and clustering coefficient (also discussed by Durante and Chakraborty) for the generalized gamma process model. Banerjee and Ghosal suggest investigating the characterization of the graph Laplacian and the limit distribution of the normalized number of edges. These represent some important possible directions of exploration.

*Consistency and rates of convergence*

Questions regarding the convergence of posterior distributions of the parameters, hyperparameters or functionals have been highlighted by a few discussants. Arbel suggests an approach to show posterior consistency for the sparsity parameter *σ*. Such results, in a well‐specified and misspecified setting, would be useful, as this would justify the use of the method that is described in Section 8.1 as a sparsity test. Castillo and Rebafka also study empirically the asymptotic behaviour of the posterior distribution on *σ* in a misspecified scenario, when simulating from a sparse, but non‐projective, model. They also investigated empirically the convergence of the posterior of the edge density and density of triangles.

Gao (see also Rubin‐Delanchy and Chakraborty) draws connections with the rich literature on frequentist estimation of the parameters of network models, such as the stochastic block model. It is not obvious to us, however, how these tools could be applied in the sparse regime. In this case, the number of isolated nodes with sociability *w*_*i*_>0 is infinite for any given *α*, which is different from the framework that was described by Gao, corresponding closely to the dense case. Let (*w*_*i*_) be the sociabilities of the nodes such that $\theta_{i}<\alpha$ and $N_{\alpha }$ the number of nodes with at least one edge. Our likelihood, for the directed multigraph, is given by

$$\text{Pr}\left\{{\left(n_{ij}\right)}_{1⩽i,\;j⩽N_{\alpha }}|\left(w_{k}\right)\right\}\propto \exp\left\{-{\left(\sum_{k=1}^{\infty }w_{k}\right)}^{\;\;2}+{\left(\sum_{i=1}^{N_{\alpha }}w_{i}\right)}^{\;\;2}\right\}\prod_{1⩽i,\;j⩽N_{\alpha }}\frac{{\left(w_{i}w_{j}\right)}^{n_{ij}}}{n_{ij}!}\exp\left(-w_{i}w_{j}\right).$$

In contrast, the likelihood defined by Gao differs in the first term and corresponds to a model with a finite number of nodes. This also clarifies a question from Moores and Firth: the weights of isolated nodes with arrival time $\theta_{i}<\alpha$ appear in the likelihood, and their sum is identifiable (but not the individual weights).

Although we provided some important analyses of the properties of our modelling framework, there are many other interesting aspects to study relating to the asymptotic properties of inference and estimation.

*Computational aspects*

This paper presented an MCMC algorithm with Hamiltonian Monte Carlo (HMC) and Metropolis–Hastings updates. The key step underlying our scheme is a data augmentation that allows a very simple expression for the gradient in the HMC update and tailored proposals for the hyperparameters. There is, however, plenty of room for improvement in the efficiency of our MCMC algorithm. Our HMC implementation is very simple, with a fixed number of leapfrog steps, and the step size adapted during part of the burn‐in period. More elaborate HMC strategies could be used here. As mentioned by Moores and Firth and briefly discussed in Section 7.2 of our paper, the algorithm is very amenable to parallelism. Alternatives to HMC sampling have been implemented by Bouchard‐Côté and Briercliffe, showing that additional gains could be obtained through such modifications as well. Alternatively, Campbell and Broderick suggest using variational Bayesian instead of MCMC algorithms, applying a truncation scheme for the completely random measure. This also represents a promising alternative to our MCMC algorithm.

## Figures and Tables

**Figure 14:**
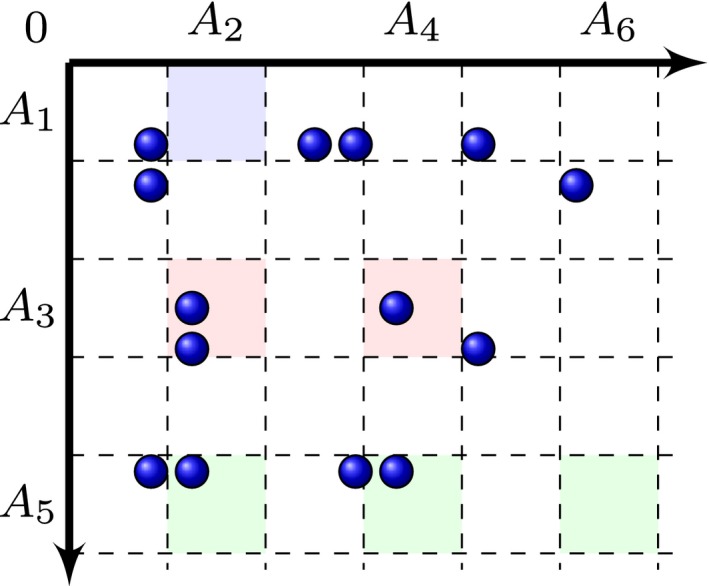
Illustration of the proof of theorem 4 in the infinite activity case (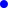, support points of the measure *D*): in this example, the coloured regions indicate the intersection of *A*_2*n*−1_ with $S_{n}^{\left(2\right)}$ for *n*=1 (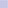), *n*=2 (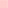), and *n*=3 (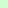); from this, we see that *X*_1_=0 (the count in blue), *X*_2_=2 (the count in red) and *X*_3_=1 (the count in green)

## Appendix A Further background

### A.1. Aldous–Hoover theorem and graphons

In theorem 7, we present the Aldous–Hoover theorem for the case of *joint exchangeability*—i.e. symmetric permutations of rows and columns—which is applicable to matrices *Z* where both rows and columns index the same set of nodes. *Separate exchangeability* allows for different row and column permutations, making it applicable to scenarios where one has distinct node identities on rows and columns, such as in the bipartite graphs that we considered in Section 3.4. Extensions to higher dimensional arrays are likewise straightforward. For a more general statement of the Aldous–Hoover theorem, which holds for separate exchangeability and higher dimensional arrays, see Orbanz and Roy (2015).

Theorem 7 (Aldous–Hoover representation of jointly exchangeable matrices (Aldous, 1981; Hoover, 1979)). A random matrix ${\left(Z_{ij}\right)}_{i,j\in N}$ is jointly exchangeable if and only if there is a random measurable function *f*:[0,1]^3^→**Z** such that

(46) $$\left(Z_{ij}\right)\overset{d}{=}\left(f\left(U_{i},U_{j},U_{ij}\right)\right),$$

where ${\left(U_{i}\right)}_{i\in N}$ and ${\left(U_{ij}\right)}_{i,j>i\in N}$ with *U*_*ij*_=*U*_*ji*_ are a sequence and matrix respectively of IID uniform[0,1] random variables.

For undirected graphs where *Z* is a binary, symmetric adjacency matrix, the Aldous–Hoover representation can be expressed as the existence of a *graphonM*:[0,1]^2^→[0,1], symmetric in its arguments, where

(47) $$f\left(U_{i},U_{j},U_{ij}\right)=\left\{\begin{array}{cc} 1 & U_{ij}<M\left(U_{i},U_{j}\right),\\ 0 & \text{otherwise}. \end{array}\right.$$

### A.2. Regularly varying Lévy measures

Here we provide a formal definition of a regularly varying Lévy measure *ρ* that is referred to in theorems 2 and 5.

Definition 1 (regular variation). A Lévy measure *ρ* on (0,∞) is said to be *regularly varying* if its tail Lévy intensity $\overset{¯}{\rho }\left(x\right)=\int_{x}^{\infty }\rho \left(dw\right)$ is a regularly varying function (Feller, 1971), i.e. it satisfies

(48) $$\overset{¯}{\rho }\left(x\right)\overset{x↓0}{∼}l\left(1/x\right)x^{-\sigma }$$

for *σ* ∈ [0,1) and *l* a slowly varying function satisfying $\lim_{t\to \infty }l\left(at\right)/l\left(t\right)=1$ for any *a*>0.

For example, constant and logarithmic functions are slowly varying.

## Appendix B Proof of proposition 1

The proof of proposition 1 follows from the properties of *W*∼CRM(*ρ*,*λ*). Let *A*_*i*_=[*h*(*i*−1),*hi*] for *h*>0 and i∈N. We have

(49) $$\left(W\left(A_{i}\right)\right)\overset{d}{=}\;\left(W\left(A_{\pi (i)}\right)\right)$$

for any permutation *π* of N. As *D*(*A*_*i*_×*A*_*j*_)∼Poisson{*W*(*A*_*i*_)*W* (*A*_*j*_)}, it follows that

(50) $$\left(D\left(A_{i}\times A_{j}\right)\right)\overset{d}{=}\;\left(D\left(A_{\pi (i)}\times A_{\pi (j)}\right)\right)$$

for any permutation *π* of N. Joint exchangeability of *Z* follows directly.

## Appendix C Proofs of results on the sparsity

### C.1. Probability asymptotics notation

We first describe the asymptotic notation that is used in the remainder of this section, which follows the notation of Janson (2011). All unspecified limits are as *α*→∞.

Let (*X*_*α*_)_*α*⩾0_ and (*Y*_*α*_)_*α*⩾0_ be two [0,∞)‐valued stochastic processes defined on the same probability space and such that $\lim_{\alpha \to \infty }X_{\alpha }=\lim_{\alpha \to \infty }Y_{\alpha }=\infty$ almost surely. We have

$$\begin{array}{cc} & X_{\alpha }=O\left(Y_{\alpha }\right)\;\text{almost surely}\Leftrightarrow \underset{\alpha \to \infty }{\lim\sup}X_{\alpha }/Y_{\alpha }<\infty \;\text{almost surely},\\ & X_{\alpha }=o\left(Y_{\alpha }\right)\;\text{almost surely}\Leftrightarrow \lim_{\alpha \to \infty }X_{\alpha }/Y_{\alpha }=0\;\text{almost surely},\\ & X_{\alpha }=\Omega \left(Y_{\alpha }\right)\;\text{almost surely}\Leftrightarrow Y_{\alpha }=O\left(X_{\alpha }\right)\;\text{almost surely},\\ & X_{\alpha }=\omega \left(Y_{\alpha }\right)\;\text{almost surely}\Leftrightarrow Y_{\alpha }=o\left(X_{\alpha }\right)\;\text{almost surely},\\ & X_{\alpha }=\Theta \left(Y_{\alpha }\right)\;\text{almost surely}\Leftrightarrow X_{\alpha }=O\left(Y_{\alpha }\right)\;\text{and}\;X_{\alpha }=\Omega \left(Y_{\alpha }\right)\;\text{almost surely}. \end{array}$$

The equivalence notation $f\left(x\right){∼}^{x↓0}g\left(x\right)$ is used for  lim _*x*→0_ *f*(*x*)/*g*(*x*)=1 (not to be confused with the notation ‘∼’ alone for ‘distributed from’).

### C.2. Proof of theorem 3

Assume the moment condition $0<\int_{0}^{\infty }w\rho \left(dw\right)<\infty .$ Let

(51) $${\tilde{Z}}_{ij}=\left\{\begin{array}{cc} 1 & \text{if}\;Z([i-1,i]\times [j-1,j])>0,\\ 0 & \text{otherwise} \end{array}\right.$$

and ${\tilde{D}}_{ij}=D\left(\left[i-1,i\right]\times \left[j-1,j\right]\right)$. Then, almost surely for any k∈N,

(52) $$\sum_{1⩽i<j⩽k}{\tilde{Z}}_{ij}⩽N_{k}^{\left(e\right)}⩽\sum_{1⩽i,j⩽k}{\tilde{D}}_{ij}.$$

As *Z* is a jointly exchangeable point process, ${\left({\tilde{Z}}_{ij}\right)}_{i,j\in N}$ is a jointly exchangeable binary matrix, and so

(53) $$\sum_{1⩽i<j⩽k}{\tilde{Z}}_{ij}=\Theta \left(k^{2}\right)\;\;\text{almost surely as}\;k\to \infty .$$

Moreover, we have

(54) $${\tilde{D}}_{ij}|W\overset{\mathrm{ind}}{∼}\text{Poisson}\left\{W\left(\left[i-1,i\right]\right)W\left(\left[j-1,j\right]\right)\right\}$$

So lemma 1 in Appendix D and the strong law of large numbers for *V*‐statistics (Arcones and Giné, 1992; Giné and Zinn, 1992) imply that

(55) $$\sum_{1⩽i,j⩽k}{\tilde{D}}_{ij}=\Theta \left(k^{2}\right)\;\;\text{almost surely as}\;k\to \infty .$$

We therefore conclude that $N_{k}^{\left(e\right)}=\Theta \left(k^{2}\right)\;\text{almost surely as}\;k\to \infty .$ Finally, for any *k*⩽*α*⩽*k*+1,

$$\frac{k^{2}}{{\left(k+1\right)}^{2}}\frac{N_{k}^{\left(e\right)}}{k^{2}}⩽\frac{N_{\alpha }^{\left(e\right)}}{\alpha^{2}}⩽\frac{{\left(k+1\right)}^{2}}{k^{2}}\frac{N_{k+1}^{\left(e\right)}}{{\left(k+1\right)}^{2}}$$

and as (*k*+1)/*k*→1 we conclude that

(56) $$N_{\alpha }^{\left(e\right)}=\Theta \left(\alpha^{2}\right)\;\;\text{almost surely as}\;\alpha \to \infty .$$

### C.3. Proof of theorem 4

#### C.3.1. Finite activity case

We first consider the case of a finite activity CRM. In this case, the number of nodes is bounded below by the square root of the number of edges, and bounded above by the (finite) number of jumps of the CRM. Let $T=\int_{0}^{\infty }\rho \left(dw\right)$, 0<*T*<∞. Let *J*_*α*_ denote the number of points $\left(w_{i},\theta_{i}\right)$ such that $\theta_{i}<\alpha$. (*J*_*α*_)_*α*⩾0_ is a homogeneous Poisson process of rate *T*, and thus *J*_*α*_/*α*→*T* almost surely. Since $\surd N_{\alpha }^{\left(e\right)}⩽N_{\alpha }⩽J_{\alpha }\;\text{almost surely}$, it follows from result (56) that $N_{\alpha }=\Theta \left(\alpha \right)\;\text{almost surely as}\;\alpha \to \infty$.

#### C.3.2. Infinite activity case

We now consider the infinite activity case where $\int_{0}^{\infty }\rho \left(dw\right)=\infty$. First note that, by monotone convergence, $\lim_{t\to \infty }\psi \left(t\right)=\infty$.

Consider sets *A*_*k*_=[(*k*−1)/2,*k*/2) for *k*=1,2,…, and let $S_{n}^{\left(1\right)}=\cup_{k=1}^{n}A_{2k-1}$ and $S_{n}^{\left(2\right)}=\cup_{k=1}^{n}A_{2k}$. $S_{n}^{\left(1\right)}$ and $S_{n}^{\left(2\right)}$ define a partition of [0,*n*]. For n∈N, define the random variable *X*_*n*_ as

(57) $$X_{n}=\#\left\{\theta_{i}\in A_{2n-1}|D\left(\left\{\theta_{i}\right\}\times S_{n}^{\left(2\right)}\right)>0\right\}$$

and let

$${\tilde{N}}_{\alpha }=\sum_{n=1}^{\left\lfloor \alpha \right\rfloor }X_{n}.$$

See Fig. 14 for an illustration. Clearly, ${\tilde{N}}_{\alpha }$ is a lower bound for the number of nodes:

(58) $${\tilde{N}}_{\alpha }\;⩽\;N_{\alpha }\;\;\text{almost surely}.$$

Using the notation $S^{\left(1\right)}=\cup_{k=1}^{\infty }A_{2k-1}$ and $S^{\left(2\right)}=\cup_{k=1}^{\infty }A_{2k}$, let *W* ^(1)^ and *W* ^(2)^ be respectively the restriction of *W* to the set $S^{\left(1\right)}$ and $S^{\left(2\right)}$. As $S^{\left(1\right)}$ and $S^{\left(2\right)}$ are non‐overlapping and *W* is a CRM, *W* ^(1)^ and *W* ^(2)^ are independent. Integrating over *W* ^(1)^ and using the marking theorem for Poisson processes (see below for more details), we obtain for *n*⩾1

(59) $$X_{n}|W^{\left(2\right)}\overset{\mathrm{ind}}{∼}\text{Poisson}\left[\frac{1}{2}\psi \left\{W\left(S_{n}^{\left(2\right)}\right)\right\}\right].$$

Lemma 1 thus implies that

(60) $$\frac{\sum_{k=1}^{n}X_{k}}{\frac{1}{2}\sum_{k=1}^{n}\psi \left\{W\left(S_{k}^{\left(2\right)}\right)\right\}}\to 1\;\;\text{almost surely}.$$

We have $\lambda (S_{n}^{\left(2\right)})=n/2$ and, using the law of large numbers,

(61) $$\frac{W(S_{n}^{\left(2\right)})}{n/2}\to \int_{0}^{\infty }w\rho \left(dw\right)\;\text{almost surely}.$$

Therefore $\psi \{W\left(S_{n}^{\left(2\right)}\right)\}\to \infty$ almost surely. Its Cesàro mean also diverges and

(62) $$\frac{\sum_{k=1}^{n}\psi \left\{W\left(S_{k}^{\left(2\right)}\right)\right\}}{n}\to \infty \;\;\text{almost surely},$$

which, together with result (60), implies that $\left(1/n\right)\Sigma_{k=1}^{n}X_{k}\to \infty \;\text{almost surely}$. We conclude that ${\tilde{N}}_{\alpha }/\alpha \to \infty \;\text{almost surely}$ and, using inequality (58), $N_{\alpha }/\alpha \to \infty \;\text{almost surely}$.

Consider now the case where $\overset{¯}{\rho }\left(x\right){∼}^{x↓0}l\left(1/x\right)x^{-\sigma }$ where *σ* ∈ (0,1) and *l*(*t*) is a slowly varying function such that ${\lim\inf}_{t\to \infty }l\left(t\right)>0.$ Then lemma 2 in Appendix D implies that ${\lim\inf}_{t\to \infty }\psi \left(t\right)/t^{\sigma }>0$ and thus, using result (61),

$$\underset{n\to \infty }{\lim\inf}\frac{\psi \{W\left(S_{n}^{\left(2\right)}\right)\}}{n^{\sigma }}>0\;\;\text{almost surely}.$$

Riemann integration and the Stolz–Cesàro theorem then imply that

$$\underset{n\to \infty }{\lim\inf}\frac{\sum_{k=1}^{n}\psi \left\{W\left(S_{k}^{\left(2\right)}\right)\right\}}{n^{\sigma +1}}=\underset{n\to \infty }{\lim\inf}\frac{\sum_{k=1}^{n}\psi \left\{W\left(S_{k}^{\left(2\right)}\right)\right\}}{\left(\sigma +1\right)\sum_{k=1}^{n}k^{\sigma }}>0\;\;\;\text{almost surely}$$

and finally $N_{\alpha }=\Omega \left(\alpha^{\sigma +1}\right)$ almost surely as *α*→∞.

##### C.3.2.1. Proof of result (59)

Let *n*⩾1. For any $\theta_{i}\in A_{2n-1}$, let *u*_*i*_ be a binary mark such that

$$\text{Pr}\left(u_{i}=1|W\right)=1-\exp\left\{-w_{i}W\left(S_{n}^{\left(2\right)}\right)\right\}.$$

Conditionally on $W_{n}^{\left(2\right)}$, the marking theorem for Poisson processes implies that the set of points $\left\{\left(w_{i}\right)|\theta_{i}\in A_{2n-1},u_{i}=1\right\}$ is drawn from a Poisson process of intensity $\frac{1}{2}\rho \left(dw\right)\left[1-\exp\left\{-wW\left(S_{n}^{\left(2\right)}\right)\right\}\right]$ and the number *X*_*n*_ of those points is Poisson distributed with rate

$$\int_{0}^{\infty }\frac{1}{2}\rho \left(dw\right)\left[1-\exp\left\{-wW\left(S_{n}^{\left(2\right)}\right)\right\}\right]=\frac{1}{2}\psi \left\{W\left(S_{n}^{\left(2\right)}\right)\right\}.$$

Finally, independence of the *X*_*n*_s follows from the complete randomness of $W_{n}^{\left(1\right)}$.

### C.4. Proof of theorem 5

$$\begin{array}{cc} E[D_{\alpha }^{*}] & =E\left[E\left[D_{\alpha }^{*}|W\right]\right]=E\left[W_{\alpha }^{*2}\right]=E{\left[W_{\alpha }^{*}\right]}^{2}+\text{var}\left(W_{\alpha }^{*}\right)\\ & =\alpha^{2}{\left\{\int_{0}^{\infty }w\rho \left(dw\right)\right\}}^{2}+\alpha \int_{0}^{\infty }w^{2}\rho \left(dw\right) \end{array}$$

where the last line follows from Campbell's theorem.

$$\begin{array}{cc} E[N_{\alpha }^{\left(e\right)}] & =E\left[E\left[N_{\alpha }^{\left(e\right)}|W\right]\right]=E\left[\sum_{i}\left[\left\{1-\exp\left(-w_{i}^{2}\right)\right\}+\frac{1}{2}\sum_{j\neq i}\left\{1-\exp\left(-2w_{i}w_{j}\right)\right\}1_{\theta_{j}⩽\alpha }\right]1_{\theta_{i}⩽\alpha }\right]\\ & =E\left[\sum_{i}\left[\left\{1-\exp\left(-w_{i}^{2}\right)\right\}-\frac{1}{2}\left\{1-\exp\left(-2w_{i}^{2}\right)\right\}\right]1_{\theta_{i}⩽\alpha }\right]+\frac{1}{2}E\left[\sum_{i}\left[\sum_{j}\left\{1-\exp\left(-2w_{i}w_{j}\right)\right\}1_{\theta_{j}⩽\alpha }\right]1_{\theta_{i}⩽\alpha }\right]. \end{array}$$

Using the Palm formula for Poisson point processes (Bertoin, 2006; Daley and Vere‐Jones, 2008)

$$E\left[\sum_{i}\left[\sum_{j}\left\{1-\exp\left(-2w_{i}w_{j}\right)\right\}1_{\theta_{j}⩽\alpha }\right]1_{\theta_{i}⩽\alpha }\right]=\alpha \int_{0}^{\infty }E\left[\left\{1-\exp\left(-2w^{2}\right)\right\}+\sum_{j}\left\{1-\exp\left(-2ww_{j}\right)\right\}1_{\theta_{j}⩽\alpha }\right]\rho \left(dw\right)$$

and the final expression is obtained by applying Campbell's theorem. Finally, the expected number of nodes

$$\begin{array}{cc} E[N_{\alpha }] & =E\left[E\left[N_{\alpha }|W\right]\right]=E\left[\sum_{i}\left\{1-\exp\left(-2w_{i}\sum_{j}w_{j}1_{\theta_{j}⩽\alpha }+w_{i}^{2}\right)\right\}1_{\theta_{i}⩽\alpha }\right]\\ & =\alpha \int_{0}^{\infty }E\left[\left\{1-\exp\left(-2w\sum_{j}w_{j}1_{\theta_{j}⩽\alpha }-w^{2}\right)\right\}\right]\rho \left(dw\right)\\ & =\alpha \int_{0}^{\infty }\left(1-E\left[\exp\left(-2w\sum_{j}w_{j}1_{\theta_{j}⩽\alpha }-w^{2}\right)\right]\right)\rho \left(dw\right)\\ & =\alpha \int_{0}^{\infty }\left[1-\exp\left\{-w^{2}-\alpha \psi \left(2w\right)\right\}\right]\rho \left(dw\right) \end{array}$$

where we successively used the Palm formula and Campbell's theorem. By dominated convergence, $E\left[N_{\alpha }\right]{∼}^{\alpha ↑\infty }\alpha \int_{0}^{\infty }\rho \left(dw\right)$ if the CRM is finite activity. Consider now that the CRM is infinite activity. Using integration by parts, we have

(63) $$E\left[N_{\alpha }\right]=\alpha \int_{0}^{\infty }\left\{2w+2\alpha \psi^{\prime }\left(2w\right)\right\}\exp\left\{-w^{2}-\alpha \psi \left(2w\right)\right\}\overset{¯}{\rho }\left(w\right)dw.$$

The Laplace exponent is a strictly increasing function with *ψ*(0)=0 and $\lim_{t\to \infty }\psi \left(t\right)=\infty$ and therefore admits a well‐defined inverse, denoted $\psi^{-1}:\left[\right.0,\infty )\to \left[\right.0,\infty )$. Using the change of variable *u*=*ψ*(2*w*), we obtain

$$\int_{0}^{\infty }2\psi^{\prime }\left(2w\right)\exp\left\{-w^{2}-\alpha \psi \left(2w\right)\right\}\overset{¯}{\rho }\left(w\right)dw=\int_{0}^{\infty }\exp\left[-{\left\{\psi^{-1}\left(u\right)/2\right\}}^{2}-\alpha u\right]\overset{¯}{\rho }\left\{\psi^{-1}\left(u\right)/2\right\}du.$$

Assume that $\int_{0}^{\infty }w\rho \left(dw\right)<\infty$. Now note that $\psi \left(t\right)\;{∼}^{t↓0}\;t\int_{0}^{\infty }w\rho \left(dw\right)$ and therefore $\psi^{-1}\left(t\right)\;{∼}^{t↓0}\;t/\int_{0}^{\infty }w\rho \left(dw\right)$. If *ρ* is a regularly varying Lévy measure, then

$$\overset{¯}{\rho }\left(x\right)\;\overset{x↓0}{∼}\;l\left(1/x\right)x^{-\sigma }$$

where *σ* ∈ [0,1) and *l* is a slowly varying function, and it therefore follows from lemma 3 in Appendix D and *ψ* ^−1^(0)=0 that

$$g\left(u\right):=\exp\left[-{\left\{\psi^{-1}\left(u\right)/2\right\}}^{2}\right]\overset{¯}{\rho }\left\{\psi^{-1}\left(u\right)/2\right\}\overset{u↓0}{∼}l\left(1/u\right)u^{-\sigma }{\left\{2\int_{0}^{\infty }w\rho \left(dw\right)\right\}}^{\sigma }$$

where *g* is a monotone decreasing function. Applying the Tauberian theorem of proposition 2 in Appendix D, we therefore have

$$\int_{0}^{\infty }\exp\left(-\alpha u\right)g\left(u\right)du\overset{\alpha ↑\infty }{∼}\alpha^{\sigma -1}l\left(\alpha \right)\Gamma \left(1-\sigma \right){\left\{2\int_{0}^{\infty }w\rho \left(dw\right)\right\}}^{\sigma }.$$

Finally, combining the above asymptotics with equation (63), and noting that

$$\int_{0}^{\infty }w\exp\left\{-w^{2}-\alpha \psi \left(2w\right)\right\}\overset{¯}{\rho }\left(w\right)dw=o\left(1\right)$$

by dominated convergence, and $\lim_{\alpha \to \infty }\alpha^{\sigma }l\left(\alpha \right)>0$ for *σ* ∈ [0,1), we obtain

$$E\left[N_{\alpha }\right]\overset{\alpha ↑\infty }{∼}\alpha^{1+\sigma }l\left(\alpha \right)\Gamma \left(1-\sigma \right){\left\{2\int_{0}^{\infty }w\rho \left(dw\right)\right\}}^{\sigma }.$$

## Appendix D Technical lemmas

The following lemma is a corollary of theorem 3, page 239, in Feller (1971).

Lemma 1 Let (*X*_*n*_)_*n*=1,2,…_ be a sequence of mutually independent random variables with arbitrary distribution and such that $\text{var}\left(X_{n}\right)⩽E\left[X_{n}\right]<\infty$. Let $S_{n}=\Sigma_{k=1}^{n}X_{k}$. If

$$\lim_{n\to \infty }E\left[S_{n}\right]=\infty$$

then

$$S_{n}/E[S_{n}]\to 1\;\;\text{almost surely as}\;n\to \infty .$$

Assume for simplicity that $E[X_{n}]>0$ for all *n* (otherwise, consider the subsequence of random variables with strictly positive mean). We have

$$\sum_{k=1}^{n}\frac{\text{var}(X_{k})}{1+E{\left[S_{k}\right]}^{2}}⩽\sum_{k=1}^{n}\frac{E[X_{k}]}{1+E{\left[S_{k}\right]}^{2}}⩽\int_{0}^{\infty }\frac{1}{1+x^{2}}dx<\infty$$

by Riemann integration. The result then follows from theorem 3, page 239, in Feller (1971) with $b_{n}=E\left[S_{n}\right]$.

Lemma 2 (relating tail Lévy intensity and Laplace exponent) (Gnedin *et al*. (2007), propositions 17 and 19). Let *ρ* be a Lévy measure, $\overset{¯}{\rho }\left(x\right)=\int_{x}^{\infty }\rho \left(dw\right)$ be the tail Lévy intensity and $\psi \left(t\right)=\int_{0}^{\infty }\left\{1-\exp\left(-wt\right)\right\}\rho \left(dw\right)$ its Laplace exponent. The following conditions are equivalent:

(64) $$\overset{¯}{\rho }\left(x\right)\overset{x↓0}{∼}l\left(1/x\right)x^{-\sigma },$$

(65) $$\psi \left(t\right)\overset{t↑\infty }{∼}\Gamma \left(1-\sigma \right)t^{\sigma }l\left(t\right)$$

where 0⩽*σ*<1 and *l* is a function slowly varying at ∞, i.e. satisfying *l*(*cy*)/*l*(*y*)→1 as *y*→∞ for every *c*>0.

Lemma 3 (Resnick (1987), chapter 0, proposition 0.8). If *U* is a regularly varying function at 0 with exponent σ∈R, and *f* is a positive function such that *f*(*t*) ∼^*t*↓0^ *tc*, for some constant 0<*c*<∞, then *U*{*f*(*t*)} ∼^*t*↓0^ *c* ^*σ*^ *U*(*t*).

Proposition 2 (Tauberian theorem) (Feller (1971), chapter XIII, section 5, theorems 3 and 4). Let *U*(d*w*) be a measure on (0,∞) with density *u* monotone in some interval (0,*x*_0_). Assume that

$$L\left(t\right)=\int_{0}^{\infty }\exp\left(-tw\right)u\left(w\right)dw$$

exists for *t*>0. If *l* is slowly varying at ∞ and 0 < *a*<∞, then the following two relationships are equivalent:

(66) $$L\left(t\right)\overset{t↑\infty }{∼}t^{-a}l\left(t\right),$$

(67) $$u\left(x\right)\overset{x↓0}{∼}\frac{1}{\Gamma (a)}x^{a-1}l\left(\frac{1}{x}\right).$$

## Appendix E Proof of theorem 6

The proof of theorem 6 relies on results on posterior characterization with models involving normalized CRMs. We first state a corollary of lemma 5 by Pitman (2003) and theorem 8.1 by James (2002). Similar results appear in Prünster (2002), James (2005) and James *et al*. (2009). The corollary involves the introduction of a discrete random variable *R*, conditional on which the CRM has strictly positive mass.

Corollary 1 Let *W*_*α*_ be a (finite or infinite) CRM on [0,*α*] without fixed atoms nor deterministic component, with mean measure *ρ*(d*w*)d*θ*. Denote $W_{\alpha }^{*}=W_{\alpha }\left(\left[0,\alpha \right]\right)$, with probability distribution $G_{\alpha }^{*}$. Let *R* ∈ {0,1,2,…} be a discrete random variable such that, for *r*⩾0,

$$\zeta_{r}\left(t\right):=\text{Pr}\left(R=r|W_{\alpha }^{*}=t\right)$$

with *ζ*_0_(0)=1. The condition $\text{Pr}(R=0|W_{\alpha }^{*}=0)=1$ ensures that, conditionally on *R*>0, $W_{\alpha }^{*}>0$ almost surely, and the normalized CRM below is properly defined.

Conditionally on *R*=*r*>0, let $X_{1},\ldots ,X_{r}{∼}^{\mathrm{IID}}W_{\alpha }/W_{\alpha }^{*}$. Let ${\tilde{\theta }}_{1},\ldots ,{\tilde{\theta }}_{k}$, *k*⩽*r*, be the unique values in (*X*_1_,…,*X*_*r*_), in order of appearance, with multiplicities $1⩽{\tilde{m}}_{j}⩽r$, ${\tilde{w}}_{i}=W_{\alpha }\left(\left\{{\tilde{\theta }}_{i}\right\}\right)$ the associated weights and Π_*r*_={*A*_1_,…,*A*_*k*_} with $A_{i}=\left\{j|X_{j}={\tilde{\theta }}_{i}\right\}$ be the associated random partition of {1,…,*r*}. Let $w_{*}=W_{\alpha }^{*}-\Sigma_{i}{\tilde{w}}_{i}$. For *r*>0, we have

(68) $$\begin{array}{cc} \text{Pr}[R=r,\Pi_{R} & =\left\{A_{1},\ldots ,A_{k}\right\},{\left({\tilde{w}}_{i}\in d{\tilde{w}}_{i}\right)}_{i=1,\ldots k},w_{*}\in dw_{*}]\\ & ={\left(w_{*}+\sum_{i=1}^{k}{\tilde{w}}_{i}\right)}^{-r}\zeta_{r}\left(w_{*}+\sum_{i=1}^{k}{\tilde{w}}_{i}\right)G_{\alpha }^{*}\left(dw_{*}\right)\alpha^{k}\prod_{i=1}^{k}{\tilde{w}}_{i}^{{\tilde{m}}_{i}}\rho \left(d{\tilde{w}}_{i}\right) \end{array}$$

and

$$\text{Pr}\left(R=0,w_{*}\in dw_{*}\right)=\zeta_{0}\left(w_{*}\right)G_{\alpha }^{*}\left(dw_{*}\right).$$

We now prove theorem 6. Consider the conditionally Poisson construction that was described in Section 5.5:

$$\begin{array}{cc} & D_{\alpha }^{*}|W_{\alpha }^{*}∼\text{Poisson}\left(W_{\alpha }^{*\;2}\right),\\ & \left(U_{1}^{\prime },\ldots ,U_{2D_{\alpha }^{*}}^{\prime }\right)|D_{\alpha }^{*},W_{\alpha }\overset{\mathrm{IID}}{∼}W_{\alpha }/W_{\alpha }^{*}. \end{array}$$

First, define, for *r* ∈ {0,2,4,…} and *t*⩾0,

$$\zeta_{r}\left(t\right):=\text{Pr}\left(2D_{\alpha }^{*}=r|W_{\alpha }^{*}=t\right)=\frac{t^{r}\exp\left(-t^{2}\right)}{(r/2)!}$$

with *ζ*_0_(0)=1. Conditionally on $2D_{\alpha }^{*}=r>0$, $U_{1}^{\prime },\ldots ,U_{r}^{\prime }$ are IID from $W_{\alpha }/W_{\alpha }^{*}$. The variables $U_{1}^{\prime },\ldots ,U_{r}^{\prime }$ take $N_{\alpha }⩽r$ distinct values ${\tilde{\theta }}_{j}$, with multiplicities $1⩽{\tilde{m}}_{j}⩽r$. Let $\Pi_{r}=\left\{A_{1},\ldots ,A_{N_{\alpha }}\right\}$ be the associated partition of {1,…,*r*}. From corollary 1 we have, for *r* ∈ {2,4,6,…},

(69) $$\begin{array}{cc} \text{Pr}[D_{\alpha }^{*} & =r/2,\Pi_{2D_{\alpha }^{*}}=\left\{A_{1},\ldots ,A_{N_{\alpha }}\right\},{\left({\tilde{w}}_{i}\in d{\tilde{w}}_{i}\right)}_{i=1,\ldots N_{\alpha }},w_{*}\in dw_{*}]\\ & =\frac{1}{(r/2)!}\exp\left\{-{\left(w_{*}+\sum_{i=1}^{N_{\alpha }}{\tilde{w}}_{i}\right)}^{2}\right\}G_{\alpha }^{*}\left(dw_{*}\right)\alpha^{N_{\alpha }}\prod_{i=1}^{N_{\alpha }}{\tilde{w}}_{i}^{{\tilde{m}}_{i}}\rho \left(d{\tilde{w}}_{i}\right) \end{array}$$

and

(70) $$\text{Pr}\left(D_{\alpha }^{*}=0,w_{*}\in dw_{*}\right)=\exp\left(-w_{*}^{2}\right)G_{\alpha }^{*}\left(dw_{*}\right).$$

Finally, let *π* be the permutation of $\left\{1,\ldots ,N_{\alpha }\right\}$ such that ${\tilde{\theta }}_{\pi (1)}<{\tilde{\theta }}_{\pi (2)}<\;\;\ldots <{\tilde{\theta }}_{\pi (N_{\alpha })}$ and let $w_{i}={\tilde{w}}_{\pi (i)}$ and $m_{i}={\tilde{m}}_{\pi (i)}$. As the ${\tilde{\theta }}_{i}$ are IID and independent of ${\tilde{w}}_{i}$, *π* is uniformly distributed over the set of permutations of $\left\{1,\ldots ,N_{\alpha }\right\}$. The vector $\left(m_{1},\ldots ,m_{N_{\alpha }}\right)$ corresponds to the sizes of the partition $\Pi_{2D_{\alpha }^{*}}$ in exchangeable random order (Pitman (2006), equation (2.7), page 39), and, for *r* ∈ {2,4,6,…},

(71) $$\begin{array}{cc} & \text{Pr}\{D_{\alpha }^{*}=r/2,\left(m_{1},\ldots ,m_{N_{\alpha }}\right),{\left(w_{i}\in dw_{i}\right)}_{i=1,\ldots N_{\alpha }},w_{*}\in dw_{*}\}\\ & =\frac{r!}{\left(r/2\right)!N_{\alpha }!\prod_{i=1}^{N_{\alpha }}m_{i}!}\exp\left\{-{\left(w_{*}+\sum_{i=1}^{N_{\alpha }}w_{i}\right)}^{2}\right\}G_{\alpha }^{*}\left(dw_{*}\right)\alpha^{N_{\alpha }}\prod_{i=1}^{N_{\alpha }}w_{i}^{m_{i}}\rho \left(dw_{i}\right). \end{array}$$

## Appendix F Details on the Markov chain Monte Carlo algorithms

The undirected graph sampler that was outlined in Section 7.2 iterates as follows.

*Step 1*: update $w_{1:N_{\alpha }}$ given the rest with HMC sampling.
*Step 2*: update (*α*,*σ*,*τ*,*w*_*_) given the rest by using a Metropolis–Hastings step.
*Step 3*: update the latent counts ${\overset{¯}{n}}_{ij}$ given the rest by using either the full conditional or a Metropolis–Hastings step.

### F.1. Step 1: update of $w_{1:N_{\alpha }}$

We use an HMC update for $w_{1:N_{\alpha }}$ via an augmented system with momentum variables *p*. See Neal (2011) for an overview. Let *L*⩾1 be the number of leapfrog steps and *ɛ*>0 the step size. For conciseness, we write

$$U^{\prime }\left(w_{1:N_{\alpha }},w_{*},\phi \right)=\nabla_{\omega_{1:N_{\alpha }}}\log\left\{p\left(\omega_{1:N_{\alpha }},w_{*},\phi |D_{\alpha }\right)\right\}{|}_{w_{1:N_{\alpha }},w_{*},\phi }$$

the gradient of the log‐posterior in equation (45). The algorithm proceeds by first sampling momentum variables as

(72) $$p∼N(0,I_{N_{\alpha }}).$$

The Hamiltonian proposal $q({\tilde{w}}_{1:N_{\alpha }},\tilde{p}|w_{1:N_{\alpha }},p)$ is obtained by the following leapfrog algorithm (for simplicity of exposition, we omit indices $1:N_{\alpha }$). Simulate *L* steps of the discretized Hamiltonian via

$$\begin{array}{cc} & {\tilde{p}}^{\left(0\right)}=p+\frac{\varepsilon }{2}U^{\prime }\left(w,w_{*},\phi \right),\\ & {\tilde{w}}^{\left(0\right)}=w \end{array}$$

and, for *l*=1,…,*L*−1,

$$\begin{array}{cc} & \log\left({\tilde{w}}^{\left(l\right)}\right)=\log\left({\tilde{w}}^{\left(l-1\right)}\right)+\varepsilon {\tilde{p}}^{\left(l-1\right)},\\ & {\tilde{p}}^{\left(l\right)}={\tilde{p}}^{\left(l-1\right)}+\varepsilon U^{\prime }\left({\tilde{w}}^{\left(l\right)},w_{*},\phi \right) \end{array}$$

and finally set

$$\begin{array}{cc} & \log\left(\tilde{w}\right)=\log\left({\tilde{w}}^{\left(L-1\right)}\right)+\varepsilon {\tilde{p}}^{\left(L-1\right)},\\ & \tilde{p}=-\left\{{\tilde{p}}^{\left(L-1\right)}+\frac{\varepsilon }{2}U^{\prime }\left(\tilde{w},w_{*},\phi \right)\right\}\\ & \tilde{w}={\tilde{w}}^{\left(L\right)}. \end{array},$$

Accept the proposal $\left(\tilde{w},\tilde{p}\right)$ with probability min(1,*r*) with

$$\begin{array}{cc} r & =\frac{\left(\prod_{i=1}^{N_{\alpha }}{\tilde{w}}_{i}^{m_{i}}\right)\exp\left\{-{\left(\sum_{i=1}^{N_{\alpha }}{\tilde{w}}_{i}+w_{*}\right)}^{2}\right\}\prod_{i=1}^{N_{\alpha }}{\tilde{w}}_{i}\rho \left({\tilde{w}}_{i}\right)}{\left(\prod_{i=1}^{N_{\alpha }}w_{i}^{m_{i}}\right)\exp\left\{-{\left(\sum_{i=1}^{N_{\alpha }}w_{i}+w_{*}\right)}^{2}\right\}\prod_{i=1}^{N_{\alpha }}w_{i}\rho (w_{i})}\exp\left\{-\frac{1}{2}\sum_{i=1}^{N_{\alpha }}\left({\tilde{p}}_{i}^{2}-p_{i}^{2}\right)\right\}\\ & \;=\left\{\prod_{i=1}^{N_{\alpha }}{\left(\frac{{\tilde{w}}_{i}}{w_{i}}\right)}^{m_{i}-\sigma }\right\}\exp\left\{-{\left(\sum_{i=1}^{N_{\alpha }}{\tilde{w}}_{i}+w_{*}\right)}^{2}+{\left(\sum_{i=1}^{N_{\alpha }}w_{i}+w_{*}\right)}^{2}-\tau \left(\sum_{i=1}^{N_{\alpha }}{\tilde{w}}_{i}-\sum_{i=1}^{N_{\alpha }}w_{i}\right)\right\}\exp\left\{-\frac{1}{2}\sum_{i=1}^{N_{\alpha }}\left({\tilde{p}}_{i}^{2}-p_{i}^{2}\right)\right\}. \end{array}$$

### F.2. Step 2: update of *w*_*_,*α*,*σ* and *τ*

Let $g_{\alpha ,\sigma ,\tau }^{*}$ denote the density of $G_{\alpha ,\sigma ,\tau }^{*}$ with respect to the reference measure *λ*_*α*_+*δ*_0_, where we recall that *λ*_*α*_ denotes the Lebesgue measure over [0,*α*]. For our Metropolis–Hastings step, we propose ($\tilde{\alpha },\tilde{\sigma },\tilde{\tau }$,${\tilde{w}}_{*}$) from $q(\tilde{\alpha },\tilde{\sigma },\tilde{\tau },{\tilde{w}}_{*}|\alpha ,\sigma ,\tau ,w_{*})$ and accept with probability min(1,*r*) where

(73) $$r=\frac{\exp\left\{-{\left(\sum_{i=1}^{N_{\alpha }}w_{i}+{\tilde{w}}_{*}\right)}^{2}\right\}}{\exp\left\{-{\left(\sum_{i=1}^{N_{\alpha }}w_{i}+w_{*}\right)}^{2}\right\}}\left\{\prod_{i=1}^{N_{\alpha }}\frac{\rho (w_{i}|\tilde{\sigma },\tilde{\tau })}{\rho (w_{i}|\sigma ,\tau )}\right\}\frac{g_{\tilde{\alpha },\tilde{\sigma },\tilde{\tau }}^{*}\left({\tilde{w}}_{*}\right)}{g_{\alpha ,\sigma ,\tau }^{*}\left(w_{*}\right)}\frac{p(\tilde{\alpha },\tilde{\sigma },\tilde{\tau })}{p(\alpha ,\sigma ,\tau )}\frac{q(\alpha ,\sigma ,\tau ,w_{*}|\tilde{\alpha },\tilde{\sigma },\tilde{\tau },{\tilde{w}}_{*})}{q(\tilde{\alpha },\tilde{\sigma },\tilde{\tau },{\tilde{w}}_{*}|\alpha ,\sigma ,\tau ,w_{*})}.$$

We shall use the proposal

$$q\left(\tilde{\alpha },\tilde{\sigma },\tilde{\tau },{\tilde{w}}_{*}|\alpha ,\sigma ,\tau ,w_{*}\right)=q\left(\tilde{\tau }|\tau \right)q\left(\tilde{\sigma }|\sigma \right)q\left(\tilde{\alpha }|\tilde{\sigma },\tilde{\tau },w_{*}\right)q\left({\tilde{w}}_{*}|\tilde{\alpha },\tilde{\sigma },\tilde{\tau },w_{*}\right)$$

where

(74) $$\begin{array}{cc} & q\left(\tilde{\tau }|\tau \right)=\text{lognormal}\left\{\tilde{\tau };\log\left(\tau \right),\sigma_{\tau }^{2}\right\},\\ & q\left(\tilde{\sigma }|\sigma \right)=\text{lognormal}\left\{1-\tilde{\sigma };\log\left(1-\sigma \right),\sigma_{\tau }^{2}\right\},\\ & q\left(\tilde{\alpha }|\tilde{\sigma },\tilde{\tau },w_{*}\right)=\text{gamma}\left\{\tilde{\alpha };N_{\alpha },\psi_{\tilde{\sigma },\tilde{\tau }}\left(2\sum_{i}w_{i}+2w_{*}\right)\right\},\; \end{array}$$

(75) $$q\left({\tilde{w}}_{*}|\tilde{\alpha },\tilde{\sigma },\tilde{\tau },w_{*}\right)=g_{\tilde{\alpha },\tilde{\sigma },\tilde{\tau }+2\Sigma_{i}w_{i}+2w_{*}}^{*}\left({\tilde{w}}_{*}\right).$$

The choice of the proposal for *w*_*_ and *α* is motivated as follows. From equations (71) and (43), the conditional density of (*α*,*w*_*_) given the rest is given by

$$p\left(\alpha ,w_{*}|\text{rest}\right)\propto \alpha^{N_{\alpha }-1}g_{\alpha ,\sigma ,\tau }^{*}\left(w_{*}\right)\exp\left\{-{\left(w_{*}+\sum_{i}w_{i}\right)}^{2}\right\}$$

which is not of a standard form because of the square in the exponential. Motivated by a first‐order Taylor approximation around the current MCMC value *w*_*_,

$${\left({\tilde{w}}_{*}+\sum_{i}w_{i}\right)}^{2}≃{\left(w_{*}+\sum_{i}w_{i}\right)}^{2}+2\left({\tilde{w}}_{*}-w_{*}\right)\left(w_{*}+\sum_{i}w_{i}\right),$$

we use a proposal

$$q\left(\tilde{\alpha },{\tilde{w}}_{*}|\tilde{\sigma },\tilde{\tau },w_{*}\right)\propto {\tilde{\alpha }}^{N_{\alpha }-1}g_{\tilde{\alpha },\tilde{\sigma },\tilde{\tau }}^{*}\left({\tilde{w}}_{*}\right)\exp\left\{-2{\tilde{w}}_{*}\left(w_{*}+\sum_{i}w_{i}\right)\right\}$$

which corresponds to the product of the proposals (74) and (75). The proposal for *w*_*_ can be written as an exponential tilting of the probability density function $g_{\tilde{\alpha },\tilde{\sigma },\tilde{\tau }}^{*}\left({\tilde{w}}_{*}\right):$

$$g_{\tilde{\alpha },\tilde{\sigma },\tilde{\tau }+2\Sigma w_{i}+2w_{*}}^{*}\left({\tilde{w}}_{*}\right)=\frac{\exp\left\{-2{\tilde{w}}_{*}\left(\sum w_{i}+w_{*}\right)\right\}g_{\tilde{\alpha },\tilde{\sigma },\tilde{\tau }}^{*}\left({\tilde{w}}_{*}\right)}{\exp\{-\tilde{\alpha }\psi_{\tilde{\sigma },\tilde{\tau }}\left(2\sum_{i}w_{i}+2w_{*}\right)\}}$$

which will allow the terms involving the intractable probability density function *g* ^*^ to cancel in the Metropolis–Hastings ratio (73). *g* ^*^ is either a gamma density (*σ*=0), a Poisson mixture of gamma densities (*σ*<0) or an exponentially tilted stable density (*σ*>0) for which efficient samplers exist (Devroye, 2009; Hofert, 2011).

Under the improper priors (43), the acceptance probability reduces to having

$$\begin{array}{cc} r= & \exp\left\{-{\left(\sum_{i=1}^{N_{\alpha }}w_{i}+{\tilde{w}}_{*}\right)}^{2}+{\left(\sum_{i=1}^{N_{\alpha }}w_{i}+w_{*}\right)}^{2}\right\}\exp\left\{-\left(\tilde{\tau }-\tau +2w_{*}-2{\tilde{w}}_{*}\right)\sum_{i=1}^{N_{\alpha }}w_{i}\right\}\\ & \times {\left(\prod_{i=1}^{N_{\alpha }}w_{i}\right)}^{-\tilde{\sigma }+\sigma }{\left\{\frac{\Gamma \left(1-\sigma \right)\psi_{\sigma ,\tau }\left(2{\tilde{w}}_{*}+2\sum_{i}w_{i}\right)}{\Gamma \left(1-\tilde{\sigma }\right)\psi_{\tilde{\sigma },\tilde{\tau }}\left(2w_{*}+2\sum_{i}w_{i}\right)}\right\}}^{N_{\alpha }}. \end{array}$$

### F.3. Step 3: update of the latent variables ${\overset{¯}{n}}_{ij}$

Concerning the latent ${\overset{¯}{n}}_{ij}$, the conditional distribution is a truncated Poisson distribution (44) from which we can sample directly. An alternative strategy, which may be more efficient for a large number of edges, is to use a Metropolis–Hastings random‐walk proposal.
